# Catalytic Reduction of Cyanide to Ammonia and Methane at a Mononuclear Fe Site

**DOI:** 10.1021/jacs.3c12395

**Published:** 2024-02-16

**Authors:** Christian M. Johansen, Jonas C. Peters

**Affiliations:** Division of Chemistry and Chemical Engineering, California Institute of Technology, Pasadena, California 91125, United States

## Abstract

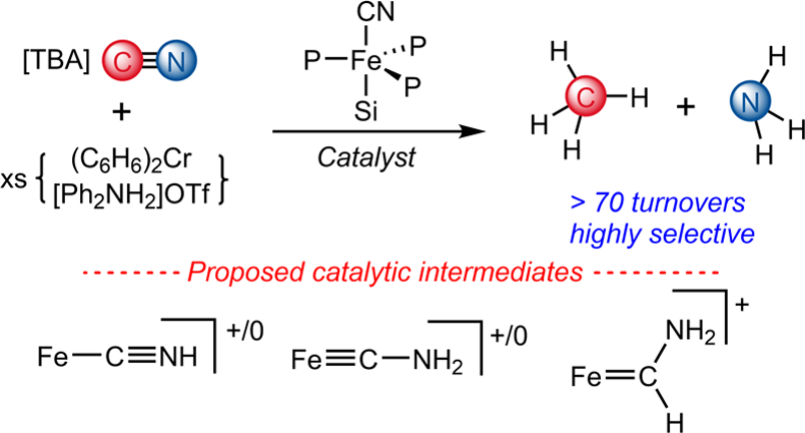

Nitrogenase enzymes catalyze nitrogen reduction (N_2_R) to ammonia and also the reduction of non-native substrates, including the 7H^+^/6e^–^ reduction of cyanide to CH_4_ and NH_3_. CN^–^ and N_2_ are isoelectronic, and it is hence fascinating to compare the mechanisms of synthetic Fe catalysts capable of both CN^–^ and N_2_ reduction. Here, we describe the catalytic reduction of CN^–^ to NH_3_ and CH_4_ by a highly selective (P_3_^Si^)Fe(CN) catalyst (P_3_^Si^ represents a tris(phosphine)silyl ligand). Catalysis is driven in the presence of excess acid ([Ph_2_NH_2_]OTf) and reductant ((C_6_H_6_)_2_Cr), with turnover as high as 73 demonstrated. This catalyst system is also modestly competent for N_2_R and structurally related to other tris(phosphine)Fe-based N_2_R catalysts. The choice of catalyst and reductant is important to observe high yields. Mechanistic studies elucidate several intermediates of CN^–^ reduction, including iron isocyanides (P_3_^Si^FeCNH^+/0^) and terminal iron aminocarbynes (P_3_^Si^FeCNH_2_^+/0^). Aminocarbynes are isoelectronic to iron hydrazidos (Fe=N–NH_2_^+/0^), which have been invoked as selectivity-determining intermediates of N_2_R (NH_3_ versus N_2_H_4_ products). For the present CN^–^ reduction catalysis, reduction of aminocarbyne P_3_^Si^FeCNH_2_^+^ is proposed to be rate but not selectivity contributing. Instead, by comparison with the reactivity of a methylated aminocarbyne analogue (P_3_^Si^FeCNMe_2_), and associated computational studies, formation of a Fischer carbene (P_3_^Si^FeC(H)(NH_2_)^+^) intermediate that is on path for either CH_4_ and NH_3_ (6 e^–^) or CH_3_NH_2_ (4 e^–^) products is proposed. From this carbene intermediate, pathways to the observed CH_4_ and NH_3_ products (distinct from CH_3_NH_2_ formation) are considered to compare and contrast the (likely) mechanism/s of CN^–^ and N_2_ reduction.

## Introduction

Nitrogenases catalyze nitrogen reduction to ammonia (N_2_R) as well as the reductive protonation of non-native substrates,^1−4^ including cyanide (CN^–^).^5−13^ These are mechanistically fascinating bioorganometallic transformations which, for the case of CN^–^ (and CO/CO_2_ as well), may involve metal-to-carbon intermediates such as alkyls, carbenes, and carbynes/carbides that are conceptually related to posited intermediates of N_2_R (e.g., NNH, NNH_2_, NH).

Whereas substantial attention from the synthetic community has been directed toward functional N_2_R models with associated mechanistic studies,^14−16^ there has been only limited attention paid to catalytic cyanide reduction by comparison.^17−22^ Given potential mechanistic parallels between catalytic N_2_ and CN^–^ reduction, including an isolobal relationship between aminocarbynes (e.g., M≡CNR_2_)^23−26^ and their hydrazido (M=NNR_2_) counterparts,^27−29^ mechanistically well-defined CN^–^ reduction catalysts present an attractive target for further study. In contrast to terminal hydrazido systems, the reductive protonation of terminal carbynes to liberate products (e.g., CH_4_/NH_3_) has rarely been observed.^25,30−33^ Indeed, catalytic transformations involving *bona fide* carbyne intermediates, outside of the scope of metathesis reactions,^34,35^ are essentially without precedent.

**Figure 1:**
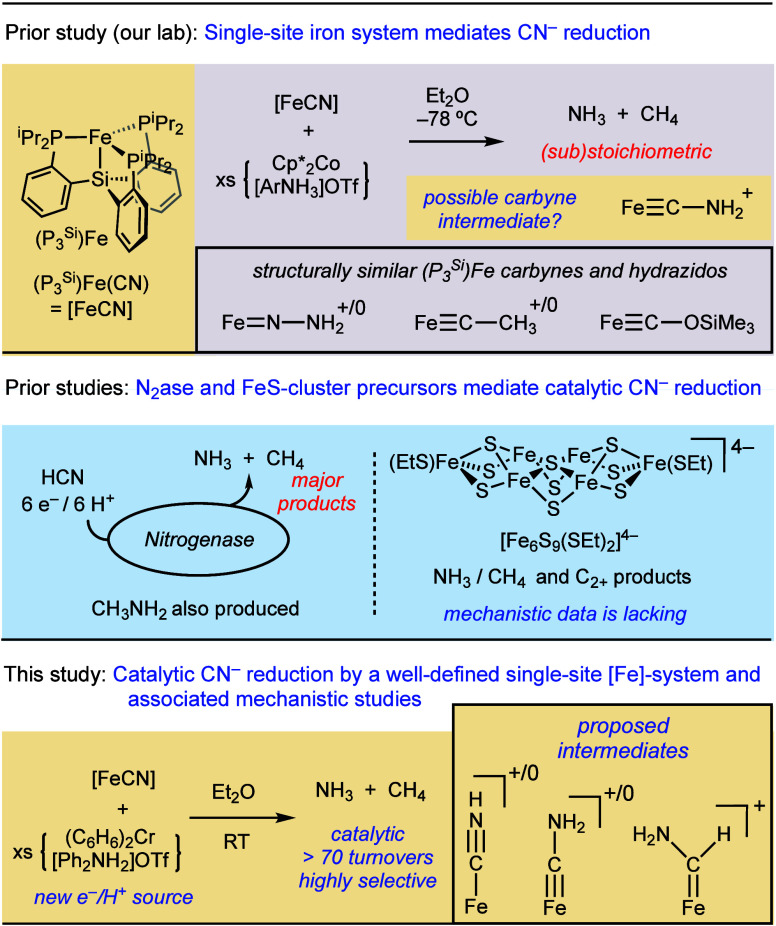
Summary of prior studies on stoichiometric and catalytic cyanide reductions mediated by iron complexes as a context for this study.

Toward these objectives, our lab reported in 2016 a single-site iron model system capable of mediating the (sub)stoichiometric reductive protonation of CN^–^ to CH_4_ and NH_3_.^25^ We also characterized a number of species as plausible intermediates of the overall transformation, most notably the carbyne complex (P_3_^Si^)Fe(CNH_2_)^+^ (P_3_^Si^ represents a tris(phosphino)silyl ligand; Figure 1, top).^25^ The product distribution observed mimics that of ATP-dependent cyanide reduction by nitrogenases (Figure 1, middle), where the major observed products under most conditions studied are methane and ammonia (6 e^–^ reduction); methylamine (H_3_CNH_2_; 4 e^–^ reduction) and methylenimine (H_2_C=NH; 2 e^–^ reduction) can also be observed as minor products, along with trace ethane and ethylene.^6−9^

Several synthetic Fe–S clusters have also been shown to catalyze cyanide reduction and exhibit substantially higher selectivities for C–C coupled products than has been observed with nitrogenase enzymes as the catalysts (Figure 1, middle).^10−12,20−22^ Catalytically relevant species in transformations employing such clusters as precatalysts are ill-defined, and to date, associated mechanistic information has not been forthcoming.

Against this backdrop, we have sought conditions for *catalytic* cyanide reduction via our well-defined (P_3_^Si^)Fe system, ideally manifesting product distributions akin to nitrogenases (chiefly favoring the C_1_ products CH_4_ and CH_3_NH_2_) and amenable to mechanistic scrutiny. This study presents our findings (Figure 1, bottom).

**Figure 2:**
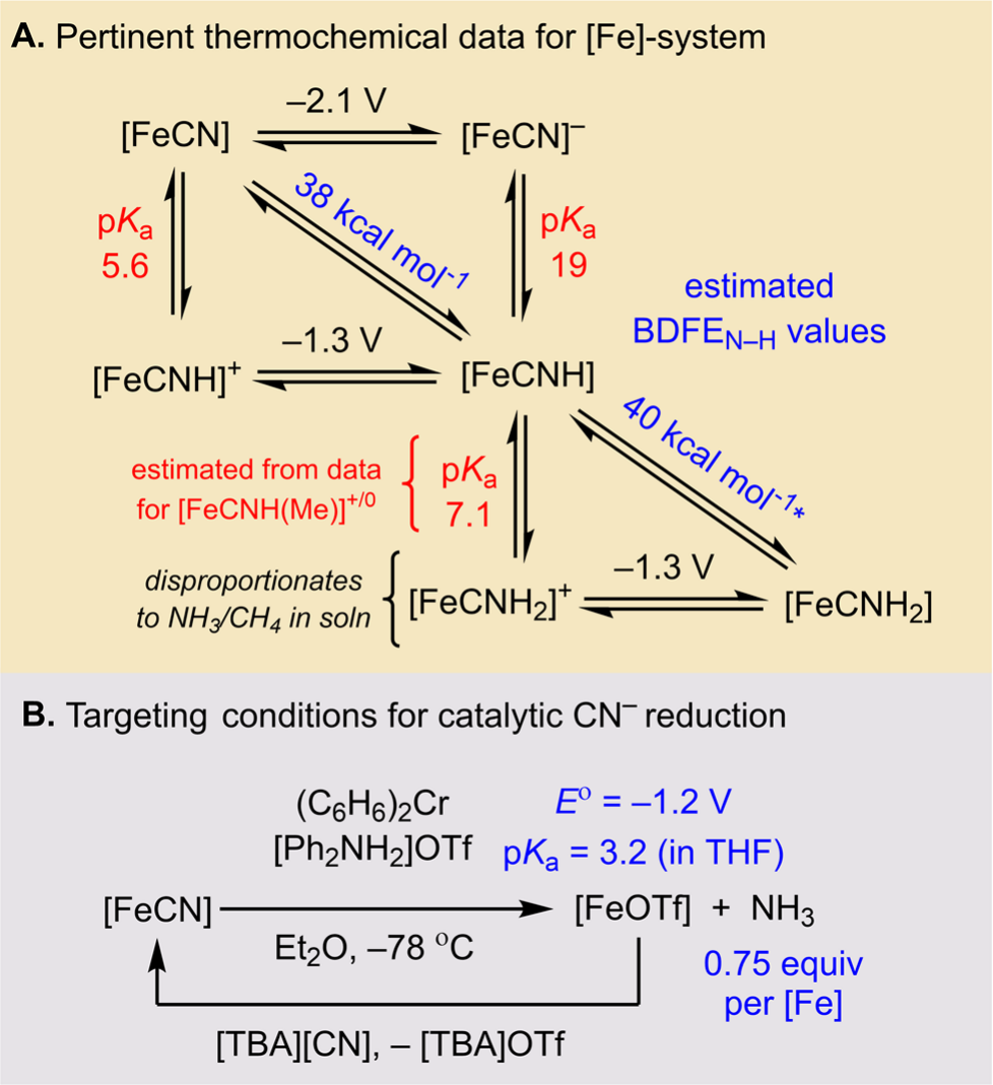
(A) Relevant, previously measured, thermochemical data (values in THF at 25 °C; *E*° in referenced to Fc^+/0^). (B) Exploring new conditions for the reductive protonation of [FeCN].

Guided by measured and estimated thermochemical parameters (Figure 2a),^36^ we show herein that the iron complex (P_3_^Si^)Fe(CN) (abbreviated as [FeCN]) efficiently catalyzes cyanide reduction in the presence of acids and reductants. By employing a combination of synthetic, ^57^Fe Mössbauer, optical, and theoretical studies, we outline a mechanistic scheme for the catalytic cycle, which can be juxtaposed with that of catalytic nitrogen fixation mediated by analogous iron complexes.

## Results

### Canvassing Conditions for More Efficient [FeCN] Reduction

To target the catalytic reduction of CN^–^, we sought conditions for the proton-coupled reduction of [FeCN] to produce NH_3_/CH_4_ (or CH_3_NH_2_) and an [Fe] byproduct that might re-enter a catalytic cycle. In our original report,^25^ we described the proton-coupled reduction of [FeCN] using excess [^2,5-Cl^PhNH_3_]OTf and Cp*_2_Co (p*K*_a_ 4.5 for [^2,5-Cl^PhNH_3_]OTf in THF; all p*K*_a_’s reported in THF;^36^*E*° = −1.9 V for Cp*_2_Co; all redox potentials are reported in THF and referenced to Fc^+/0^).^37^ Such reaction mixtures invariably afforded low yields of NH_3_/CH_4_ (Figure 1a) despite being effective for catalytic N_2_R.^38^

Curiously, in our original study, we had observed that the cationic aminocarbyne, [FeCNH_2_]OTf, prepared via double protonation of [FeCN][Na(12-c-4)_2_],^25^ decays upon warming to liberate 0.09 equiv NH_3_/Fe and 0.07 CH_4_/Fe (Figure 2), with [FeCNH]OTf and [FeOTf] as the major Fe products. This NH_3_ yield represents ∼50% of that theoretically possible for a disproportionation reaction assuming a stoichiometry of five equivalents of [FeCNH_2_]OTf providing five H atom equivalents ([FeCNH_2_]^+^→ [FeCNH]^+^ + H^+^/e^–^) to reduce one equivalent [FeCNH_2_]OTf to NH_3_ and CH_4_ (eq 1).
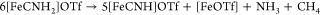
1

Based on thermochemical data (Figure 2a),^36^ removal of a H^+^/e^–^ pair from [FeCNH_2_]^+^ is equivalent to removal of 1H^+^/1e^–^ from an acid/reductant pair with p*K*_a_ ∼ 7 and *E*° ∼ −1.3 V. Reagents suiting these values would be significantly milder than [^2,5-Cl^PhNH_3_]OTf and Cp*_2_Co. Hence, once [FeCNH_2_]^+^ is formed *in situ* via reductive protonation of [FeCN] (p*K*_a_ = 5.6), comparatively mild reagents should drive net CN^–^ reduction. Because [FeCN] can be converted to [FeCNH_2_]OTf with a reductant strength of *E*° ≈ −1.3 V, we deduced that the 7H^+^/6e^–^ reduction of [FeCN] should be accessible with reductants at *E*° ≈ −1.3 V.

Gratifyingly, [FeCN] was stirred with (C_6_H_6_)_2_Cr (*E*° = −1.2 V, Figure S23)^39^ and [Ph_2_NH_2_]OTf (p*K*_a_ 3.2)^40^ in Et_2_O at −78 °C and then the reaction mixture was allowed to warm to RT overnight yielding 0.75 equiv NH_3_/Fe (75% yield per reductant) with [FeOTf] as the major Fe product (Figure 2b and Figure S9). Moreover, it was established that [FeOTf] reacts cleanly with excess [TBA][CN] to reform [FeCN], setting the stage for catalysis (Figure S10).

### Catalytic CN^–^ Reduction

Thus, using [FeCN] (0.72 mM) as a precatalyst, in a reaction mixture containing 140 equiv [TBA][CN] (100 mM), 480 equiv [Ph_2_NH_2_]OTf, and 360 equiv (C_6_H_6_)_2_Cr in Et_2_O at 25 °C, yielded 28 ± 5 equiv NH_3_/Fe after 80 min (Figure 2c and Table 1, entry 1).

**Table 1:**
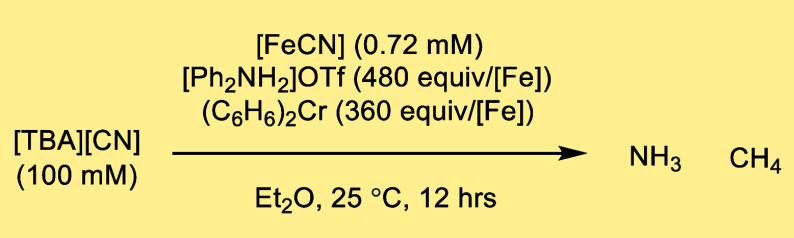
Results for the Catalytic Reduction of CN^–^ to Ammonia

entry	change from standard conditions	NH_3_ (equiv/Fe)	CH_4_ (equiv/Fe)	yield per reductant (%)^a^
1	none	28 ± 5	25 ± 4	47 ± 8
2	–78 °C→25 °C	33 ± 6	33 ± 3	55 ± 10
3	–20 °C→25 °C	35 ± 8		58 ± 13
4	0 °C→25 °C	26 ± 2		43 ± 3
5	2.9 mM [FeCN]^b^	9.7 ± 0.2		65 ± 1
6	0.15 mM [FeCN]^b^	73 ± 4		24 ± 1
7	no [FeCN]^b^	<0.4	<0.3	<1
8	no [TBA][CN]^b^	0.7		1.2
9	8.0 mM FeCl_2_ as cat.^b^	0.3		5.5
10	8.0 mM CrCl_2_ as cat.^b^	<0.05		<1
11	2.9 mM (PhB^iPr^_3_) FeBr as cat.^b^	1.4 ± 0.7	1.0 ± 0.1	12 ± 3^c^
12	2.9 mM (P_3_^B^)Fe[BAr^F^_4_] as cat.^b^	2.3 ± 0.3	1.6	15.6 ± 0.6
13	Cp_2_Co instead of (C_6_H_6_)_2_Cr	2.8 ± 0.8		12 ± 4
14	Cp*_2_Cr instead of (C_6_H_6_)_2_Cr	13.5 ± 3		32 ± 6
15	[FeOTf] as cat.	32		53
16	reloaded catalysis^d^	4.1 ± 1.0		6.8 ± 1.7

a Yields assume net 6 e^–^ reduction to generate NH_3_.

b Catalysis initiated at −78 °C and then allowed to warm gradually to 25 °C (total reaction time of 12 h).

c This yield includes 0.6 ± 0.2 equiv CH_3_NH_2_.

d After 80 min of catalysis under standard conditions (entry 1), soluble Fe species were extracted into Et_2_O and then re-exposed to the standard catalytic conditions.

Using these same catalytic conditions, we also analyzed the gaseous products. CH_4_ was observed as the major reduced carbon product, with a yield of 25 ± 4 equiv of CH_4_/Fe, consistent with a net 7H^+^/6e^–^ reduction of CN^–^ (eq 2; yield based on consumed (C_6_H_6_)_2_Cr is 47 ± 8%).
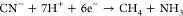
2

Under these conditions, trace C_2_ products ethylene and ethane were also identified (0.4 equiv C_2_H_4_ and 0.3 equiv C_2_H_6_ per Fe). These products correspond to 10H^+^/8e^–^ and 12H^+^/10e^–^ reductions of CN^–^. Combined, these C_2_ evolving reactions accounted for less than 2% of the consumed reductant. Hence, the [FeCN] catalyst is nearly quantitatively selective for C_1_ products, as is observed via the nitrogenase enzyme.^6,7^ H_2_ accounts for most of the remaining reducing equivalents (yield based on (C_6_H_6_)_2_Cr: 29 ± 11%). Neither CH_3_NH_2_ (4e^–^ product) or CH_2_NH (2e^–^) was detected, regardless of initial temperature, using [FeCN] as a catalyst.

Curiously, whereas synthetic iron catalysts for N_2_R have shown highest efficiency at low temperatures due to mitigated HER (hydrogen evolution reaction) and entropically favored N_2_ binding,^41^ no such advantage is observed for catalytic cyanide reduction by [FeCN] (entries 2–4). Instead, background HER via the combination of this reductant and acid is comparatively slow (*vide infra*). Also, CN^–^ binds favorably to [Fe(II)] at RT. For reactions started at −78 °C, catalytic turnover is slow, reflecting a slow OTf^–^ for CN^–^ metathesis step needed to turn the system over (*vide infra*); most of the observed catalysis occurs as the reaction is warmed. For a catalytic reaction run at −20 °C and quenched after 20 min, 1.7 equiv of NH_3_ was detected, demonstrating that catalytic turnover occurs at this temperature but is relatively slow.

Increasing the catalyst loading to 2.9 mM (entry 5) modestly increased the NH_3_ yield relative to reductant present (65 ± 1%). Lowering the catalyst loading (0.15 mM; entry 6) improved the TON for produced NH_3_ (73 ± 4 equiv) but led to a corresponding drop in yield per (C_6_H_6_)_2_Cr (24 ± 1%).

A catalyst-free reaction yielded no detectable NH_3_, CH_4_, or other gaseous carbon products (entry 7). This conclusion is further supported by experiments with [TBA][^13^CN] as the cyanide source. ^13^C NMR spectroscopy of catalytic runs using [TBA][^13^CN] confirmed the formation of ^13^CH_4_ and consumption of ^13^CN^–^ (Figures S5 and S6). By contrast, a corresponding catalyst-free reaction (under otherwise identical conditions) showed negligible consumption of ^13^CN^–^ and no observable ^13^CH_4_. These observations collectively establish that the Fe catalyst is required for consumption of substrate and responsible for the NH_3_ and CH_4_ products.

Catalysis run in the absence of [TBA][CN] produced 0.7 equiv of NH_3_, with [FeC^15^N] used to demonstrate that this NH_3_ arose solely from precatalyst reduction and not N_2_R (entry 8, Figure S4).

The nature of the phosphine-ligated iron catalyst appears to be critical. FeCl_2_ instead of [FeCN] produced only 0.3 equiv of NH_3_ under the standard conditions (entry 9), and CrCl_2_ instead of [FeCN] produced no detectable NH_3_ (entry 10). The tris(phosphino)iron complexes (P_3_^B^)Fe[BAr^F^_4_]^16^ and (PhBP^iPr^_3_)FeBr^42^ (P_3_^B^ represents a trisphosphine borane ligand; PhBP^iPr^_3_ represents a trisphosphine borate ligand) showed very moderate activity as (pre)catalysts compared to [FeCN] (entries 11–12). Curiously, for (PhBP^iPr^_3_)FeBr, a small amount of methylamine, CH_3_NH_2_ (0.6 ± 0.2), was detected as a product. These iron phosphine precatalysts produce CH_4_ as the major hydrocarbon product but with a lower selectivity. The ratio of C_2_/C_1_ products produced is 0.16 and 0.11 for (P_3_^B^)Fe[BAr^F^_4_] and (PhBP^iPr^_3_)FeBr, respectively (Table S3), compared to 0.02 for [FeCN].

While (C_6_H_6_)_2_Cr is the favored reductant for CN^–^ reduction, other reductants including Cp_2_Co (*E*° = −1.3 V, Figure S25; entry 13) and Cp*_2_Cr (*E*° = −1.5 V, Figure S27; entry 14) were modestly competent. The low yields for these reductants do not appear to correlate with the reduction potential of the chemical reductant. Instead, we attribute the strong attenuation in yield to enhanced background HER. Accordingly, we find that the rate of reaction of each reductant independently with [Ph_2_NH_2_]OTf (to produce H_2_), as measured by cyclic voltammetry, inversely correlates with the NH_3_ TON observed in a catalytic run when CN^–^ is present under the standard conditions (see Section S8.2 for details).

To summarize, we have established a highly selective catalytic system for NH_3_ and CH_4_ production via reductive protonation of CN^–^; the choice of catalyst ([FeCN]) and reductant ((C_6_H_6_)_2_Cr) is crucial for observing high turnover and significant yields.

### Mechanistic Studies

**Scheme 1:**
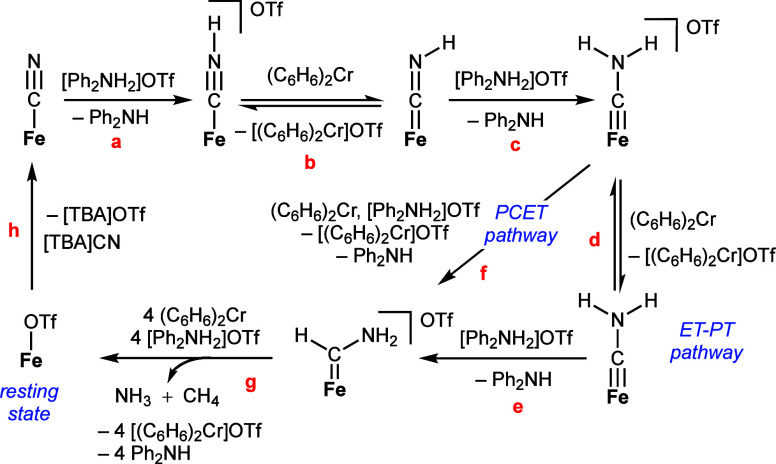
Proposed Mechanism for CN^–^ Reduction to NH_3_ and CH_4_ as Catalyzed by [FeCN]

Scheme 1 provides a working outline for the catalytic CN^–^ reduction cycle starting from [FeCN], emphasizing the early intermediates of the cycle. To guide the following discussion, summary remarks concerning a plausible pathway are as follows: [FeCN] is first protonated (step a) to form independently characterized [FeCNH]^+^,^25^ which is then reduced (to [FeCNH]; step b) and protonated (step c) to afford the independently characterized aminocarbyne, [FeCNH_2_]^+^.^25^ [FeCNH_2_]^+^ is in redox equilibrium with [FeCNH_2_] in the presence of (C_6_H_6_)_2_Cr (step d). These carbyne intermediates are suggested to be rate-contributing to overall CN^–^ reduction (see below). Along the ET-PT pathway, [FeCNH_2_] can be protonated to form a posited carbene intermediate (step e), [FeC(H)(NH_2_)]^+^. This carbene is modeled via the independent generation of its methylated analogue, [FeC(H)(NMe_2_)]^+^, via the protonation of [FeCNMe_2_] (see below). This observation and computational evidence each lends support to C–H bond formation to produce [FeC(H)(NH_2_)]^+^ during catalysis. A direct PCET pathway from [FeCNH_2_]^+^ to [FeC(H)(NH_2_)]^+^ is also plausible (step f). Finally, a series of downstream (as yet undefined), facile reductive protonation steps of [FeC(H)(NH_2_)]^+^ are proposed to release NH_3_ and CH_4_ along with [FeOTf] (step g); the latter is returned to [FeCN] via metathesis with [TBA][CN], a step (step (h)) that is turnover limiting.

### Probing Catalyst Resting State and Deactivation

To probe speciation during catalysis, we prepared [^57^FeCN] to facilitate monitoring the catalysis by ^57^Fe Mössbauer spectroscopy via low-temperature quenching of catalytic runs initiated at 25 °C. Related studies proved insightful for N_2_R catalysis by related Fe systems.^40,43^

Freeze-quenching (77 K) the catalysis after 1 min at 25 °C, we found [^57^FeOTf] as the sole iron species present (see Section S6.2).^44^ This result points to [FeOTf] as the catalyst resting state with metathesis step (h) being turnover limiting. Consistent with this observation, [FeOTf] performs analogous to [FeCN] as a catalyst (Table 1, entry 15). Freeze-quenched snapshots at later reaction times show attenuation in the signal for [^57^FeOTf] and the growth of unknown iron species. After 80 min, all of the [^57^FeOTf] has been consumed; the remaining iron species showed poor activity following extraction and (re)subjection to catalytic conditions at 25 °C with fresh acid, reductant and [TBA][CN], yielding only an additional 4.1 ± 1 equiv NH_3_ (entry 16). NMR analysis of the postcatalysis mixture revealed evidence of a diamagnetic iron hydride (possibly [Fe(H)(NHPh_2_)]) with the (P_3_^Si^)Fe platform intact, as well as free Si(H)P_3_ (Figure S13). Relatedly, iron hydrides (e.g., [Fe(H)(N_2_)]) have been shown to be off-cycle sinks during catalytic N_2_R.^43,45,46^

While our mechanistic studies have focused on the most efficient catalyst, [FeCN], initial studies of the reactivity of (P_3_^B^)Fe[BAr^F^_4_] and (PhBP^iPr^_3_)FeBr suggest that these less efficient precatalysts are also less stable to excess CN^–^. When reacted with 20 equiv [TBA][CN] in Et_2_O (in the absence of acid or reductant), free phosphine is observed, indicating partial demetalation as a pathway for deactivation, offering a plausible reason for the lower turnover numbers (Figures S14 and S15).

### Early N–H Bond Forming Steps

Since metathesis to produce [FeCN] from [FeOTf] appeared to be turnover limiting, we turned to stoichiometric experiments to probe the role(s) of early intermediates of reductive protonation in this catalysis.

**Figure 3:**
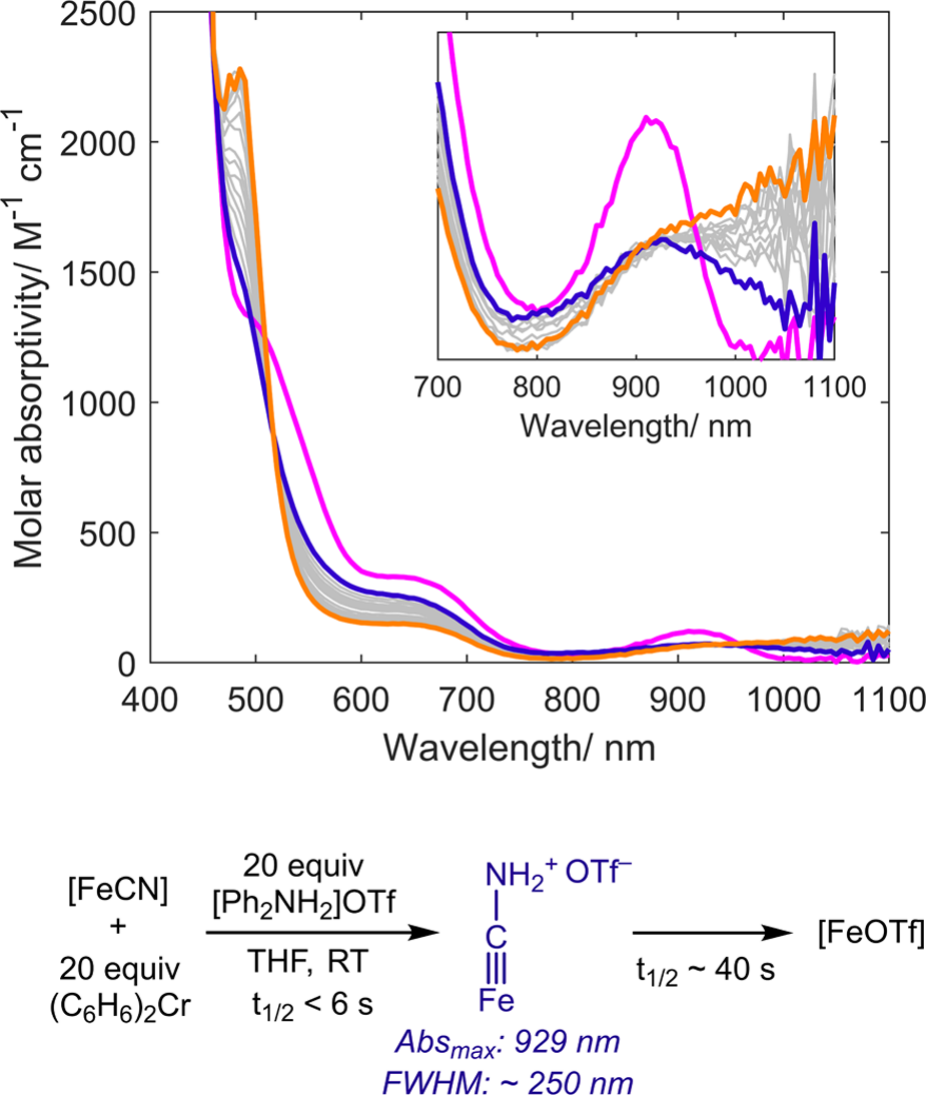
UV–vis data showing the *in situ* formation of [FeCNH_2_]OTf (blue trace, 6 s after acid addition) in the reduction of [FeCN] (pink trace) to [FeOTf] (orange trace, 240 s after acid addition).

Exposing a solution containing a mixture of [FeCN] and a large excess (20 equiv) of (C_6_H_6_)_2_Cr (unreactive in the absence of acid) to [Ph_2_NH_2_]OTf (20 equiv, added via a syringe) caused distinct color changes that could be monitored by UV–vis spectroscopy (Figure 3). While higher energy absorptions (λ < 600 nm) are complicated by the absorption spectrum of (C_6_H_6_)_2_Cr^+/0^, the lower energy transitions provide a useful handle for the iron speciation. Upon addition of the acid (all 20 equiv) at RT, the characteristic near-IR absorption of [FeCN] (λ_max_ = 905 nm, FWHM ≈100 nm; pink trace) decayed rapidly (τ_1/2_ < 6 s) and a new, broader absorbance characteristic of [FeCNH_2_]^+^ (λ_max_ = 929 nm, FWHM ≈250 nm; blue trace) was observed.^25^ This feature decayed more slowly (τ_1/2_ ∼ 40 s under the conditions studied) with simultaneous growth of a shoulder that extends further into the near-IR, and a strong absorption centered at 485 nm (orange trace). These latter features are consistent with the formation of [FeOTf], as was also confirmed by ^1^H NMR spectroscopy (Figure S12). Products were also analyzed (1.0 equiv of NH_3_/Fe and 0.9 equiv CH_4_/Fe). These data demonstrate the aminocarbyne [FeCNH_2_]^+^ as an observable on-path intermediate in the conversion of [FeCN] to [FeOTf]. Isosbestic points at 520 and 900 nm establish that no further downstream intermediates buildup as [FeCNH_2_]^+^ is converted to [FeOTf] during liberation of NH_3_ and CH_4_.

**Figure 4:**
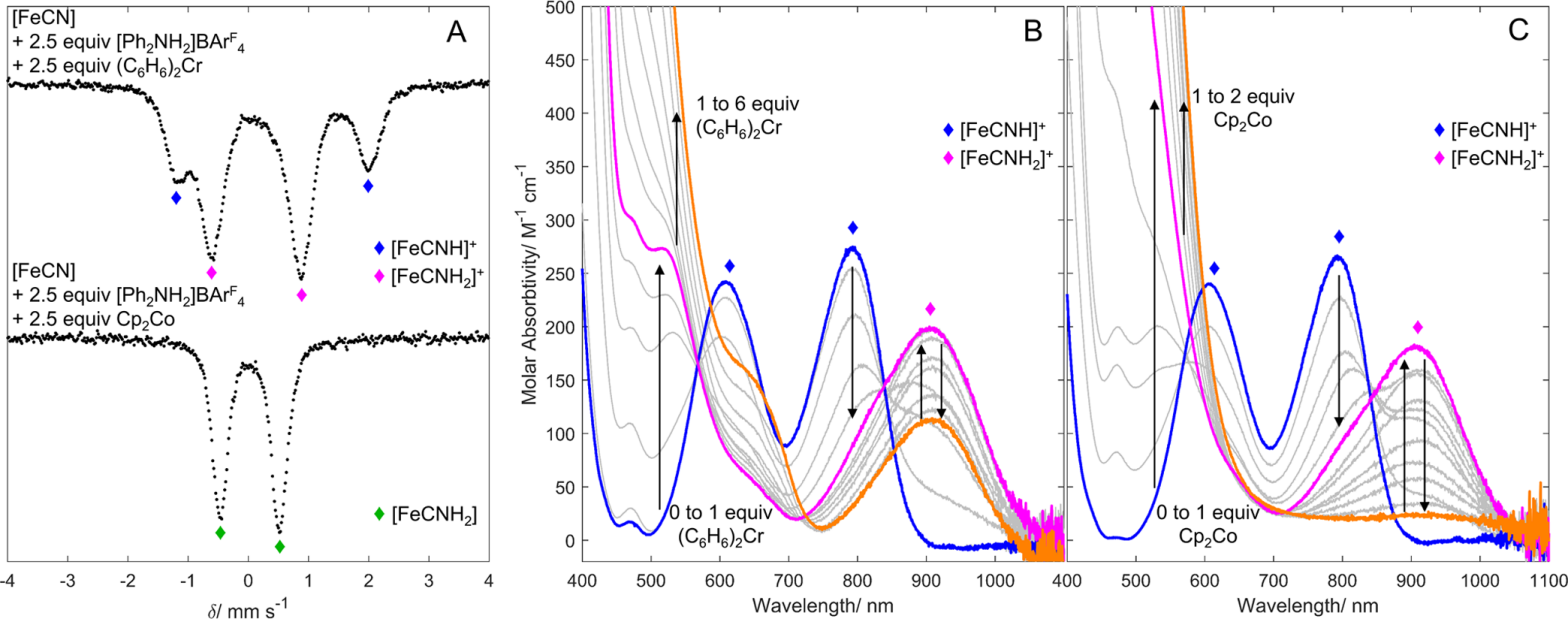
(A) ^57^Fe Mössbauer spectra of reaction of [FeCN] with [Ph_2_NH_2_]BAr^F^_4_ and (C_6_H_6_)_2_Cr or Cp_2_Co. (B, C) UV–vis data for reaction of [FeCN] with [Ph_2_NH_2_]BAr^F^_4_ and (C_6_H_6_)_2_Cr (B) or Cp_2_Co (C) to form early intermediates [FeCNH]BAr^F^_4_ (blue trace), [FeCNH_2_]BAr^F^_4_ (pink trace), and [FeCNH_2_]^0^ (orange trace).

To interrogate shorter-lived intermediates, we next studied the consumption of [FeCN] at lower temperature, using fewer equivalents of reductant and acid. Mixing [^57^FeCN] with [Ph_2_NH_2_]BAr^F^_4_ and (C_6_H_6_)_2_Cr (2.5 equiv each) in Et_2_O at −78 °C for 1 min, followed by freeze-quench (77 K) and analysis by Mössbauer spectroscopy, showed primarily [^57^FeCNH_2_]BAr^F^_4_ (Figure 4a; δ = 0.13 mm s^–1^ and Δ*E*_Q_ = 1.47 mm s^–1^), again supporting the proposed intermediacy of [FeCNH_2_]^+^ during catalysis.^25^ These low-temperature conditions also allowed identification of the first intermediate of protonation, [^57^FeCNH]BAr^F^_4_ (δ = 0.407 mm s^–1^ and Δ*E*_Q_ = 3.20 mm s^–1^),^25^ as a minor component, consistent with step (a) (Scheme 1).

We also obtained evidence for a facile redox equilibrium between [FeCNH_2_]^+/0^ and (C_6_H_6_)_2_Cr^+/0^, as can be expected based on the estimated difference in their reduction potentials (*E*°(Fe^+/0^) ∼ −1.2 V, *E*°(Cr^+/0^) = −1.2 V; see Section S8.3 for data). Relatedly, the available data imply that single electron reduction of [FeCNH_2_]^+^ to [FeCNH_2_] (step d) is feasible under conditions relevant to the catalysis. Accordingly, addition of 2.5 equiv of [Ph_2_NH_2_]BAr^F^_4_ to a THF solution of [FeCN] at −80 °C resulted in the immediate formation of [FeCNH]BAr^F^_4_ (Figure 4b, blue trace).^25^^,^^47^ Following this, the solution was titrated with 0–6 equiv of (C_6_H_6_)_2_Cr to study its response (Figure 4b). During the addition of the first equivalent of (C_6_H_6_)_2_Cr, UV–vis maxima for [FeCNH]^+^ (800 and 610 nm) decreased in intensity and new maxima appeared, reflecting the growth of [FeCNH_2_]^+^ (929 and 570 nm; pink trace). Isosbestic points at 570 and 860 nm establish no other intermediate buildup. Upon addition of further equivalents of (C_6_H_6_)_2_Cr, the signals for [FeCNH_2_]^+^ attenuate with corresponding growth of a strong absorbance with a shoulder at around 560 nm (orange trace). These changes are consistent with the reduction of [FeCNH_2_]^+^ to [FeCNH_2_]. Still, even after addition of 6 equiv (C_6_H_6_)_2_Cr, a large fraction of [FeCNH_2_]^+^ remained.^48^ These results confirm a redox equilibrium between [FeCNH_2_]^+/0^ and (C_6_H_6_)_2_Cr^+/0^ (step (d)).

As expected for such a redox equilibrium, cobaltocene, a stronger reductant than (C_6_H_6_)_2_Cr (*E*°(Cp_2_Co^3+/2+^) = −1.3 V), completely reduces [FeCNH_2_]^+^ to [FeCNH_2_]. Accordingly, ^57^Fe Mössbauer spectra of the reaction between [^57^FeCN] with [Ph_2_NH_2_]BAr^F^_4_ and Cp_2_Co (2.5 equiv each) at −78 °C in Et_2_O reveal the formation of a single new species (δ = 0.02 mm s^–1^ and Δ*E*_Q_= 0.99 mm s^–1^; Figure 4a). These parameters closely resemble those of [FeCNMe_2_] (δ = 0.06 mm s^–1^ and Δ*E*_Q_= 1.12 mm s^–1^),^25^ consistent with formation of [FeCNH_2_]. Complete formation of [FeCNH_2_] with only 2.5 equiv of reductant differs markedly from conditions using (C_6_H_6_)_2_Cr. Titrations monitored by UV–vis spectroscopy using Cp_2_Co still showed [FeCNH_2_]^+^ as an intermediate upon addition of just 1 equiv of Cp_2_Co to a mixture of [FeCN] and [Ph_2_NH_2_]BAr^F^_4_ (Figure 4c, pink trace). Upon addition of a second equiv of Cp_2_Co, [FeCNH_2_]^+^ is fully consumed with concomitant formation of [FeCNH_2_] (orange trace).

### Evidence for C–H Bond Formation via Fe=C(H)NH_2_^+^

The intermediacy of iron carbynes [FeCNH_2_]^+/0^ in catalytic CN^–^ reduction corresponds to the intermediacy of isolobal hydrazidos [FeNNH_2_]^+/0/–^, during Fe-catalyzed N_2_R.^27−29,49,50^ With this analogy in mind, we wondered whether iron carbynes might be selectivity determining in CN^–^ reduction, with final N–H bond formation releasing NH_3_ (analogous to N_β_–H bond formation in N_2_R via hydrazido intermediates), resulting in the observed 6 e^–^ products (CH_4_ and NH_3_), possibly via a transient carbide [Fe(C)] intermediate. Computational evidence and a study of the reactivity of the methylated carbyne [FeCNMe_2_] complex instead support the formation of a C–H bond via a carbene intermediate, [Fe=C(H)(NH_2_)]^+^, as the next step of the cycle. This path implies that the aminocarbyne is not selectivity determining in the present system; C–N bond cleavage occurs later in the catalytic cycle.

**Scheme 2:**
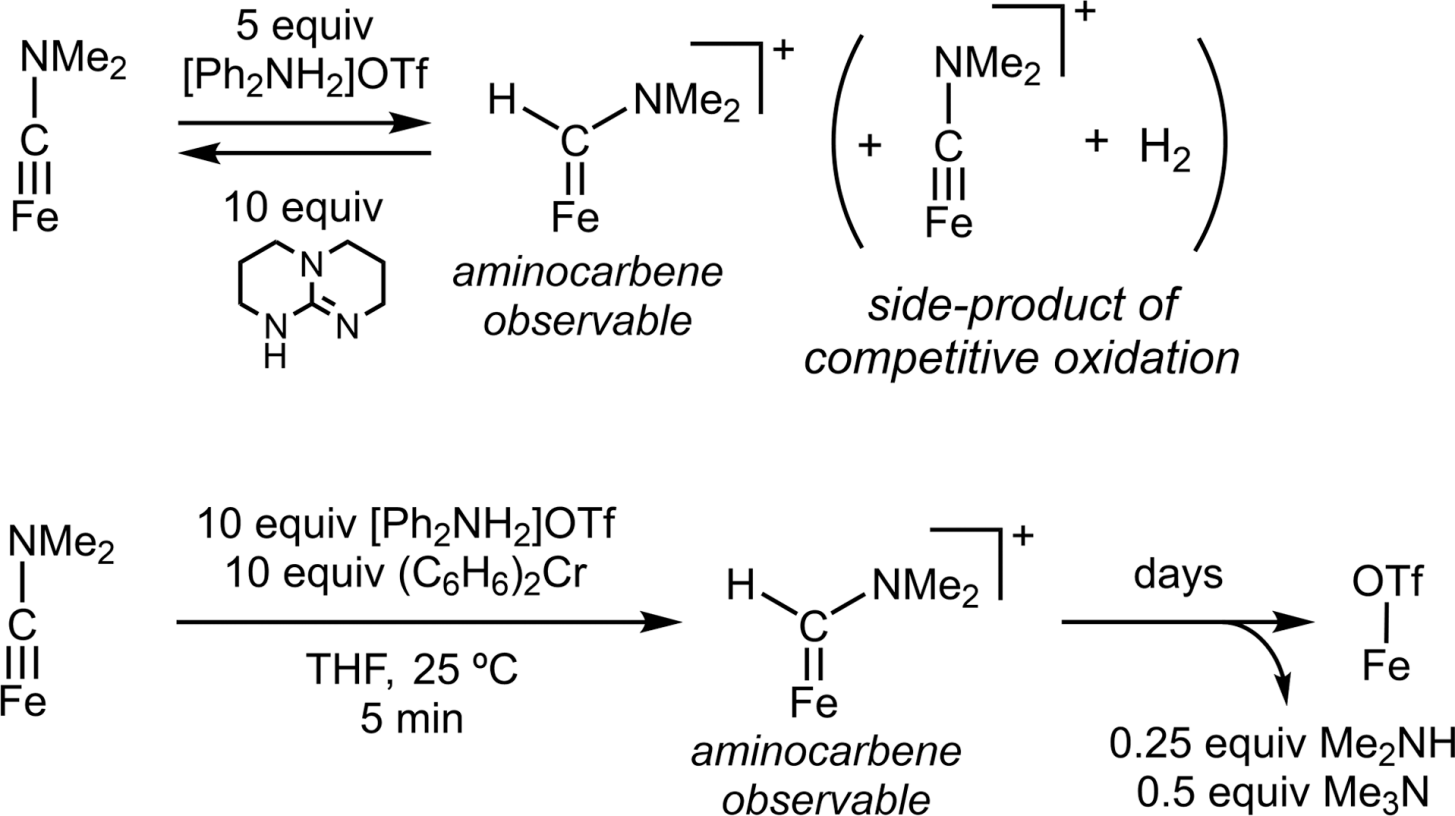
Protonation and Proton-Coupled Reduction of [FeCNMe_2_] as a Model of [FeCNH_2_] Reactivity

As intermediates downstream of [FeCNH_2_] cannot be identified during the CN^–^ reduction process, we studied the reactivity of the more tractable, methylated [FeCNMe_2_] analogue (Scheme 2). Thus, a reaction between [FeCNMe_2_] and [Ph_2_NH_2_]OTf in the absence of added reductant affords a new paramagnetic species, observed via UV–vis and ^1^H NMR spectroscopy (see SI, Section S9); some competing oxidation to [FeCNMe_2_]^+^ (with loss of H_2_) is also observed in the reaction mixture, frustrating isolation and purification of the new species. Nonetheless, on the basis of reactivity and ^57^Fe Mössbauer (*vide infra*), we assign the product of protonation as the aminocarbene [FeC(H)(NMe_2_)]^+^. Its formation is reversible; [FeCNMe_2_] is cleanly regenerated upon addition of a triazabicyclodecene (TBD) base (Scheme 2 and Figure S32).

[FeCNMe_2_] also reacts with 10 equiv [Ph_2_NH_2_]OTf in the presence of 10 equiv (C_6_H_6_)_2_Cr in THF at room temperature and is gradually converted to [FeOTf] over a period of 7 days, with Me_2_NH (0.25 equiv) and (curiously) Me_3_N (0.5 equiv) detected as the N-containing products.^51^ Notably, we had previously observed that [FeCNMe_2_] is not reduced in combination with Cp*_2_Co and [^2,5-Cl^PhNH_3_]OTf.^25^

While formation of [FeOTf] from [FeCNMe_2_] is slow in the presence of [Ph_2_NH_2_]OTf and (C_6_H_6_)_2_Cr (at 10 equiv each), the [FeCNMe_2_] is consumed rapidly (τ_1/2_ ≈ 1 min at 25 °C) and the same paramagnetic (presumed) carbene species is now also observed as an intermediate. Hence, reacting [FeCNMe_2_] with excess (C_6_H_6_)_2_Cr and [Ph_2_NH_2_]OTf and freeze-quenching the reaction after 5 min revealed a new major species with the Mössbauer parameters δ = 0.40 mm s^–1^ and Δ*E*_Q_ = 2.25 mm s^–1^ (Figure S37), consistent with an *S* = 1 [FeC(H)(NMe_2_)]^+^ species with parameters similar to previously characterized *S* = 1 (P_3_^Si^)Fe^II^–L^+^ species (L = CO, CNR or N_2_).^36,52^ Taken together, these data are highly consistent with [FeC(H)(NMe_2_)]^+^ as an intermediate during the reductive protonation of [FeCNMe_2_] to liberate [FeOTf] and the amine products and suggest that [FeC(H)(NH_2_)]^+^ would form readily via protonation of [FeCNH_2_] during CN^–^ reduction catalysis.

**Figure 5:**
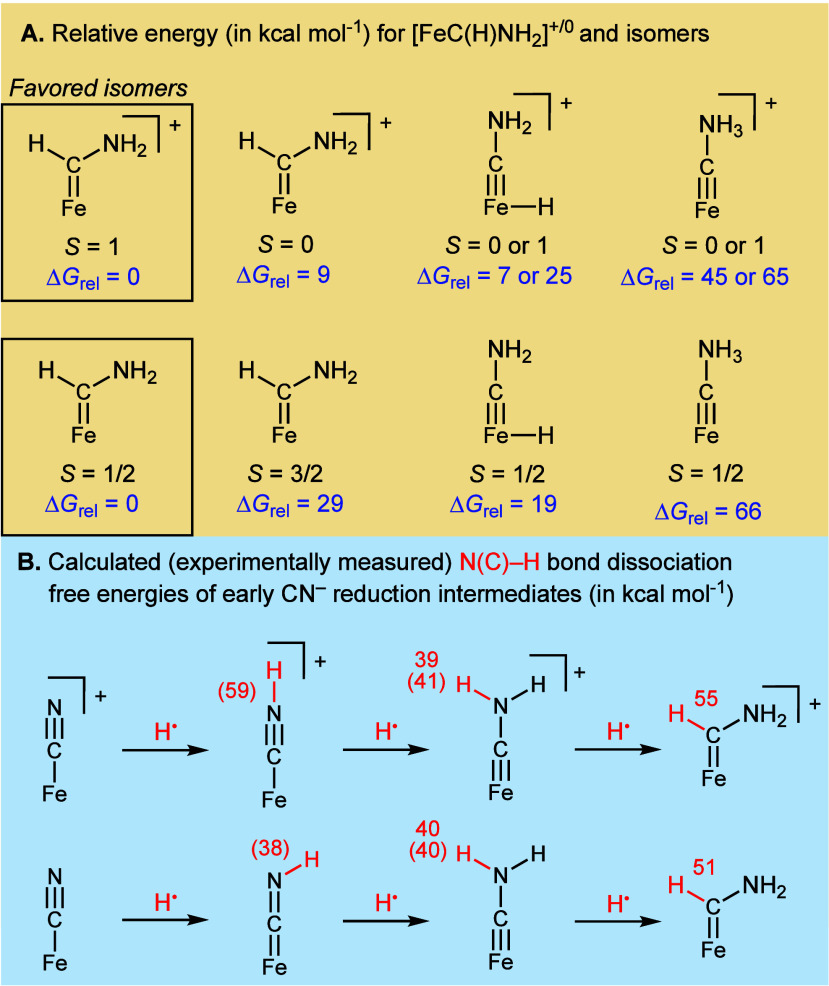
(A) Comparing the energies of isomers in specified spin states for [FeC(H)(NH_2_)]^+/0^. (B) Calculated N–H bond and C–H bond dissociation free energies (BDFEs). Experimentally determined values are provided in parentheses; for carbynes, these are estimated from [FeCN(H)(Me)]^+/0^.^36^

To gain further support for this proposed [Fe=C(H)(NH_2_)]^+^ intermediate, we turned to computational methods to explore the energy of aminocarbene species versus other plausible isomers. The TPSS functional^53^ and a def2-TZVP basis set on Fe, with a def2-SVP basis set on all other atoms,^54^ reliably replicate experimentally estimated BDFEs for complexes similar to those discussed here.^55^ We thus used this approach to compare plausible isomers with specified spin states under the addition of H^•^ to the [FeCNH_2_]^+/0^ carbynes (Figure 5a). We find that iron carbenes [Fe=C(H)(NH_2_)]^+/0^ in their corresponding *S* = 1 and *S* = 1/2 spin states, respectively, are the lowest energy isomers (Δ*G*_rel_ = 0 kcal mol^–1^) when compared to their corresponding ammonium carbyne isomers ([Fe≡C–NH_3_]^+/0^; Δ*G*_rel_ = 45–66 kcal mol^–1^) and iron carbyne hydrides ([(H)Fe≡C–NH_2_]^+/0^; Δ*G*_rel_ = 7–25 kcal mol^–1^). Alternative spin states of the iron carbenes (S = 0 or 3/2) are also higher in energy (Δ*G*_rel_ = 9 and 29 kcal mol^–1^, respectively). The small Δ*G*_rel_ of [(H)Fe≡C–NH_2_]^+^ is interesting given that iron hydrides can be catalytic sinks for this system;^46^ isomerization between the on-path iron carbene and this iron carbyne hydride might be a relevant deactivation pathway.

The thermodynamic favorability of C–H bond formation (over N–H bond formation) can be rationalized by considering the basicity of the N and C atoms of the iron aminocarbyne. [FeCNH_2_] features a planar sp^2^-hybridized N atom, suggesting substantial π donation from N, which can be expected to make the N atom less basic than the carbyne C atom. Such a scenario would favor C atom protonation as observed.

**Figure 6:**
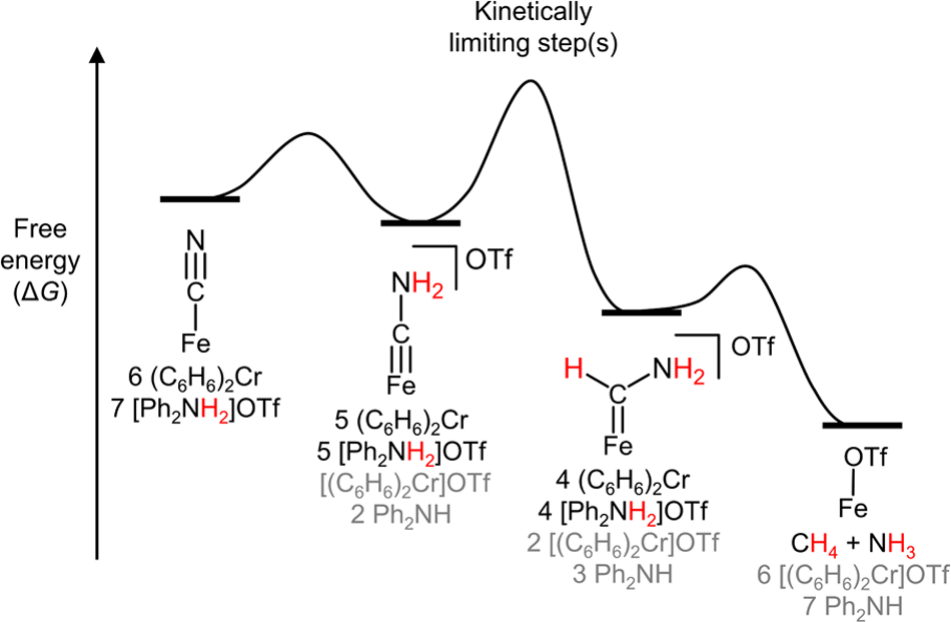
Proposed qualitative energy barriers for transformation of [FeCN] to [FeOTf] with key intermediates, [FeCNH_2_]^+^ and [FeC(H)(NH_2_)]^+^ indicated.

Computationally, the carbene [FeC(H)(NH_2_)]^+/0^ C–H bonds (51–55 kcal mol^–1^) are much stronger than the carbyne [FeCNH_2_]^+/0^ N–H bonds (39–40 kcal mol^–1^; Figure 5b), consistent with the conversion of carbyne to carbene being a thermodynamically favorable step in [FeCN] reduction (Figure 6). However, the buildup of [FeCNH_2_]^+^ as an observable intermediate when [FeCN] is reduced to NH_3_ and CH_4_ (Figure 3), and the slow protonation observed for [FeCNMe_2_]^0^ (Scheme 2) suggests a significant kinetic barrier in converting [FeCNH_2_]^+/0^ to [FeC(H)(NH_2_)]^+/0^ (Figure 6). This can be rationalized by a rehybridization at carbon with a corresponding change in spin state upon protonation, which would correlate with a significant kinetic barrier.

## Discussion

### Comparison to Other Fe-Based Catalysts

As introduced above, reported Fe catalysts for cyanide (or HCN) reduction have exclusively been Fe–S clusters, as either the protein active sites of nitrogenase enzymes,^5−9,13^ the extracted cofactors (e.g., FeMoco),^10−12^ or synthetic clusters.^20−22^ The extracted cofactors and synthetic clusters studied have been shown to reduce CN^–^ using weak acids (lutidinium or pH 8 buffered solutions) and lanthanide(II) reductants (SmI_2_ or Eu^II^(DTPA)) as a source of H^+^/e^–^ equivalents. Invariably, these systems have produced substantial amounts of C_2+_ products, accounting for 20–40% of the total reduced carbon products, in addition to C_1_ products, including CH_4_ (and NH_3_) or CH_3_NH_2_.^10−12,20−22^

By contrast, the [FeCN] catalyst studied herein shows <2% C_2+_ products. Curiously, its reactivity profile more closely resembles that of HCN reduction by MoFe nitrogenase, where C_2+_ products account for <0.1% of the total reduced carbon.^6,7^ Still, [FeCN] shows much higher selectivity for the 6 e^–^ reduction products (CH_4_ + NH_3_) than has been observed for the nitrogenases studied (MoFe and VFe variants), which also show significant CH_3_NH_2_ production (MoFe, CH_3_NH_2_:CH_4_ = 0.39; VFe, CH_3_NH_2_:CH_4_ = 0.66–1.1).^9^ The complex (PhBP^iPr^_3_)FeBr, while a less active catalyst system for CN^–^ reduction (Table 1, entry 10), more faithfully captures the selectivity of nitrogenases, producing substantial CH_3_NH_2_ as well as (CH_4_ + NH_3_). As functional models, the (P_3_^B^)Fe– and (P_3_^Si^)Fe–systems we have studied are distinct in that both have been shown to display catalytic activity for N_2_R and CN^–^ reduction, akin to ATP-dependent nitrogenase enzymes.^1,5,16,43^

### Mechanistic Findings

The data presented above allow us to posit several important intermediates we believe to be on the path for catalytic CN^–^ reduction by [FeCN] (Scheme 1) and to further consider the observed selectivity. A key observation from low-temperature UV–vis titrations includes the finding that [FeCN] is readily protonated by [Ph_2_NH_2_]OTf. The resulting isocyanide [FeCNH]^+^ can be reduced by (C_6_H_6_)_2_Cr, and the resulting [FeCNH]^0^ product is rapidly protonated to afford the observable aminocarbyne [FeCNH_2_]^+^. With (C_6_H_6_)_2_Cr present as the reductant, [FeCNH_2_]^+^ and [FeCNH_2_] were shown to be in redox equilibrium.

While we observe downstream conversion of [FeCNH_2_]^+^ to [FeOTf] at room temperature (associated with liberation of NH_3_ and CH_4_), we have been unable to characterize intermediates of this transformation even at low temperature. However, by reconciling computational data with the observed reactivity of a methylated analogue, [FeCNMe_2_], we favor a C–H bond forming step to produce [FeC(H)(NH_2_)]^+^ as the next intermediate from [FeCNH_2_]^+^ along the catalytic pathway, possibly via ET-PT (steps d and e in Scheme 1) or PCET (step f). This Fischer-type aminocarbene would plausibly be on the path for either CH_4_ + NH_3_ (6 e^–^), or CH_3_NH_2_ (4 e^–^), products. The selectivity determining C–N bond cleaving step that produces the 6 e^–^ products in this system must therefore occur at a later stage of the catalytic cycle, with additional (and facile) 4H^+^/4e^–^ transfers (Scheme 1, step g).

**Figure 7:**
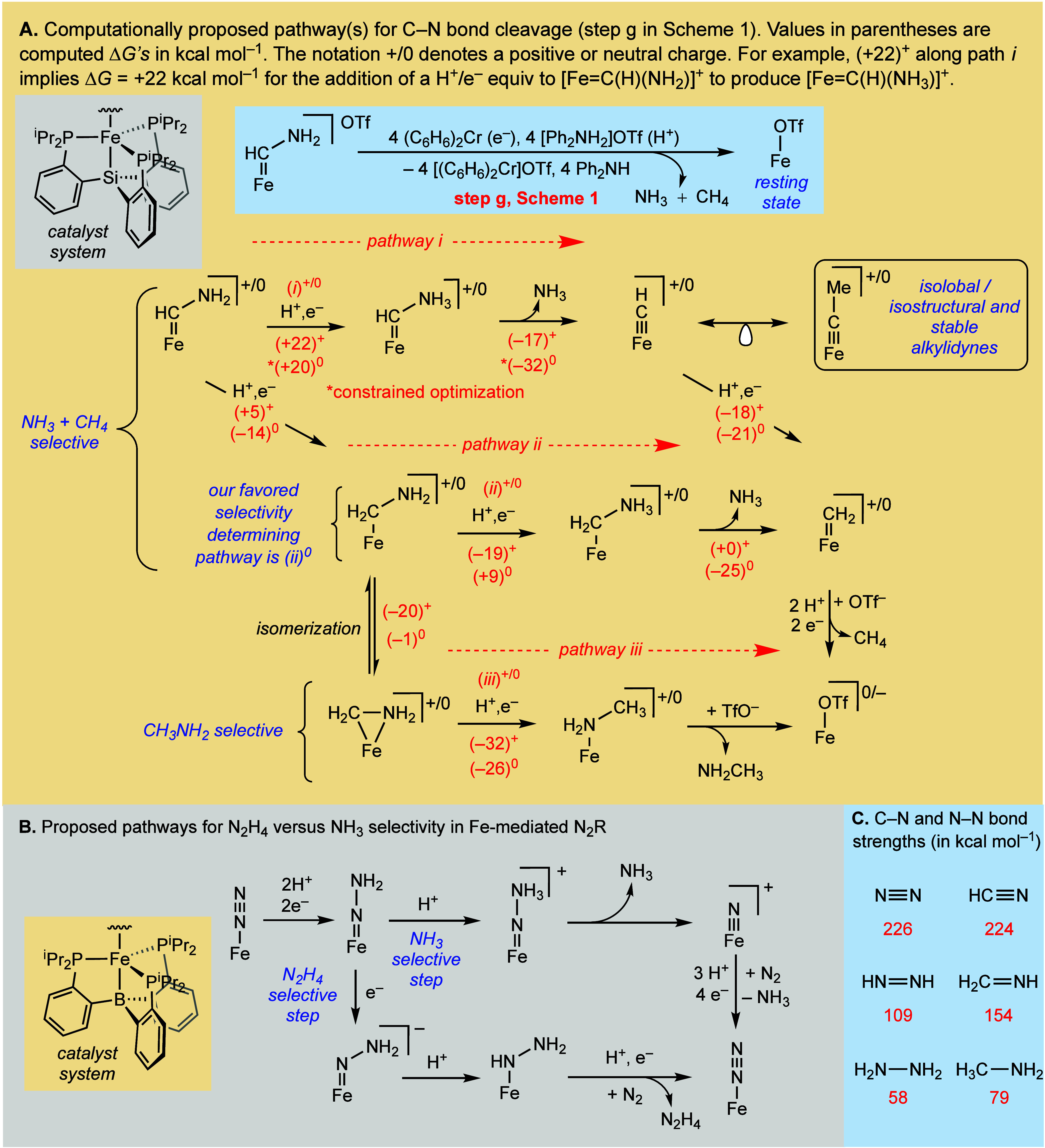
(A) Plausible pathways *i*–*iii* for C–N bond cleavage via step g in Scheme 1 for CN^–^ reduction by the P_3_^Si^Fe system, attempting to rationalize selectivity, with associated thermodynamic data for stepwise e^–^/H^+^ transfers en route to product, with values for both cationic (+) and neutral (0) species. Pathway *ii*^0^ is our favored pathway. (B) Comparison with N–N bond cleavage in Fe-mediated N_2_R by the P_3_^B^Fe-system, accounting for NH_3_ versus N_2_H_4_ selectivity. (C) Comparison of N–N and C–N bond strengths in N_2_ and HCN and their further reduced derivatives.^61^

From the Fischer carbene (or its one-electron reduced congener), several pathways can account for CH_4_ and NH_3_ products (depicted in Figure 7a). Guided by theoretical studies, we can, qualitatively at least, compare them. Ultimately, each specific H^+^ and e^–^ step likely needs to be examined to fully account for CH_4_ and NH_3_/CH_3_NH_2_ selectivity, as has been the case for NH_3_/N_2_H_4_ selectivity during N_2_R (Figure 7b).^28,29,50^ However, acknowledging increased error in theoretical calculations when studying changes in charge state,^56^ we have opted to limit our present considerations to the thermodynamics of the addition of a net H atom to [FeC(H)_*x*_(NH_2_)]^+/0^ (*x* = 1, 2) species, and the associated C–N bond strengths of the ammonium intermediates, [FeC(H)_*x*_(NH_3_)]^+/0^, that form. The combined H^+^/e^–^ transfers (Figure 7a) are referenced to the combination of (C_6_H_6_)_2_Cr (e^–^) and [Ph_2_NH_2_]OTf (H^+^; see the SI for details).

We consider three pathways in Figure 7a(i–iii) as an expansion on step g introduced in Scheme 1. Pathways i–iii proceed via either cationic or neutral intermediates, and we use +/0 to differentiate between these charge states in the figure.

Starting from the aminocarbene +/0 intermediates, addition of the next H^+^/e^–^ equivalent at N would yield an ammonium carbene, [FeC(H)(NH_3_)]^+/0^, which could liberate NH_3_ and an iron methylidyne, [Fe≡C–H]^+/0^. The methylidyne is envisioned to be reductively protonated to form CH_4_ and [FeOTf] (Figure 7a, pathway i). The plausibility of iron methylidyne intermediacy in this P_3_^Si^Fe catalyst system is supported by our recent report of the isolation and structural characterization of the methylated analogues [P_3_^Si^Fe≡C–CH_3_]^+/0^.^44^

Alternatively, C–H instead of N–H bond formation from [FeC(H)(NH_2_)]^+/0^ would yield an iron alkylamine product. We consider computationally such a species as two spin-isomers, a low spin (*S* = 0, 1/2) η^2^-iminium adduct ([Fe(η^2^–CH_2_NH_2_)]^+/0^) and an intermediate spin (*S* = 1, 3/2) η^1^–alkylamine ([Fe(η^1^-CH_2_NH_2_)]^+/0^). In [Fe(η^1^-CH_2_NH_2_)]^+/0^ intermediates, N–H bond formation is likely kinetically favorable (see below), but from the η^2^-iminium adduct, we anticipate similar barriers for C–H and N–H bond formation. From either alkylamine isomer, N–H bond formation would yield the alkylammonium product [FeC(H)_2_(NH_3_)]^+/0^ (pathway *ii*). C–N bond cleavage releases NH_3_ and an iron methylidene ([Fe=CH_2_]^+/0^). While we have not previously characterized a terminal P_3_^Si^Fe=CR_2_ carbene (for R = H or alkyl), cationic, diamagnetic iron methylidenes, [CpFe(L_2_)=CH_2_]^+^ (L = phosphine or CO), have been synthesized by O atom protonation of a corresponding methoxymethyl iron complex, followed by C–O bond cleavage.^57−59^ Such a scenario is akin to the C–N bond cleavage suggested here. Addition of a further 2H^+^/2e^–^ releases CH_4_ from [FeCH_2_]^+/0^.

A CH_3_NH_2_ selective pathway, (*iii*)^+/0^, has also been considered, where the addition of H^+^/e^–^ to the C atom of [Fe(η^2^-CH_2_NH_2_)]^+/0^ results in a methylamine adduct, [FeNH_2_CH_3_]^+/0^. The latter should readily liberate CH_3_NH_2_ upon reduction, as has been demonstrated for the ammonia-complex, [P_3_^Si^FeNH_3_]^+^.^60^

Computational analysis of the intermediates along these three pathways shows that the C–H bond formation is always thermodynamically favored. Consequently, if the strongest bond is always formed, CN^–^ reduction would produce CH_3_NH_2_ instead of CH_4_ and NH_3_ (Figure 7; pathway *iii*). Hence, to account for the observed CH_4_ and NH_3_ products, we propose that the bulky ^*i*^Pr-groups on the P_3_^Si^ ligand limit access to the carbyne C atom and thereby kinetically favor N–H bond formation. This leads to ammonium intermediates [FeC(H)_*x*_(NH_3_)]^+/0^ (*x* = 1,2) that ultimately liberate NH_3_ (and then CH_4_; Figure 7, pathways *i* and *ii*). This rationalization accommodates the observed mixture of CH_3_NH_2_ and NH_3_ observed when using (PhBP^iPr^_3_)FeBr as a catalyst instead of [Fe] (Table 1, entry 10); the 4-coordinate Fe center in (PhBP^iPr^_3_)FeBr affords a more accessible carbyne intermediate C atom.^62^ Accordingly, the rate of C–H bond formation can compete with N–H bond formation and CH_3_NH_2_ is an observable product. Relatedly, we suspect that the increased steric bulk at N in [FeCNMe_2_] slows the rate of N–H bond formation, leading to the observed product distribution (2:1 Me_3_N:Me_2_NH).

With these considerations, we next compared the NH_3_/CH_4_ selective pathways (*i* and *ii*). We favor pathway (*ii*)^0^ as a pathway involving exothermic and mildly endergonic steps, with C–N bond cleavage occurring from [FeCH_2_NH_3_]^0^. Considering pathways (*i*)^+/0^, they feature highly endergonic N–H bond formation steps (+22 and +20 kcal mol^–1^, respectively). As this step is followed by exothermic C–N bond cleavage and reductive protonation of the resulting methylidyne, pathways (*i*)^+/0^ may nevertheless be kinetically competent.^63^ Path (*ii*)^+^ features downhill or mildly endergonic steps. We nevertheless disfavor this pathway (compared to (*ii*)^0^) due to the high favorability of the diamagnetic [Fe(η^2^-CH_2_NH_2_)]^+^ (−20 kcal mol^–1^ compared to [Fe(η^1^-CH_2_NH_2_)]^+^), with subsequent H^•^ addition at C rather than N being very exothermic from [Fe(η^2^-CH_2_NH_2_)]^+^.

Our favored path (*ii*)^0^ points to a selectivity determining step at the addition of a (net) H^•^ to [Fe(η^1^-CH_2_NH_2_)]^0^; the latter constitutes a 4H^+^/4e^–^ intermediate of [FeCN] reduction. By contrast, the proposed selectivity determining intermediate during N_2_R is [FeNNH_2_], a 2H^+^/2e^–^ intermediate (Figure 7b). Compared to isolobal N≡N, C≡N^–^ requires a greater degree of reduction before the C–N bond cleavage can occur. This is consistent with the respective C–N and N–N σ bond strengths: While their triple bond strengths are similar, the bond weakening upon decrease in bond order is much greater for N_2_ (Figure 7c).^61^

In addition to the high selectivity for CH_4_ and NH_3_, CN^–^ reduction of [FeCN] has high C_1_ selectivity compared with other Fe catalysts. When considering the origin of this selectivity, it is worth noting that the addition of multiple cyanide ligands bound to Fe has not been observed during catalysis or chemical experiments. This might be critical for the high selectivity for the C_1_ products. The precedent for C–C coupling of CO or CNR ligands at mononuclear metal sites requires two of these ligands bound to the metal prior to coupling.^64−67^ The sterically encumbered, four-coordinate P_3_^Si^ ligand hinders the facile addition of multiple equivalents of CN^–^, maintaining the trigonal bipyramidal geometry during catalysis.^68^ We propose that this results in high yields for C_1_ products. Accordingly, the more flexible (P_3_^B^)Fe platform^14,55^ and the less encumbered (PhBP^iPr^_3_)Fe platform^62^ both have high C_2_/C_1_ ratios (0.16 and 0.11, respectively) compared to [FeCN] (0.02). Relatedly, a previously synthesized and stable compound, (PhBP^iPr^_3_)Fe(CNR)_2_, demonstrates that (PhBP^iPr^_3_)Fe could accommodate two CN^–^ ligands (or further protonated derivatives) in a 5-coordinate structure, likely needed for C_2_ product formation.^69^

Finally, it is interesting to compare the strength of the reductant used herein for the CN^–^ reduction ((C_6_H_6_)_2_Cr; *E* °= −1.2 V), with a common reductant used for N_2_R via related iron catalysts Cp*_2_Co (*E*° = −1.9 V). Proposed pathways for catalysis require a turnover limiting potential (*E*° ≈ −2.0 V) that generates an FeN_2_^–^ species before protonation (to generate FeN_2_H) can occur, necessitating reductants as strong as Cp*_2_Co.^38,40,70^

By contrast, the basicity of the CN^–^ ligand enables protonation of [FeCN] prior to an ET step.^25,36^ Consequently, the turnover limiting potential is that of [FeCNH]^+/0^ (*E*° = −1.3 V), not [FeCN]^0/–^ (*E*°= −2.1 V), allowing the use of a comparatively mild reductant like (C_6_H_6_)_2_Cr. If initial protonation, or PCET, occurs before any independent ET steps, the turnover limiting potential for catalysis can be significantly less reducing. Indeed, Schrock’s original triamidoamine Mo–N_2_ catalyst system is thought to proceed via an initial PT step, and it is compatible with correspondingly milder reductants (e.g., Cp*_2_Cr) for turnover.^15,71,72^

## Conclusions

In conclusion, we have described the catalytic reductive protonation of CN^–^ to primarily NH_3_ and CH_4_, by a mononuclear Fe complex, with selectivities comparable to those observed for CN^–^ reduction by nitrogenase. We also report mechanistic studies that show terminal iron aminocarbyne (FeCNH_2_) intermediates, which are structurally similar to iron hydrazido intermediates (FeNNH_2_) of Fe-mediated N_2_R, as on-path in the CN^–^ reduction cycle. Experimental and computational studies suggest that these aminocarbynes undergo further C–H bond formation(s) prior to C–N bond cleavage, resulting in the selectivity observed, in contrast to iron hydrazidos during N_2_R. Via this study, a terminal transition metal carbyne is hence invoked as a critical intermediate in the catalytic reductive protonation of a robust small molecule (CN^–^).

## Supporting Information Available

Computational models (TXT)
Experimental methods; ammonia production and quantification studies; detection and quantification of gaseous products; additional control experiments; additional NMR experiments; ^57^Fe Mössbauer spectroscopy; UV–vis spectroscopy; electrochemical methods; generation of proposed [FeC(H)NMe_2_]^+^; derivation of estimated BDFE (bond dissociation free energy) for early N–H bonds; and computational methods (PDF)

## Abbreviations

[TBA]: tetrabutylammonium
OTf: triflate
BAr^F^_4_: tetrakis(3,5-bis(trifluoromethyl)phenyl)borate
Cp: cyclopentadienyl
Cp*: pentamethylcyclopentadienyl
(12-c-4): 12-crown-4 (1,4,7,10-tetraoxacyclododecane)
