# Supplementary material for: Catalytic Reduction of Cyanide to Ammonia and Methane at a Mononuclear Fe Site
Source: J Am Chem Soc. 2024 Feb 16;146(8):5343–54. doi: 10.1021/jacs.3c12395 (PMC10910527; doi:10.1021/jacs.3c12395)
Supplement: Supplementary file 2 — ja3c12395_si_002.pdf [file ja3c12395_si_002.pdf]

Supporting information for

Catalytic reduction of cyanide to ammonia and methane at a mononuclear Fe-site

Christian M. Johansen and Jonas C. Peters*

Correspondence to: jpeters@caltech.edu

Table of Contents:

**2**	S1. Experimental section
**3-9**	S2. Ammonia production and quantification studies
**10-14**	S3. Detection and quantification of gaseous products
**15-16**	S4. Additional control experiments
**17-22**	S5. Additional NMR experiments
**23-27**	S6. ^{57}Fe Mössbauer spectroscopy
**28-29**	S7. UV-vis spectroscopy
**30-36**	S8. Electrochemical methods
**37-41**	S9. Generation of proposed $[FeC(H)NMe_2]^+$
**42-43**	S10. Derivation of estimated BDFE (Bond Dissociation Free Energy) for early N−H bonds
**44-50**	S11. Computational methods
**51-52**	S12. References

S1. Experimental section

S1.1 General Considerations:

All manipulations were carried out using standard Schlenk or glovebox techniques under an N_2 atmosphere. Solvents were deoxygenated and dried by thoroughly sparging with N_2 followed by passage through an activated alumina column in a solvent purification system by SG Water, USA LLC. Nonhalogenated solvents were tested with sodium benzophenone ketyl in tetrahydrofuran (THF) to confirm the absence of oxygen and water. Deuterated solvents were purchased from Cambridge Isotope Laboratories, Inc., degassed, and dried over activated 3-Å molecular sieves prior to use. [FeCN],[1] [FeCNMe$_2$],[1] [$P_3{}^BFe$]BAr$^F{}_4$,[2] $^{Ph}B(^{iPr2}P)_3FeBr$,[3] [Ph_2NH_2]OTf,[4] KC_8[1], [$H(OEt_2)_2$]BAr$^F{}_4$[5], [TBA][^{13}CN][6] were synthesized using literature methods. $(C_6H_6)_2Cr$, $Cp^*{}_2Cr$ and Cp_2Co were purchased from Strem and used without further purification.

S1.2 Physical Methods

NMR: Nuclear Magnetic Resonance (NMR) measurements were recorded with a Varian 400 MHz spectrometer. ^1H NMR chemical shifts are reported in ppm relative to tetramethylsilane, using ^1H resonances from residual solvent as internal standards.[7]

UV-Vis: Ultraviolet-visible (UV-vis) absorption spectroscopy measurements were collected with a Cary 50 UV-vis spectrophotometer using a 1 cm path length quartz cuvette. All samples had a blank sample background subtraction applied. Temperature regulation for UV-Vis measurements was carried out with a Unisoku cryostat. Time course UV-Vis spectra were collected with the Scanning Kinetics application of the Cary WinUV software.

Mössbauer spectra: ^{57}Fe Mössbauer spectroscopy measurements were recorded on a spectrometer from SEE Co. (Edina, MN) operating in the constant acceleration mode in a transmission geometry. The sample was kept in an SVT-400 cryostat from Janis (Wilmington, MA). The quoted isomer shifts are relative to the centroid of the spectrum of a metallic foil of α-Fe at room temperature (RT). Solution samples were transferred to a sample cup and freeze-quenched with liquid nitrogen inside of the glovebox and then immersed in liquid N_2 until mounted in the cryostat. Data analysis was performed using version 4 of the program WMOSS (wmoss.org) and quadrupole doublets were fit to Lorentzian lineshapes.

S2 Ammonia production and quantification studies

S2.1 Standard NH_3 Generation Reaction Procedure

All solvents are stirred with Na/K for ≥2 hours and filtered prior to use. In a nitrogen-filled glovebox, the precatalysts ([FeCN] or other Fe-complexes) are weighed in individual vials.* The precatalysts are then transferred quantitatively into a Schlenk tube using benzene. The benzene is then lyophilized to provide a fluffy powder of precatalyst at the bottom of the Schlenk tube. The tube is then charged with a stir bar and the acid ([Ph_2NH_2]OTf), reductant (e.g., (C_6H_6)$_2$Cr)) and [TBA][CN], all added as solids. The tube is sealed and cooled to 77 K in a cold well. To the cold tube 1 mL Et_2O solvent is added to produce the desired precatalyst concentration. For reactions run above -20 °C additional Et_2O was added taking into account the headspace of the tube and the vapor pressure of Et_2O such that the reaction volume remained 1 mL. The temperature of the system is allowed to equilibrate for 5 minutes and then the tube is sealed with a Teflon screw-valve. This tube is passed out of the box into a liquid N_2 bath and transported to a fume hood. For experiments run at -78 °C the tube is then transferred to a dry ice/isopropanol bath where it thaws and is allowed to stir for minimum three hours before warming. For experiments run at -20 °C the tube is instead transferred to a 3:1 ice:NaCl bath where it thaws and is allowed to stir for minimum one hours before warming. For experiments run at 0 °C the tube is transferred to an ice bath where it thaws and is allowed to stir for minimum three hours before warming. For experiments run at 25 °C the tube is transferred to a water bath where it thaws and is allowed to stir for a minimum of 80 minutes. To ensure reproducibility, all experiments were conducted using 10 mm egg-shaped-stir bars and stirring was conducted at ~600 rpm.

* In cases where less than 2.3 µmol of precatalyst were used, stock solutions were used to avoid having to weigh very small amounts.

S2.2 NH_3 detection by ^{1}H NMR

Following the completed reaction, the reaction flask was cooled to 77 K and allowed to freeze. The reaction vessel is then opened to atmosphere and to the frozen solution is slowly added an excess KO^{t}Bu as a solid, (in at least two-fold excess with respect to acid) and THF (2 mL). This solution is allowed to freeze, then the headspace of the tube is evacuated, and the tube is sealed. The tube is then allowed to warm to RT and stirred at RT for at least 10 minutes. An additional Schlenk tube is charged with HCl (3 mL of a 2.0 M solution in Et_2O, 6 mmol) to serve as a collection flask. The volatiles of the reaction mixture are vacuum transferred into this collection flask. After completion of the vacuum transfer, the collection flask is sealed and warmed to RT. Solvent is removed *in vacuo*, and the remaining residue is dissolved in a known amount of (1 mL or 0.5 mL) DMSO-d_6 containing 1,3,5-trimethoxybenzene as an internal standard. Integration of the ^{1}H NMR peak observed for NH_4^+ (δ 7.25 t, J = 52 Hz, 4H) is then integrated against the two peaks of 1,3,5-trimethoxybenzene to quantify the ammonium present. A typical spectrum is shown in **Figure S1**. $MeNH_3Cl$ (when detected) is characterized in a similar fashion with integration of diagnostic peaks ($MeNH_3$.Cl δ 7.78, br, 3H, δ 2.35, q, J = 8 Hz, 3H) against 1,3,5-trimethoxybenzene. A typical spectrum is shown in **Figure S2**.

S2.3 Reload experiments

For the reload experiments (Table 1 entry 16), catalysis is setup as detailed in S.2.1, except that following the sealing of the tube, it was allowed to thaw inside the glovebox, and stirred for 80 minutes at 25 °C. After 80 minutes the reaction mixture is filtered through a Celite pipette, with the flask, and filter cake washed with an additional 2x(1 mL) dry Et_2O. The extracted Et_2O soluble products are added to a fresh Schlenk flask, and the solvent is removed *in vacuo*.

The (fresh) tube is then charged with a stir bar, the acid ($[Ph_2NH_2]OTf$), reductant ($(C_6H_6)_2Cr$)) and [TBA][CN]. The tube is sealed and cooled to 77 K in a cold well. To the cold tube Et_2O is added, taking into account the headspace of the tube and the vapor pressure of Et_2O such that the reaction volume remained 1 mL at 25 °C. The temperature of the system is allowed to equilibrate for 5 minutes and then the tube is sealed with a Teflon screw-valve. This tube is passed out of the box into a liquid N_2 bath and transported to a fume hood. The reaction is warmed to room temperature, and allowed to stir for 12 hours. The reloaded tube is analyzed as detailed in section 2.2.

The first catalytic run is also analyzed for NH_3 (as detailed in section 2.2), with the solids remaining on the Celite pipette being added back to the flask.

S2.4 A note on error bars and the solubility of species during catalysis

Compared to similar N_2 reduction reactions somewhat greater error between individual runs is observed.[4,7] We attribute this to the relative heterogeneity in the reaction mixture. $[Ph_2NH_2]OTf$ is insoluble in Et_2O while [FeCN], $(C_6H_6)_2Cr$ and [TBA][CN] are all sparingly soluble. As a result, sufficient and consistent stirring was required to achieve reproducibility, but nonetheless some variation was observed.

The relatively low catalyst solubility also necessitated the lyophilization of the precatalyst, particularly for low temperature experiments. The increased surface area of lyophilized materials helps more rapidly solubilize the catalyst. This was found to be crucial to achieve efficient catalysis. Attempts to counteract these effects, e.g. by using THF for increased solubility, gave lower yields (Table S1 runs A3 and B3), possibly due to a greater rate of background hydrogen evolution.

S2.5 NH_3 detection results

Table S1. Catalytic yields for reduction of [TBA][CN] to yield NH_3. [a]Assuming 6 e^- reduction. [b]CH_3NH_2 products also taken into account as a 4 e^- product.

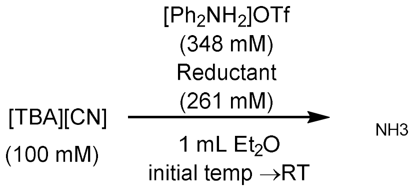

Entry	Catalyst loading (µmol) ([FeCN] is catalyst unless specified)	Conditions, (($C_6H_6)_2Cr$ is the reductant unless specified)	NH_3 (equiv/ Fe)	Yield per reductant (%)[a]
A1	0.72 µmol	25 °C, Et_2O	20.5	34.1
B1	0.72 µmol	25 °C, Et_2O	32.8	54.6
C1	0.72 µmol	25 °C, Et_2O	24.3	40.5
D1	0.72 µmol	25 °C, Et_2O	32.5	54.2
**Table 1, entry 1**	**0.72 µmol**	**25 °C, Et_2O**	**28±5**	**47±8**
E1	0.72 µmol	–20 °C, Et_2O	27.6	46
F1	0.72 µmol	–20 °C, Et_2O	43.4	72
**Table 1, entry 2**	**0.72 µmol**	**–20 °C, Et_2O**	**35±8**	**58±13**
G1	0.72 µmol	0 °C, Et_2O	27.6	46
H1	0.72 µmol	0 °C, Et_2O	24.3	40.5
**Table 1, entry 3**	**0.72 µmol**	**0 °C, Et_2O**	**26±2**	**43±3**
I1	0.72 µmol	–78 °C, Et_2O,	16.2	23
J1	0.72 µmol	–78 °C, Et_2O	39.0	55
K1	0.72 µmol	–78 °C, Et_2O	30.7	44
L1	0.72 µmol	–78 °C, Et_2O	46.2	66
**Table 1, entry 4**	**0.72 µmol**	**–78 °C, Et_2O**	**33±6**	**55±10**
M1	2.9 µmol	–78 °C, Et_2O	9.5	63
N1	2.9 µmol	–78 °C, Et_2O	10.0	66
**Table 1, entry 5**	**2.9 µmol**	**–78 °C, Et_2O**	**9.7±0.2**	**65±1**
O1	0.15 µmol	–78 °C, Et_2O	76.5	25.5
P1	0.15 µmol	–78 °C, Et_2O	69	23
**Table 1, entry 6**	**0.15 µmol**	**–78 °C, Et_2O**	**73±4**	**24±1**
Q1	No catalyst	–78 °C, Et_2O	<0.05	<1
R1	No catalyst	–78 °C, Et_2O	<0.05	<1
**Table 1, entry 7**	**No catalyst**	**–78 °C, Et_2O**	**<0.05**	**<1**
**S1 Table 1, entry 8**	**No [TBA][CN], [$FeC^{15}N$] precatalyst**	**–78 °C, Et_2O**	**0.7**	**1.1**

Entry	Catalyst loading (μmol) ([FeCN] is catalyst unless specified)	Conditions, (($(C_6H_6)_2Cr$ is the reductant unless specified)	NH_3 (equiv/ Fe)	Yield per reductant (%)[a]
**T1 Table 1, entry 9**	**$FeCl_2$ 8 μmol**	**–78 °C, Et_2O**	**0.3**	**5.5**
**U1 Table 1, entry 10**	**$CrCl_2$ 8 μmol**	**–78 °C, Et_2O**	**<0.03**	**<0.5**
V1	$PhB(CH_2{}^iPr_2P)_3$ FeBr (2.9 μmol)	–78 °C, Et_2O	1.1 +0.4 $MeNH_2$ (equiv/Fe)	9.1
W1	$PhB(CH_2{}^iPr_2P)_3$ FeBr (2.9 μmol)	–78 °C, Et_2O	1.7 +0.85 $MeNH_2$ (equiv/Fe)	15.1
**Table 1, entry 11**	**$PhB(CH_2{}^iPr_2P)_3$ FeBr 2.9 μmol**	**–78 °C, Et_2O**	**1.4±0.7 +0.6±0.2 $MeNH_2$ (equiv/Fe)**	**12±3[b]**
X1	$[P_3{}^BFe]BAr^F{}_4$ 2.9 μmol	–78 °C, Et_2O	2.2	14.7
Y1	$[P_3{}^BFe]BAr^F{}_4$ 2.9 μmol	–78 °C, Et_2O	2.4	16.0
**Table 1, entry 12**	**$[P_3{}^BFe]BAr^F{}_4$ 2.9 μmol**	**–78 °C, Et_2O**	**2.3±0.1**	**15.3±0.6**
Z1	0.72 μmol	25 °C, Et_2O, Cp_2Co reductant	3.6	6
A2	0.72 μmol	25 °C, Et_2O, Cp_2Co reductant	2.0	3.3
**Table 1, entry 13**	**0.72 μmol**	**25 °C, Et_2O, Cp_2Co reductant**	**2.8±0.8**	**4.6±1.3**
B2	0.72 μmol	25 °C, Et_2O, $(Cp^*)_2Cr$ reductant	10.9	18.2
C2	0.72 μmol	25 °C, Et_2O, $(Cp^*)_2Cr$ reductant	16.1	26.8
**Table 1, entry 14**	**0.72 μmol**	**25 °C, Et_2O, $(Cp^*)_2Cr$ reductant**	**13.5±3**	**22±5**
**D2, Table 1, entry 15**	**0.72 μmol**	**25 °C, Et_2O**	**32.4**	**54**
E2	0.72 μmol	25 °C, Et_2O	3.1	5.2
F2	0.72 μmol	25 °C, Et_2O	5.1	8.5
**Table 1, entry 16**	**0.72 μmol**	**25 °C, Et_2O**	**4.1±1**	**7±2**

Additional catalytic runs

Entry	Catalyst loading (μmol) ([FeCN] is catalyst unless specified)	Conditions, (($C_6H_6)_2Cr$ is the reductant unless specified)	NH_3 (equiv/ Fe)	Yield per reductant (%)[a]
A3	0.72 μmol	–78 °C, THF	12.3	
B3	0.72 μmol	–78 °C, THF	16.7	27.8
	0.72 μmol	**–78 °C, THF**	14.5±2.2	**24±4**
C3[c]	2.9 μmol	–78 °C, Et_2O	2.3	46
D3[c]	2.9 μmol	–78 °C, Et_2O	3.6	72
E3[c]	2.9 μmol	–78 °C, Et_2O	4.1	82
	2.9 μmol	**–78 °C, Et_2O**	3.3±0.6	66±12

[c] loadings of 30 equiv $(C_6H_6)_2Cr$ (87 mM)/ 40 equiv $[Ph_2NH_2]OTf$ (116 mM)/ 6 equiv [TBA][CN] (17.4 mM).

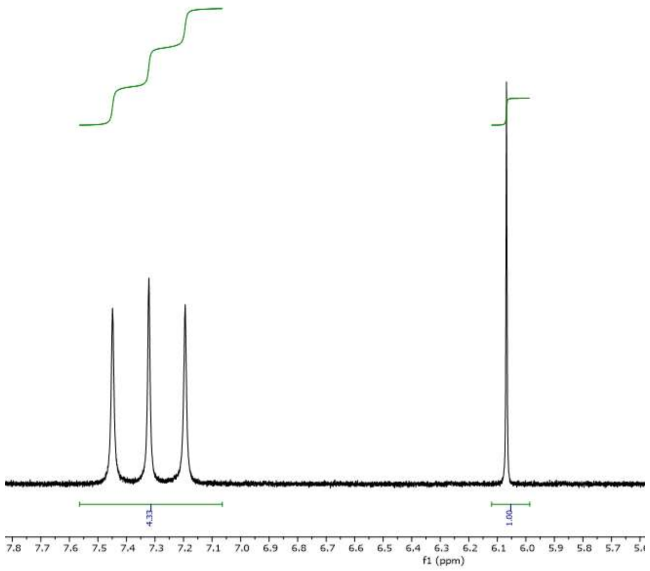

Figure S1. Typical spectra for ^1H NMR detection of $[NH_4]Cl$ (triplet, *J*=52 Hz at 7.31 ppm), with 10 μmol 1,3,5-trimethoxbenzene (TMB) added.

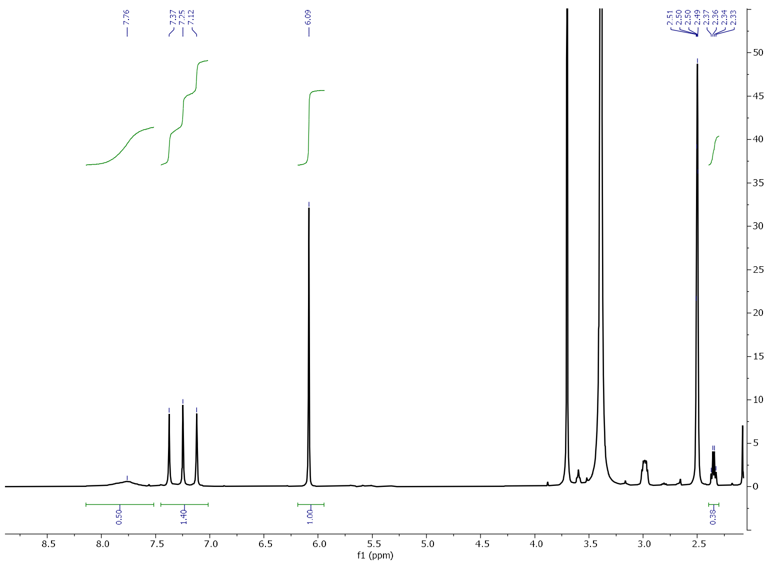

Figure S2. Typical spectra for ^{1}H NMR detection of [NH_4]Cl (δ 7.31 ppm (t), *J*=52 Hz) and [CH_3NH_3]Cl (δ 7.78, br, 3H, δ 2.35, q, *J* = 8 Hz, 3H), with 5 µmol 1,3,5-trimethoxbenzene (TMB) added.

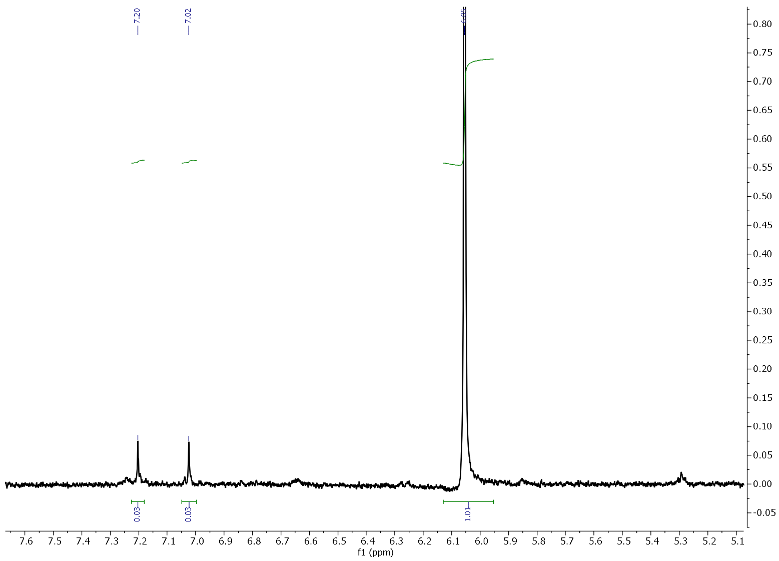

Figure S3. ^{1}H NMR detection of [$^{15}NH_4$]Cl (δ 7.1 ppm (d), *J*=72 Hz) from reduction of [$FeC^{15}N$] (360 equiv $(C_6H_6)_2Cr$ and 480 equiv [Ph_2NH_2]OTf), in the absence of [TBA][CN], quantified with 10 µmol 1,3,5-trimethoxybenzene (TMB) added. Lack of [$^{14}NH_4$]Cl rules out possible background N_2R.

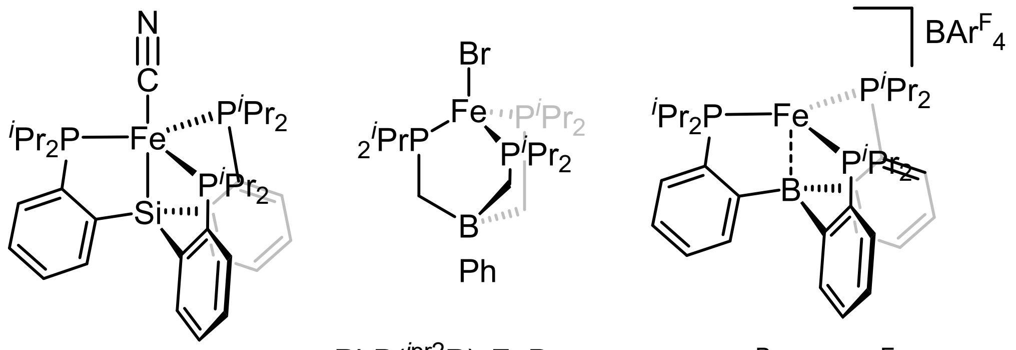

[FeCN] PhB(^{ipr2}P)$_3$FeBr [P_3^BFe][BAr^F_4]

Figure S4. Structures of molecular complexes employed as precatalysts.

S3 Detection and quantification of gaseous products

S3.1 General considerations.

When using standard catalytic conditions, the acid, reductant and cyanide all constitute an appreciable wt% ([Ph_2NH_2]OTf wt. 12 %, $(C_6H_6)_2Cr$ wt. 6 % and [TBA][CN] wt. 4 %) of the reaction mixture and have an unknown effect on the solubility of gaseous organic products. We find that these reagents result in a generally greater amount of CH_4 absorbed in solution than expected for Et_2O alone.

To initially quantify the amount of CH_4 produced, 4 tubes with chemical reagents ([TBA][CN], [Ph_2NH_2]OTf and $(C_6H_6)_2Cr$, standard loadings table 1 entry 1) with and without [FeCN] (0.72 mM) and with and without 500 µL CH_4 added (at 25°C, 1 atm equals 20.5 µmol or 29.4 equiv per [Fe] at 0.72 mM catalyst loading) were prepared. After completion of the reaction an aliquot from the headspace was injected on an HP 5890 GC-FID (30 M column length, 0.280 mm column diameter, He carrier gas), and the respective ratios of the CH_4 peak were compared (retention time=3.7 min). Using these measurements, the CH_4 yields were calculated (Table S2). While we did not quantify C_2 products they were observed as possible minor components in the GC-FID, motivating the use of a gas analysis method on a different instrument that was previously calibrated for quantification of other gases C_2H_4, C_2H_6 and H_2.

With the amount of CH_4 quantified, samples were manually injected on a combined GC-FID/TCD (SRI 8610C, in Multi Gas 5 configuration equipped with HayeSep D and Molsieve 5A columns). Hydrocarbons were detected by a S4 methanizer-flame ionization detector (FID), and the hydrogen was detected by a thermal conductivity detector (TCD). H_2, C_2H_6 and C_2H_4 were calculated with respect to CH_4 (Table S3 and table S4). Using this same internal calibration, gaseous products from reactions run at room temperature and with the tris(phosphine) iron precatalysts were also quantified.

Table S2. Detection of CH_4 with control and leak test

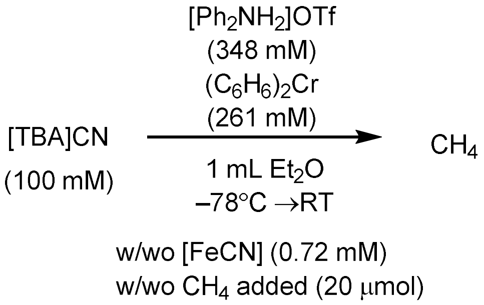

Sample	Signal Area CH_4	Amount of CH_4 added	Amount of CH_4 detected	Amount of CH_4 produced
[Fe]	1754	0 µmol	22.3 µmol	22.0 µmol
[Fe] + 0.5 mL CH_4 (27.8 equiv)	3655	20 µmol	46.4 µmol	26.1 µmol
No Fe	28	0 µmol	0.3 µmol	
No Fe + 0.5 mL CH_4 (27.8 equiv)	1575	20 µmol	20 µmol	
			Average	24±2 µmol
				33±3 CH_4 per Fe

S3.2 Quantification of additional gases

To quantify the additional gaseous products observed a GC-FID/TCD was used, with a previously measured calibration curve. It was assumed that gas absorption (in the reaction mixture) was similar across gasses, as such the gas in the head space was used to calculate the ratios of H_2/CH_4, C_2H_4/CH_4 and C_2H_6/CH_4. Using this same internal calibration gaseous products at room temperature were also quantified in runs C and D, with $^{Ph}BP_3FeBr$ precatalysts in runs D and E and $P_3^BFe[BAr^F_4]$ precatalyst in runs F.

Table S3. Summary of quantification of additional gaseous products.

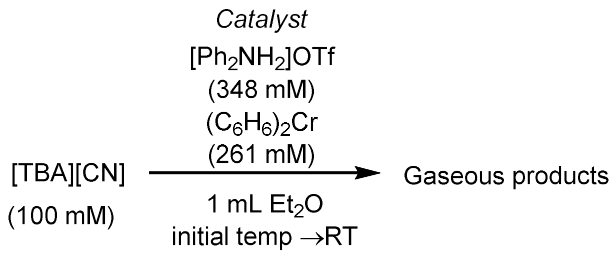

Catalyst (Loading)	Initial T	CH_4 equiv/Fe (yield%/Cr)	H_2 equiv/Fe (yield%/Cr)	C_2H_4 equiv/Fe (yield%/Cr)	C_2H_6 equiv/Fe (yield%/Cr)	C_2/C_1 ratio
[FeCN] (0.575 mM)	−78°C	33±3 (55±5%)	53±15 (29±8%)	0.28±0.02 (0.6±0.04%)	0.21±0.02 (0.6±0.04%)	0.015
[FeCN] (0.575 mM)	25°C	27±4 (45±6%)	53±20 (29±11%)	0.33 (0.7%)	0.26±0.10 (0.7±0.3%)	0.022
$^{Ph}BP_3FeBr$ (2.9 mM)	−78°C	1.0±0.1 (7.3±1%)	5.1±0.1 (11±0.2%)	0.09±0.01 (0.7±0.1%)	0.06±0.01 (0.8±0.1%)	0.11
$(P_3^BFe)[BAr^F_4]$ (2.9 mM)	−78°C	1.6 (11%)	16 (36%)	0.1 (0.1%)	0.1 (0.1%)	0.16

Table S4. Quantification of additional gases and room temperature catalysis. *Headspace concentrations are relative based on GC-calibration.

Gas		CH_4	H_2	C_2H_4	C_2H_6
Run A (20 mL reaction volume)	Area	744	238	13.3	8.7
	Headspace concentration*	3250 ppm	4195 ppm	27 ppm	19.4 ppm
	Ratio to CH_4	-	129%	0.8%	0.6%
Run B (20 mL reaction volume)	Area	762	366	15	10
	Headspace concentration*	3334 ppm	6485 ppm	31 ppm	22 ppm
		-	195%	0.9%	0.7%
Average ratio to CH_4			162±46%	0.85±0.07%	0.65±0.07%
Yield per Fe		33±3	53±15	0.28±0.02	0.21±0.02
Yield per $(C_6H_6)_2Cr$		55±5	29±8%	0.6±0.04%	0.6±0.04%
**Room temperature gas analysis**					
Run C (150 mL reaction volume)	Area	118.9	108.7	2.4	0.6
	Headspace concentration*	406 ppm	995 ppm	4.6 ppm	2 ppm
	Ratio to CH_4 runs(A+B)	92%	220%	1%	0.5%
Run D (150 mL reaction volume)	Area	101.2	54.2	2.4	1.4
	Headspace concentration*	302 ppm	443 ppm	4.6 ppm	4.9 ppm
	Ratio to CH_4 (runs A+B)	70%	100%	1%	1.1%
Average ratio to CH_4			160±60%	1%	0.8±0.3%
Yield per Fe		27±4	53±20	0.33	0.26±0.10
Yield per $(C_6H_6)_2Cr$		45±6	29±11%	0.7%	0.7±0.3%

Gas		CH_4	H_2	C_2H_4	C_2H_6
**Catalytic run with $^{Ph}BP_3FeBr$ (2.9 mM)**					
Run E (20 mL reaction volume)	Area	105.3	109.7	19.4	9.2
	Headspace concentration*	360 ppm	2012 ppm	37 ppm	21 ppm
	Ratio to CH_4 runs(A+B)	11% (0.9 equiv)	61% (5 equiv)	1.1% (0.09 equiv)	0.6% (0.05 equiv)
Run F (20 mL reaction volume)	Area	127.7	114	19	10.8
	Headspace concentration*	434 ppm	2091 ppm	36 ppm	26 ppm
	Ratio to CH_4 (runs A+B)	13% (1.1 equiv)	64% (5.3 equiv)	1.1% (0.09 equiv)	0.8% (0.07 equiv)
Average ratio to CH_4		12±1%	38.5±0.5%	1.1%	0.7±0.01%
Yield per Fe		1.0±0.1	5.1±0.1	0.09±0.01	0.06±0.01
Yield per $(C_6H_6)_2Cr$		7.3±1%	11±0.2%	0.7±0.1%	0.8±0.1%
**Catalytic run with $(P_3^BFe)BAr^F_4$ (2.9 mM)**					
Run G (20 mL reaction volume)	Area	182	356	21.6	12.5
	Headspace concentration*	622 ppm	6530 ppm	41.2 ppm	43.9 ppm
	Ratio to CH_4 runs(A+B)	19% (1.6 equiv)	198%	1.2%	1.3%
Yield per Fe		1.6	16	0.1	0.1
Yield per $(C_6H_6)_2Cr$		10.6%	36%	0.9%	1.1%

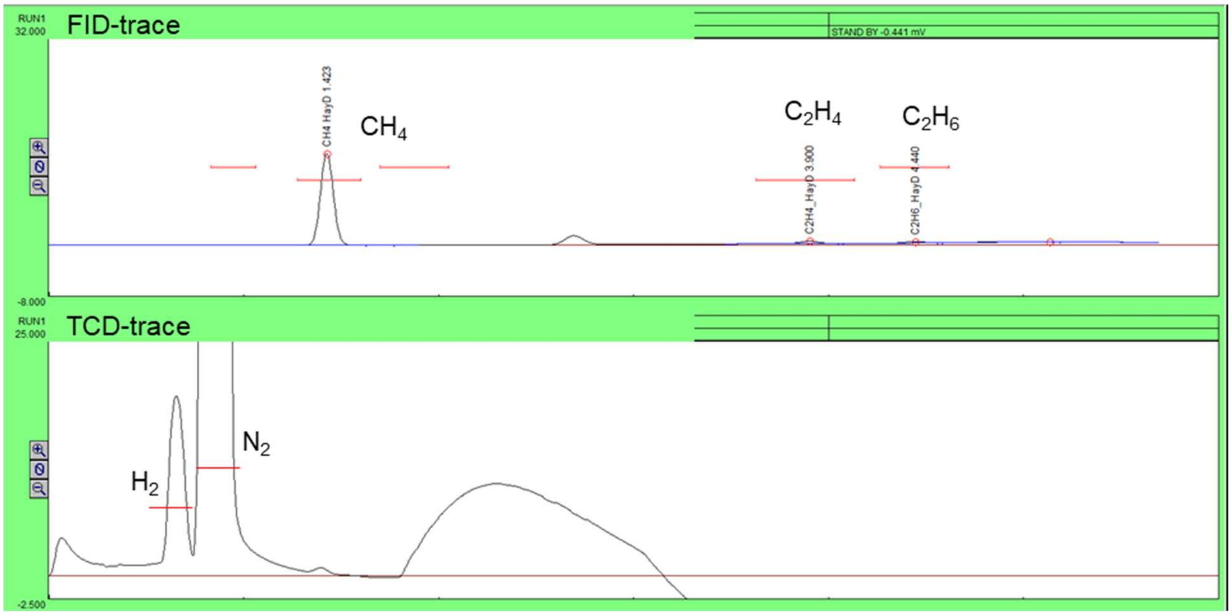

Figure S5. Typical trace for GC-FID (top) and TCD (bottom) of gases detected, with CH_4, C_2H_4, C_2H_6 and H_2 highlighted.

S4 Additional control experiments

S4.1 Monitoring of [TBA]^{13}CN consumption by NMR

To monitor consumption of $[CN]^-$ and formation of methane derived from $[CN]^-$, [TBA][^{13}CN] was used in a ^{13}C NMR experiment. Two J. Young NMR tubes were loaded with [TBA][^{13}CN] (100 mM), $(C_6H_6)_2Cr$ (261 mM) and $[Ph_2NH_2]OTf$ (348 mM), one of these tube was also loaded with [FeCN] (2.9 mM). The tubes were cooled to -78 ˚C, d_8-THF was added, and the tubes were sealed. The tubes were allowed to warm up slowly with occasional mixing (by hand; every 10 minutes) for the first 2 hours of the reaction. Upon completion, analysis by ^{13}C NMR showed that only the tube loaded with [FeCN] had consumed $^{13}CN^-$ and produced $^{13}CH_4$.

The use of THF was required to get sufficient solubility of reaction components, given the worse mixing in the J. Young tube. It was confirmed that setting up catalysis in THF still yields NH_3 (14.5±2.2, Table S1, runs A3 and B3).

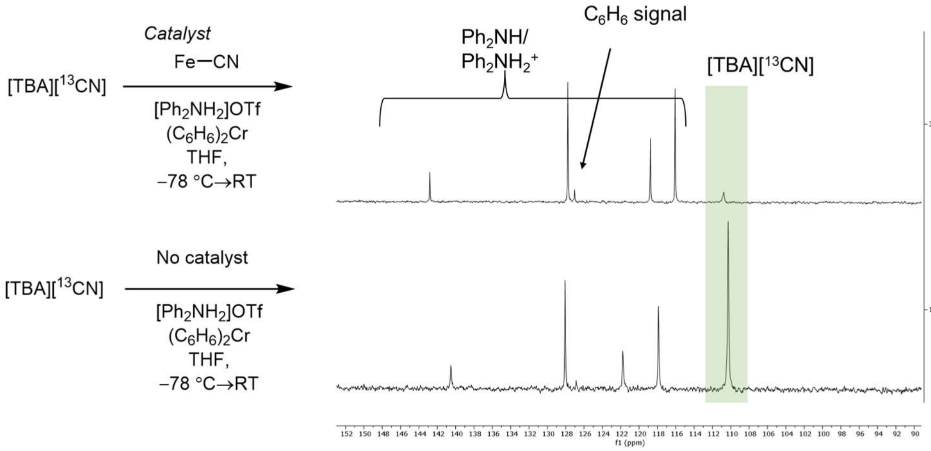

Figure S6. ^{13}C NMR spectra showing reaction of [TBA][^{13}CN] with $(C_6H_6)_2Cr$, $[Ph_2NH_2]OTf$ in the presence and absence of [FeCN] catalyst. As compared to the peaks corresponding to $Ph_2NH/[Ph_2NH_2]^+$ $[^{13}CN]^-$ is only appreciable consumed in the presence of [FeCN] (top spectrum).

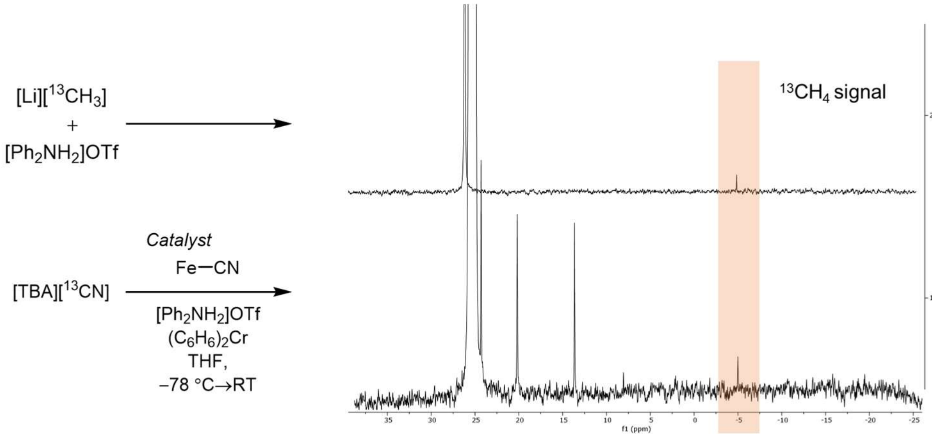

Figure S7. ^{13}C NMR spectra showing reaction of [TBA][^{13}CN] with $(C_6H_6)_2Cr$, $[Ph_2NH_2]OTf$ in the presence of [FeCN] catalyst, with a $^{13}CH_4$ peak (δ – 4.8 ppm, s), as compared to a sample generated upon the addition of acid to [Li][$^{13}CH_3$].

S4.2 Using [FeC^{15}N] precatalyst.

To determine that the precatalyst CN^- ligand is consumed we used [FeC^{15}N] labelled precatalyst, but otherwise set up catalysis as usual (i.e., using natural abundance [TBA][CN]). Low loading conditions were used to not swamp out the ^{15}N signal (2.9 mM FeC^{15}N, 6 equiv [TBA][CN], 30 equiv $(C_6H_6)_2Cr$/ 40 equiv [Ph_2NH_2]OTf). 0.8 equiv [$^{15}NH_4$]Cl is detected along with about 3 equivalents [$^{14}NH_4$]Cl by ^{1}H NMR, consistent with most of the bound [FeC^{15}N] ligand being consumed. Catalysis with similar loading yielded similar amounts of NH_3 (**Table S1** entry C3-E3).

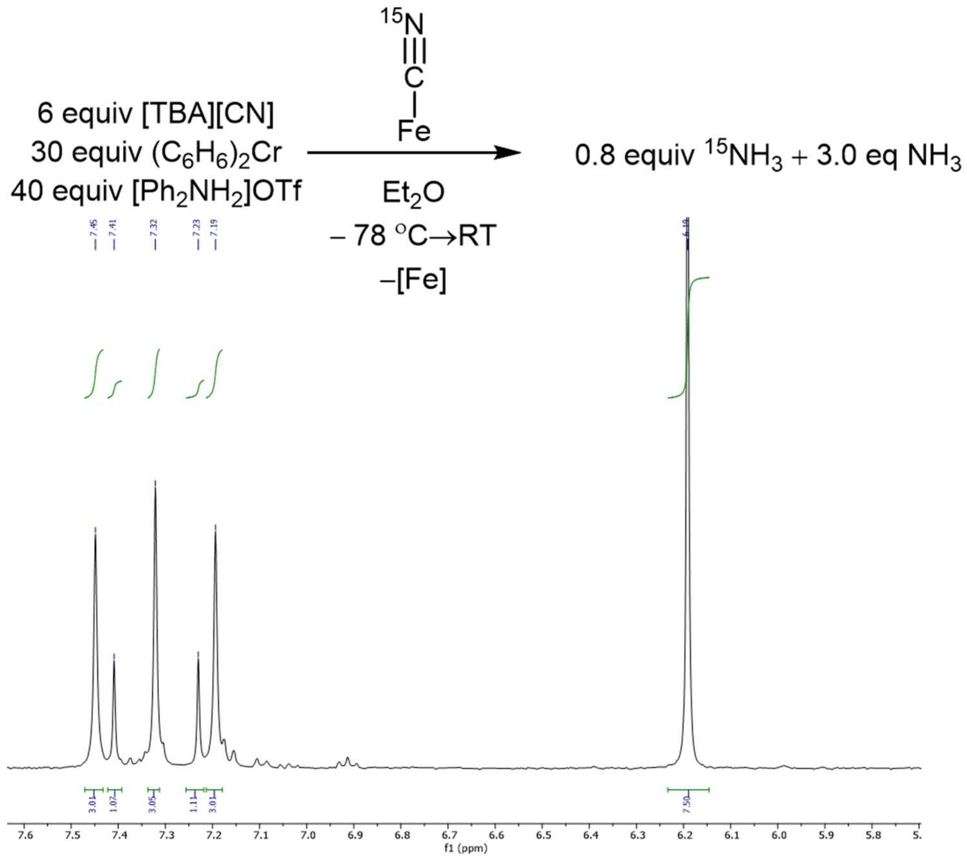

Figure S8. ^{1}H NMR detection of [$^{15}NH_4$]Cl (triplet, J = 52 Hz at 7.32 ppm), and [$^{15}NH_4$]Cl (doublet, J = 72 Hz at 7.32 ppm) with 10 µmol 1,3,5-trimethoxbenzene (TMB) added.

S5 Additional NMR experiments

S5.1 Stoichiometric reactions

S5.1.1 Reduction of [FeCN]

[FeCN] (2 mg, 2.9 μmol) was deposited in a Schlenk flask as a lyophilized powder followed by addition of $(C_6H_6)_2Cr$ (3.6 mg, 6 equiv, 17.4 μmol) and $[Ph_2NH_2]OTf$ (7.4 mg, 8 equiv, 23 μmol). The flask was cooled to −78 °C, and Et_2O (1 mL) was added via syringe. The flask was allowed to warm up slowly overnight, and after completion was brought back into the glovebox. The solvent was removed *in vacuo* and the contents were taken up in C_6D_6 and analyzed by ^1H NMR to find $P_3^{Si}FeOTf$ as the only detectable Fe-species. In a separate experiment, the contents of the flask were worked up as described in section **S2.2**.

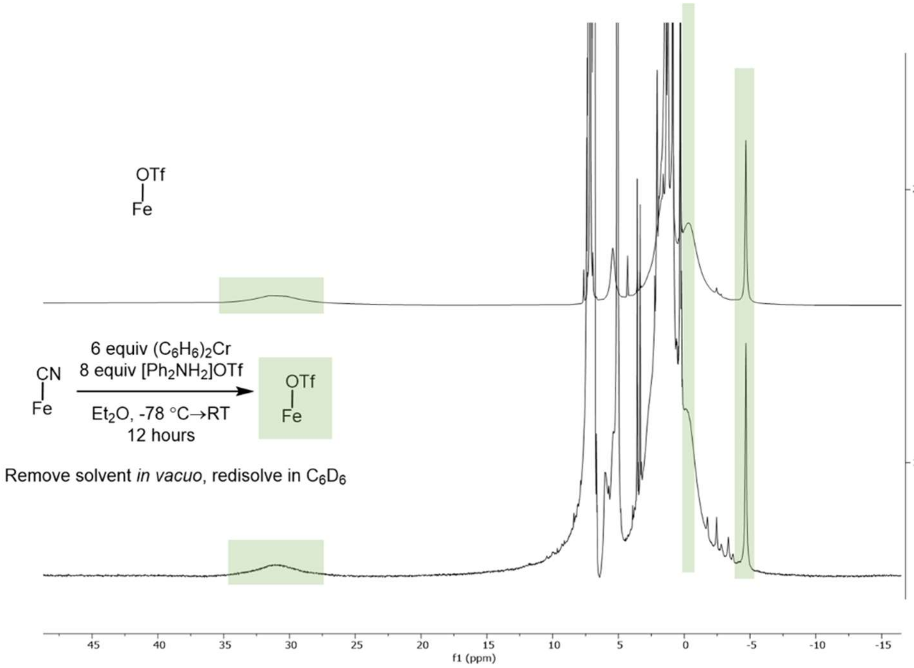

Figure S9. ^1H NMR of reaction of [FeCN] with 6 equiv $(C_6H_6)_2Cr$ and 8 equiv $[Ph_2NH_2]OTf$ in Et_2O. After completed reaction, solvent was removed *in vacuo* and the sample was redissolved in C_6D_6. ^1H NMR showed that the [Fe] product was primarily [FeOTf], green boxes for comparison to authentic sample.

S5.1.2 Metathesis of [FeOTf] to [FeCN]

[FeOTf][8] (5 mg, 6 µmol) and [TBA][CN] (8.8 mg, 10 equiv, 60 µmol) were stirred in Et_2O at room temperature for 5 minutes, color change (orange to red) suggested a change in Fe-speciation consistent with [FeCN] formation. The solvent was removed *in vacuo*, and the contents was redissolved in C_6D_6 to confirm [FeCN] formation by ^1H NMR. ^{19}F NMR showed mostly free TfO^- but some [FeOTf].[8]

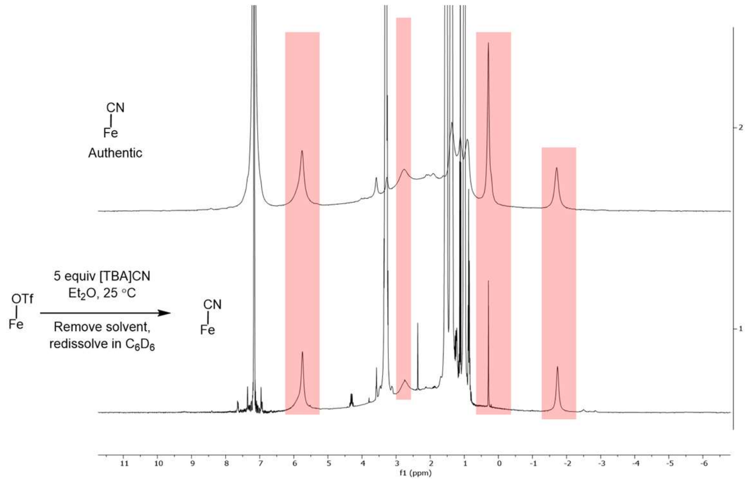

Figure S10. ^1H NMR of reaction of [FeOTf] with 10 equiv [TBA][CN] in Et_2O, stirred at 25 °C for 5 minutes at room temperature. ^1H NMR showed that the [Fe] product was primarily [FeCN], red boxes for comparison to authentic sample.

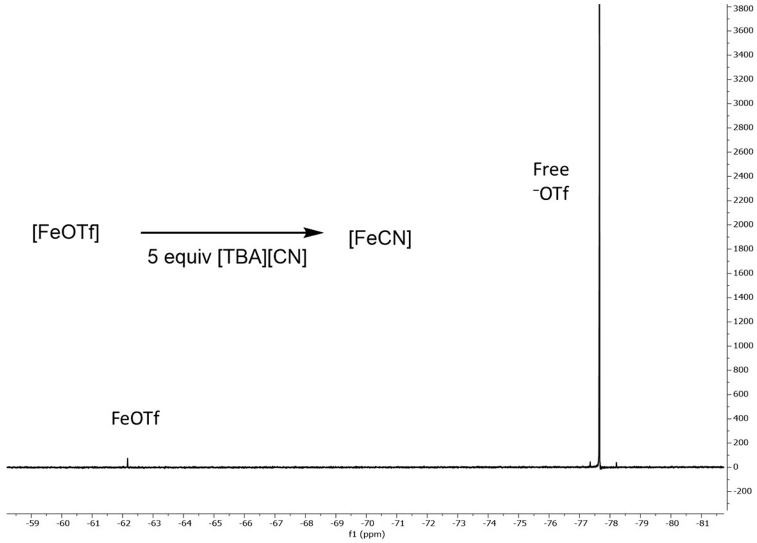

Figure S11. ^{19}F NMR of reaction of [FeOTf] with 10 equiv [TBA][CN] in Et_2O, stirred at 25 °C for 5 minutes at room temperature. ^{19}F NMR showed that the ^{19}F-product was primarily TfO^-.

S5.1.3 Reduction of [FeCN] at room temperature

To confirm the assignment of [FeOTf] in Figure 3 (see main text) a parallel NMR experiment was run. In the drybox, [FeCN] (2 mg, 2.9 µmol) was deposited in a J. Young tube as a lyophilized powder followed by addition of $(C_6H_6)_2Cr$ (12 mg, 20 equiv, 68 µmol) and d_8-THF (0.6 mL). $[Ph_2NH_2]OTf$ (22 mg, 20 equiv, 68 µmol) was added as a solid. Within 5 minutes of acid addition a ^1H NMR of the reaction was collected, confirming formation of [FeOTf]. CH_4 and NH_3 were confirmed in separate experiments, with the reaction being sampled or quenched after a 10 minute reaction time.

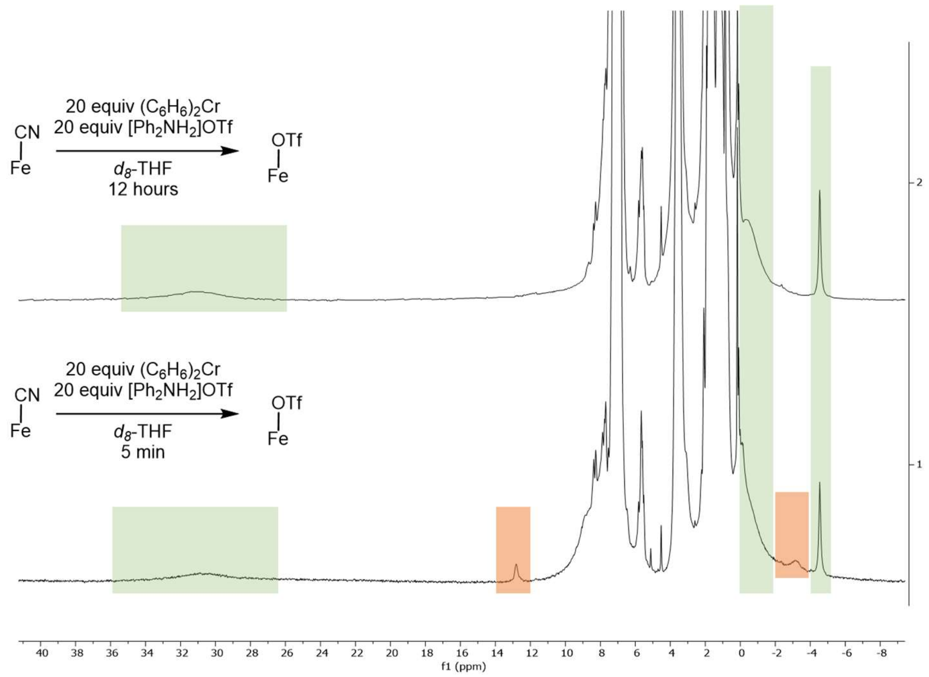

Figure S12. ^1H NMR of reaction of [FeCN] with 20 equiv $(C_6H_6)_2Cr$ and 20 equiv $[Ph_2NH_2]OTf$ in THF. After 5 minutes, multiple Fe-species are formed, including [FeOTf] and an unknown species (orange boxes). Overnight this species disappears and [FeOTf] is the only identifiable Fe-species.

S5.2 Analysis of post-catalysis products

Catalysis was setup as described in section 2.1. To more readily detect [Fe] products the higher loadings (2.9 mM [FeCN]; **Table 1**, entry 5) were used. After completed catalysis (12 hours), the reaction flask was brought back into the glovebox. The flask was filtered, and extracted with additional Et_2O. The solvent was removed *in vacuo*, redissolved in C_6D_6 and analyzed. Fe−H species were detected, notably a doublet of triplets at δ -15.5 ppm. In addition, no [FeOTf] was observed (characteristic peaks δ 31.5, -4.5 ppm were not observed).

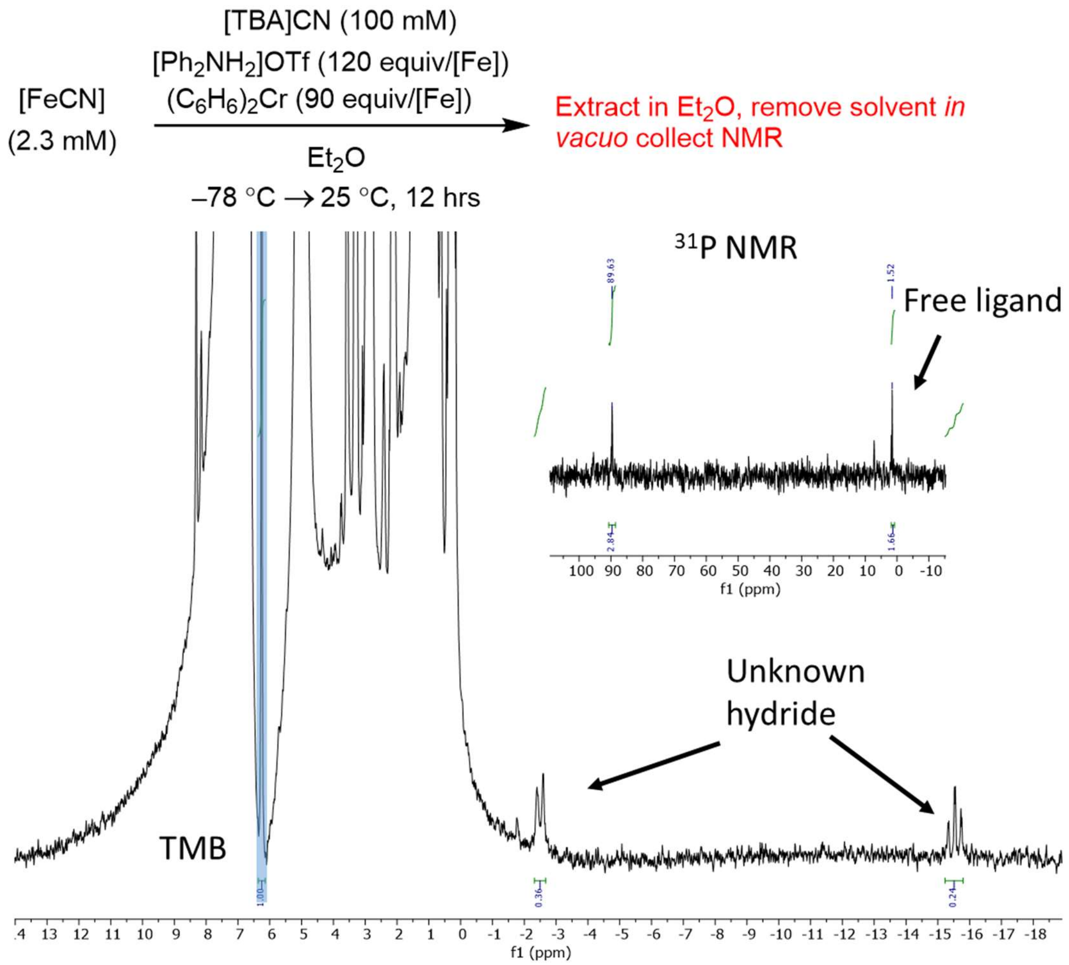

Figure S13. ^{1}H NMR and ^{31}P NMR (inset) detection of extracted post-catalysis products, integrated against 1,3,5-trimethoxbenzene internal standard. Peak at δ -15.5 ppm, td (J = 81.2 Hz; 11.4 Hz) is consistent with a hydride bound to $P_3^{Si}Fe$ with coupling arising from $^2J_{P-H}$ couplings. For comparison $P_3^{Si}Fe(N_2)(H)$ has previously been characterized with td (J = 83.2 Hz; 19.8 Hz).[9]

S5.3 Stability measurements of other iron phosphine precatalysts

To assess possible reasons for poorer performance from the (P_3^B)Fe[BAr$^F_4$] and ($^{Ph}BP_3$)FeBr these catalysts (2.3 mM) were reacted with excess (20 equiv) [TBA][CN] in Et_2O. Analysis of the reaction mixture by ^{31}P NMR after 15 minutes revealed free ligand from both precatalysts. In addition, a low spin Fe-bound product was observed upon the reaction of [TBA][CN] with $^{Ph}BP_3$FeBr, possibly arising from the formation of [$^{Ph}BP_3$Fe(Br)(CN)]$^-$.

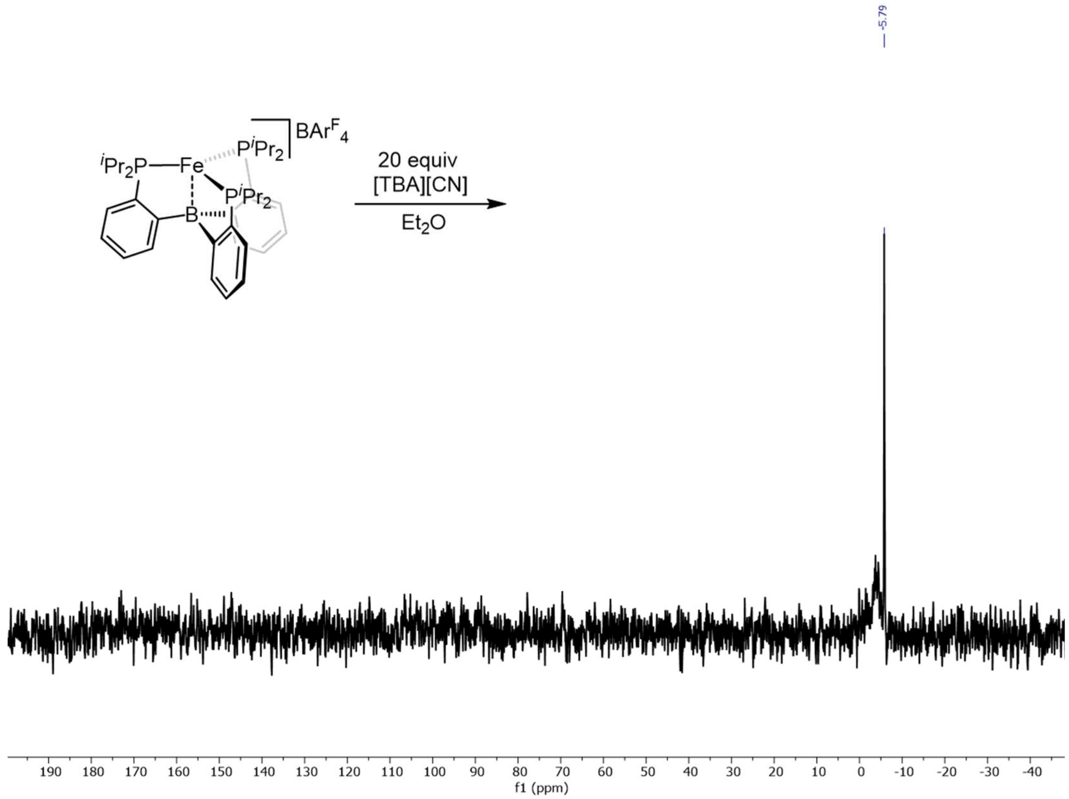

Figure S14. ^{31}P NMR of reaction of (P_3^B)Fe[BAr$^F_4$] with [TBA][CN] in Et_2O. Sharp peak at –6.8 ppm is consistent with unligated phosphine, with another borad peak (–3 ppm) also observed.

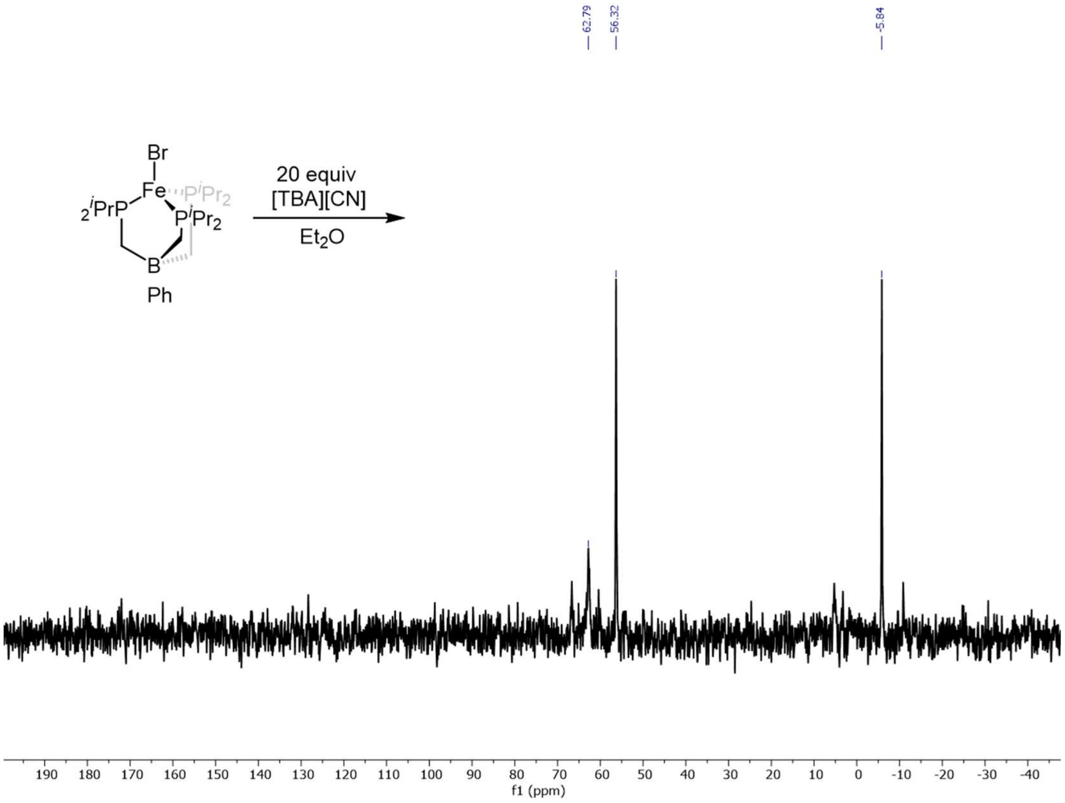

Figure S15. ^{31}P NMR analysis of reaction of ($^{Ph}BP_3$)FeBr with [TBA][CN] in Et_2O. Sharp peak at – 5.9 ppm is consistent with unligated phosphine. Another major peak at 56.3 ppm suggests the formation of diamagnetic Fe-species, perhaps from the formation of formation of $[^{Ph}BP_3Fe(Br)(CN)]^-$ or $[^{Ph}BP_3Fe(Br)(CN)_2]^-$ with the CN^- ligand binding inducing a change of spin state from ($^{Ph}BP_3$)FeBr (S = 1). Additional, small peaks are also observed (65 ppm, 63 ppm, 4 ppm).

S6 Mössbauer data

S6.1 Note on fitting of Mössbauer spectra

Data analysis was performed using version 4 of the program WMOSS (www.wmoss.org) and quadrupole doublets were fit to Lorentzian lineshapes. Simulations were constructed from the minimum number of quadrupole doublets required to attain a quality fit to the data (convergence of χ_R^2). Quadrupole doublets were constrained to be symmetric. Using the non-linear error analysis algorithm provided by WMOSS, the errors in the computed parameters are estimated to be 0.02 mm s^{-1} for δ and 2% for ΔE_Q.

S6.2 Freeze-Quenched Mössbauer spectroscopy of catalytic reactions

S6.2.1 General Procedure for freeze-quench Mössbauer Spectroscopy of catalytic reaction

All solvents are stirred with Na/K for ≥ 2 hours and filtered prior to use. In a nitrogen filled glovebox, the desired ^{57}Fe species (0.0023 mmol) is quantitatively transferred using benzene to a Schlenk flask and then lyophilized to yield a powder. That flask is charged with a stir bar and the other catalytic reagents as solids (analogous to catalytic reduction as described in section **S2.1)**. The tube is then chilled to 77 K in the glovebox cold-well and allowed to equilibrate for five minutes. To the chilled tube is added 1 mL of Et_2O and this flask is allowed to equilibrate for another five minutes. The flask is then transferred to a stir plate where it allowed to thaw. When the stir bar is freed from the frozen solvent and begins to stir the time is started. At the time noted the stirring is stopped and using a pipette the reaction mixture is transferred in one portion to a Mössbauer cup sitting in a vial. The vial is then placed into the 77 K cooled cold well, allowing the reaction mixture to freeze in approximately twenty seconds. The sample is allowed to equilibrate for an additional 20 minutes, before being removed from the glovebox, immediately submerged in liquid nitrogen, and then mounted following standard procedure.

The resulting signals had broad ^{57}Fe Mössbauer spectra, for which meaningful fits could not be readily generated. However, noting the chemistry of [FeCN] in the presence of excess [Ph_2NH_2]OTf and $(C_6H_6)_2Cr$ (**Figures 3** and **S12**) and the known spectra of [FeOTf] (δ = 0.66 mm s^{-1}, ΔE_Q = <0.2 mm s^{-1}, black line in **figure S14** is centered at 0.66 mm s^{-1}) we are reasonably confident that [FeOTf] is present at early times. NMR analysis of the mixtures at 80 minutes show no evidence of [FeOTf] (**Figure S13**).

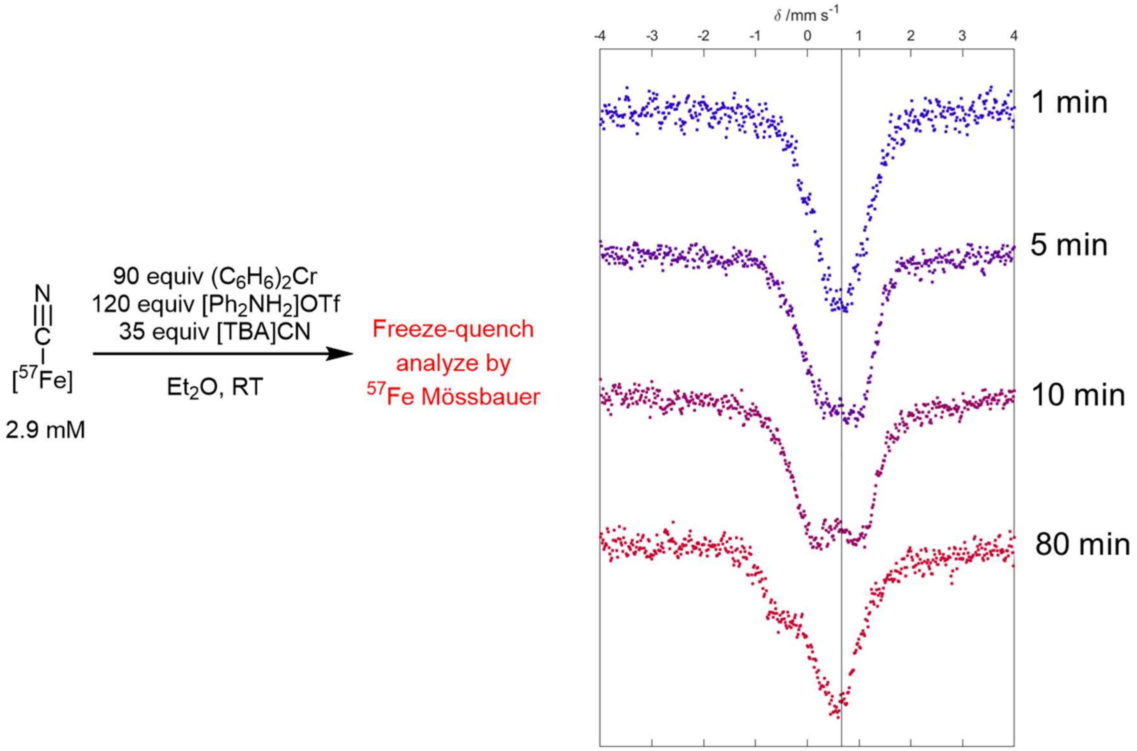

Figure S16. ^{57}Fe mössbauer spectra of freeze-quenched catalytic reactions reacting [^{57}FeCN] (2.9 mM) with 90 equiv $(C_6H_6)_2Cr$, 120 equiv $[Ph_2NH_2]OTf$ and 35 equiv [TBA][CN] in Et_2O for variable time. [FeOTf], reported as a species with $\delta = 0.66$ mm s^{-1}; $\Delta E_Q = <0.2$ mm s^{-1} is observed within 1 minute (Black line at 0.66 mm s^{-1}).[10] Over time other species appear but we have not been able to assign them to known Fe-species.

S6.3 Mössbauer spectroscopy of low temperature reactions of [FeCN] with reductant and acid

S6.3.1 General Procedure for Freeze-Quench Mössbauer Spectroscopy of reactions

All solvents are stirred with Na/K for ≥ 2 hours and filtered prior to use. In a nitrogen filled glovebox, the desired ^{57}Fe species (0.0023 mmol) is dissolved in 0.3 mL Et_2O and cooled to -78 °C in a stirring vial in the glovebox cold well. Acid ($[Ph_2NH_2]BAr^F{}_4$, 0.00575 mmol) was added in 0.1 mL Et_2O. Reductant ($(C_6H_6)_2Cr$ or Cp_2Co, 0.00575 mmol) was added in 0.3 mL to the chilled vial. The reaction was allowed to stir for 5 minutes before it was rapidly transferred to a pre-chilled Mössbauer cup. The cold well was then changed from -78°C (195 K) to 77 K and the solution froze within 5 minutes. The Mössbauer cup was kept at 77 K for minimum 20 minutes before being rapidly taken out of the glovebox and submerged in liquid N_2 and then mounted following standard procedure.

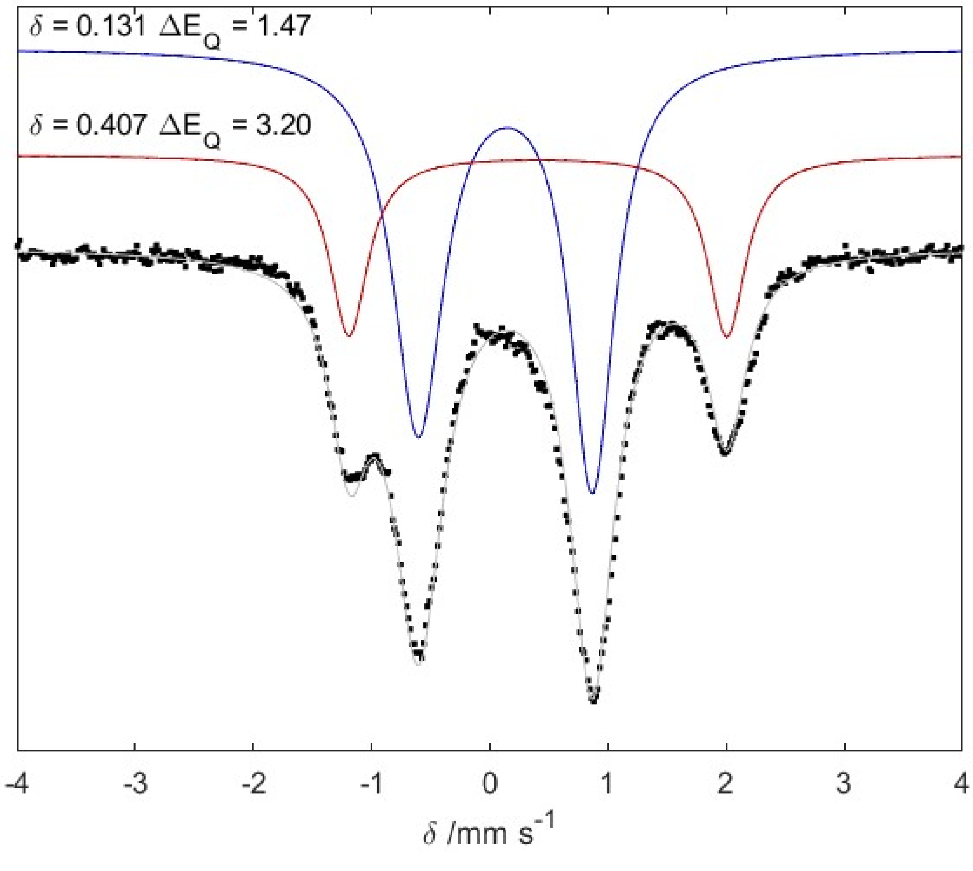

Trace	δ (mm s^{-1})	ΔE_Q (mm s^{-1})	Relative area	Assignment
Blue	0.13±0.01	1.47±0.01	71 %	$[FeCNH_2]^+$
Red	0.41±0.01	3.20±0.01	29%	$[FeCNH]^+$

Figure S17. ^{57}Fe mössbauer spectrum of reaction of $[^{57}FeCN]$ with 2.5 equiv each of $(C_6H_6)_2Cr$ and $[Ph_2NH_2]BAr^F{}_4$. Spectra fit as two species.

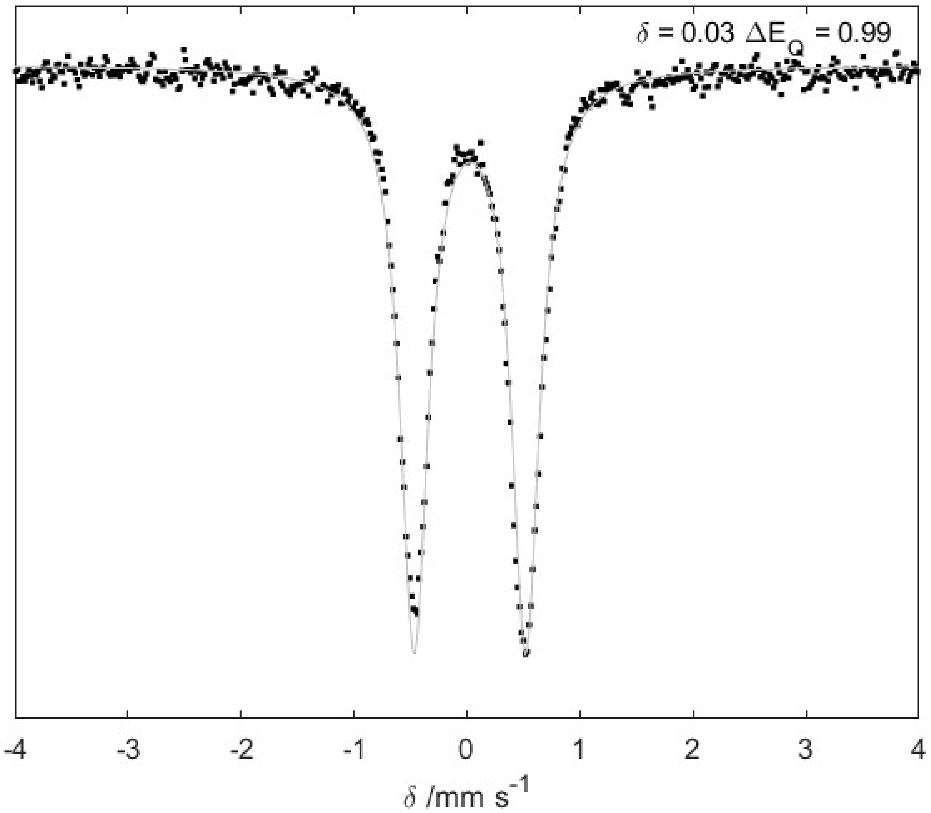

Figure S18. ^{57}Fe mössbauer spectrum of reaction of [^{57}FeCN] with 2.5 equiv each of Cp_2Co and [Ph_2NH_2]BAr^F_4. Spectra fit as a single species, $\delta = 0.03\pm0.01$ mm s^{-1}, $\Delta E_Q = 0.99\pm0.01$ mm s^{-1}

S6.4 Alternative synthesis of [$FeCNH_2$] Mössbauer sample

S6.4.1 Synthetic procedure

[FeCN] (20 mg, 29 μmol) was dissolved in 0.5 mL THF in a 20 ml vial. The solution was cooled to −78 °C, and excess KC_8 was added. The reaction mixture was then filtered through a pipette loaded with a filter paper, Celite and KC_8 3 times to form (proposed) [FeCN]K_2. The reaction mixture was filtered a final time to remove graphite and excess KC_8, giving a jet-black solution. This *in situ* formed material was used directly to form [$FeCNH_2$]. Alternatively, transferring this solution to a Mössbauer cup while maintaining the temperature, freezing the reaction mixture at 77 K and analyzing by ^{57}Fe Mössbauer revealed a Mössbauer spectra with parameters $\delta = 0.20$ mm s^{-1}, $\Delta E_Q = 0.18$ mm s^{-1} (**Figure S19 A**), with a minor impurity.

For generation of [$FeCNH_2$], 2 equiv [$H(OEt_2)_2$]BAr^F_4 (59 mg, 58 μmol) dissolved in 0.3 mL THF, was added slowly to *in situ* formed [FeCN]K_2, with an orange solution formed. Transferring this solution to a Mössbauer cup while maintaining the temperature, freezing the reaction mixture at 77 K and analyzing by ^{57}Fe Mössbauer revealed a Mössbauer spectra with parameters $\delta = 0.02$ mm s^{-1}; $\Delta E_Q = 0.96$ mms^{-1} (**Figure S19 B**), with some oxidized material also formed. The parameters of the major species fit those observed for [$FeCNH_2$] synthesized with [Ph_2NH_2]BAr^F_4 and Cp_2Co.

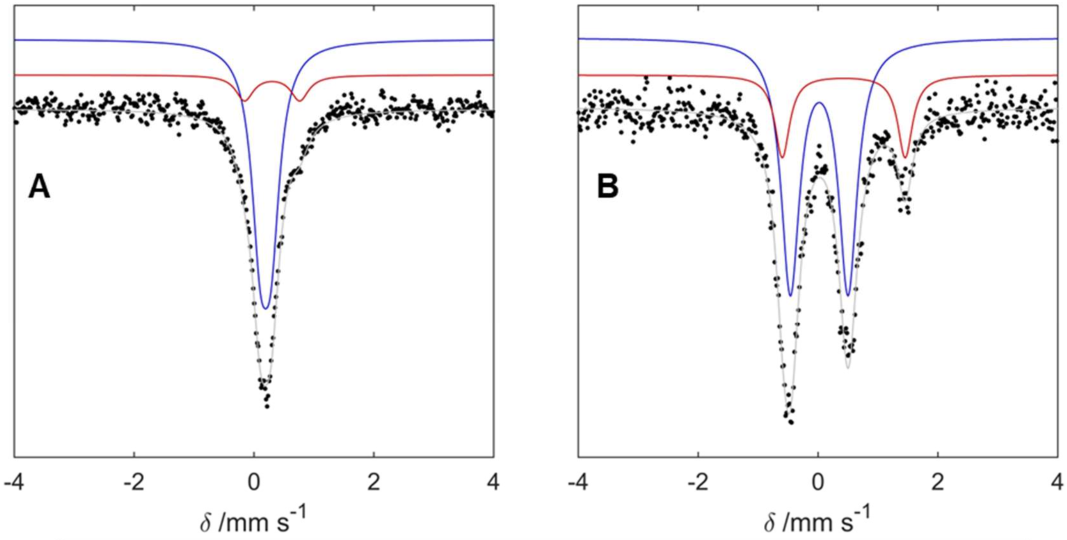

^{57}Fe spectra A			
δ(mm s^{-1})	ΔE_Q (mm s^{-1})	Γ (mm s^{-1})	Rel. area
0.20±0.01	0.18±0.05	0.35±0.05	86% (Blue)
0.30±0.01	0.92±0.05	0.36±0.03	14% (Red)

^{57}Fe spectra B

δ(mm s^{-1})	ΔE_Q (mm s^{-1})	Γ (mm s^{-1})	Rel. area
0.02±0.01	0.96±0.01	0.37±0.01	80% (Blue)
0.43±0.01	2.09±0.01	0.30±0.01	20% (Red)

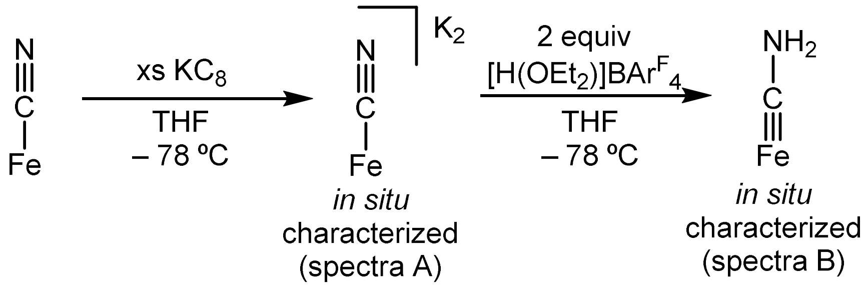

Figure S19. ^{57}Fe mössbauer of reaction of [^{57}FeCN] with KC_8 (excess) to form proposed [^{57}FeCN]K_2 (spectra A) and reaction of proposed [^{57}FeCN]K_2 with 2 equiv [$H(OEt_2)_2$]BAr^F_4 to form [$FeCNH_2$] (spectra B).

S7. UV-visible measurements

S7.1 General procedure for room temperature reduction of [FeCN] monitored by UV-vis spectroscopy

A 1 cm path length cuvette was loaded with [FeCN] (1 mM) and $(C_6H_6)_2Cr$ (20 mM) in THF in the glovebox. The solution was sealed with a septum with N_2 flowed through the septum to maintain an O_2 free atmosphere. To this solution $[Ph_2NH_2]OTf$ was rapidly added via syringe and the reaction was monitored collecting a spectrum every 6 seconds.

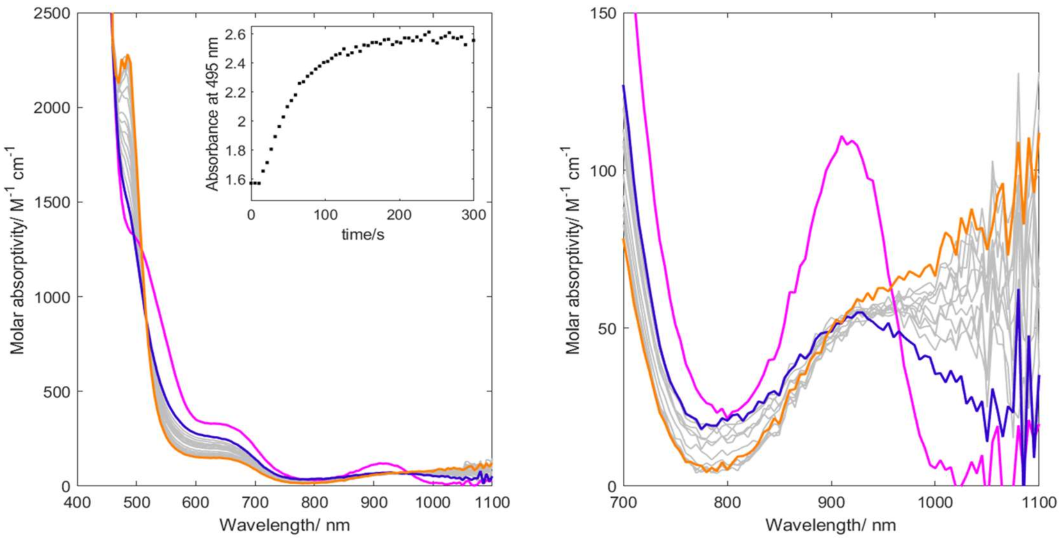

Figure S20. Inset shows absorbance peak at 495 nm, which has previously been noted as characteristic for [FeOTf], monitoring of this peak gave a $\tau_{1/2}$ = 40 s. Zoomed out spectrum shows isosbestic point at 510 nm and sharp absorption at 495 nm characteristic of [FeOTf].

S7.2 General procedure for low temperature UV-vis spectroscopy (Figure 4 b and c).

A 1 cm path length cuvette was loaded with [FeCN] (1 mM) in THF in the glovebox. It was sealed with a septum. It was brought out and allowed to equilibrate at the given temperature for 20 minutes. The temperature was maintained at -80°C, with constant stirring during data collection. The acid was dissolved in 0.5 mL THF and added to the temperature equilibrated cuvette. This resulted in immediate formation of $[FeCNH]^+$ (**Figure S21**). Then 2 equivalents of reductant were dissolved in 1 mL THF in the glovebox, brought out of the glovebox and titrated into the solution via syringe. A spectrum was collected 2 minutes after each addition of reductant. In the case of $(C_6H_6)_2Cr$ an additional 4 equivalents of reductant were dissolved in an additional 1 mL of solvent to continue the titration. The spectra were plotted against molar absorptivity thereby adjusting for the change of concentration of [Fe] over the course of the titration (Figure 4B and C).

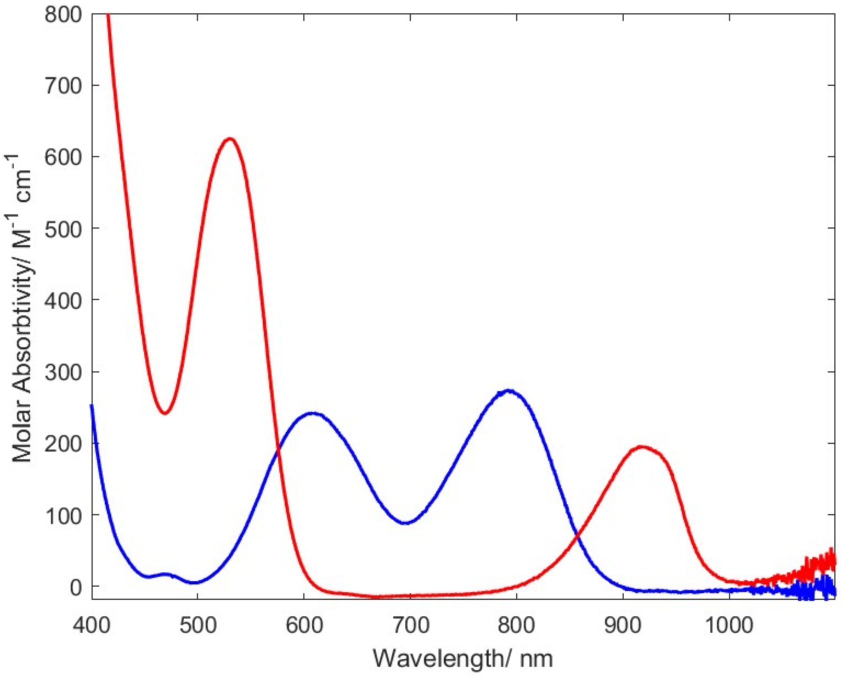

Figure S21. Spectrum of starting [FeCN] (red) and after addition of $[Ph_2NH_2]BAr^F_4$ (blue) in THF at -80 °C. Features match what has been previously observed for [FeCN] and $[FeCNH]^+$.[1,10]

S8. Electrochemical measurements

S8.1 General procedure

Electrochemical measurements were conducted with a glassy carbon working electrode, with a platinum wire counter electrode and Ag/AgOTf (1mM AgOTf in 0.2 M [TBA][OTf]) reference electrode isolated by a CoralPor™ frit (obtained from BASi) and referenced externally to $Fc^{+/0}$. Unless otherwise specified, NaK dried THF was used as solvent, with 0.2 M [TBA]OTf electrolyte. Measurements were conducted with a CH Instruments 600B electrochemical analyzer.

S8.2 Acid reduction current with different reductants

To test the ability of each reductant to perform the hydrogen evolution reaction, we used electrochemical methods. Beginning the oxidized form of the reductants ([red]BAr^F_4 ; red = $(C_6H_6)_2Cr$, Cp_2Co, $Cp^*{}_2Cr$) for each [red]BAr^F_4 the current associated with reduction is enhanced upon addition of [Ph_2NH_2]OTf. The increase in current is proposed to occur due to a catalytic EC mechanism (**Figure S20**), with reduction of [red]BAr^F_4 being followed by protonation and hydrogen evolution. The increase in current upon the addition of acid to each reductant becomes a measure of the rate of reaction between the reductant and [Ph_2NH_2]OTf, proposed to reflect rate of background hydrogen evolution during catalysis.

[red]BAr^F_4 (Red = $(C_6H_6)_2Cr$, Cp_2Co, $Cp^*{}_2Cr$) was prepared in an analogous way to [$Cp^*{}_2Co$]BAr^F_4 [7] previously. 1 mM solutions were prepared in [TBA]OTf electrolyte (0.2 M) in THF. Acids were added as solids to avoid change in concentration of [red]BAr^F_4.

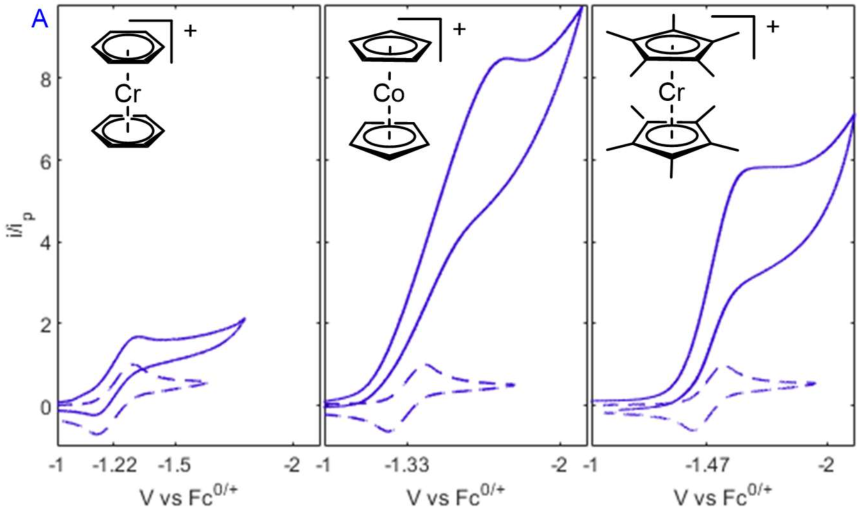

B

Reductant	$E°$ (V vs $Fc^{+/0}$)	i_{cat}/i_p	TON (NH_3)
$(C_6H_6)_2Cr$	-1.22	1.7	27 ± 6
Cp^*_2Cr	-1.47	5.8	13.5 ± 3
Cp_2Co	-1.33	8.5	2.8 ± 0.8

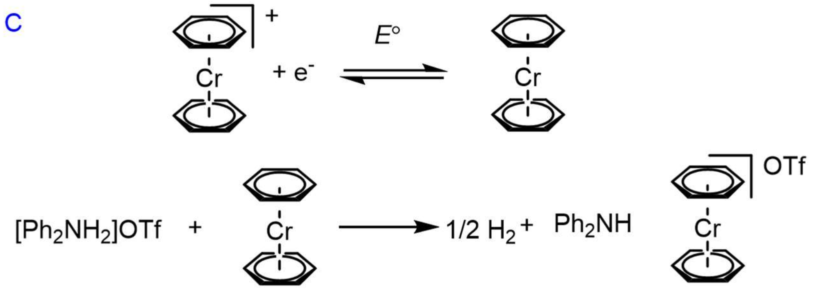

Figure S22. (A): Cyclic voltammograms of [red]BAr^F_4 (1 mM) in 0.2 M [TBA]OTf, with 0 equiv [Ph_2NH_2]OTf (dashed) and 50 equiv [Ph_2NH_2]OTf (solid lines) (100 mv s^{-1} scan rate). After addition of [Ph_2NH_2]OTf catalytic current is observed, but to a differing extent. The catalytic current is a measure for the rate of reaction between acid and reductants. (B): Comparing the acid free current to the catalytic proton reduction current (i_{cat}/i_p) we find that i_{cat}/i_p correlates inversely to TON (NH_3), with $(C_6H_6)_2Cr$ > Cp_2Co > Cp^*_2Cr being the order of NH_3 TON and $(C_6H_6)_2Cr$ < Cp_2Co < Cp^*_2Cr being the order of i_{cat}/i_p. Interestingly there is no correlation between i_{cat}/i_p and the reduction potential of the different reductants. (C) Proposed mechanism for electrochemical acid reduction, mediated by [red]BAr^F_4, exemplified for red = $(C_6H_6)_2Cr$.

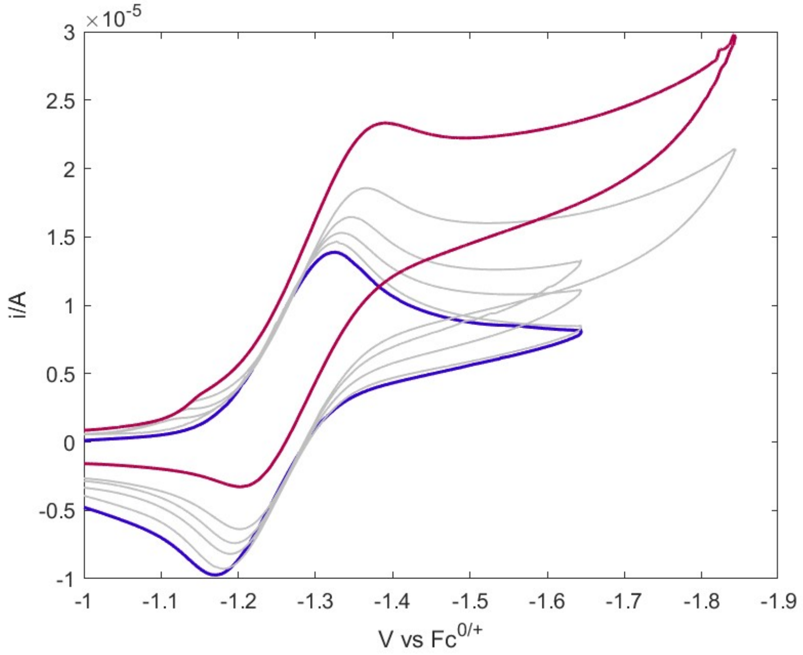

Figure S23. (A): Cyclic voltammograms of $[(C_6H_6)_2Cr]BAr^F_4$ (1 mM, dashed lines) in 0.2 M [TBA]OTf, with increasing $[Ph_2NH_2]$OTf added from 0 (blue trace) to 50 equiv (red trace) at 100 mV s^{-1} scan rate.

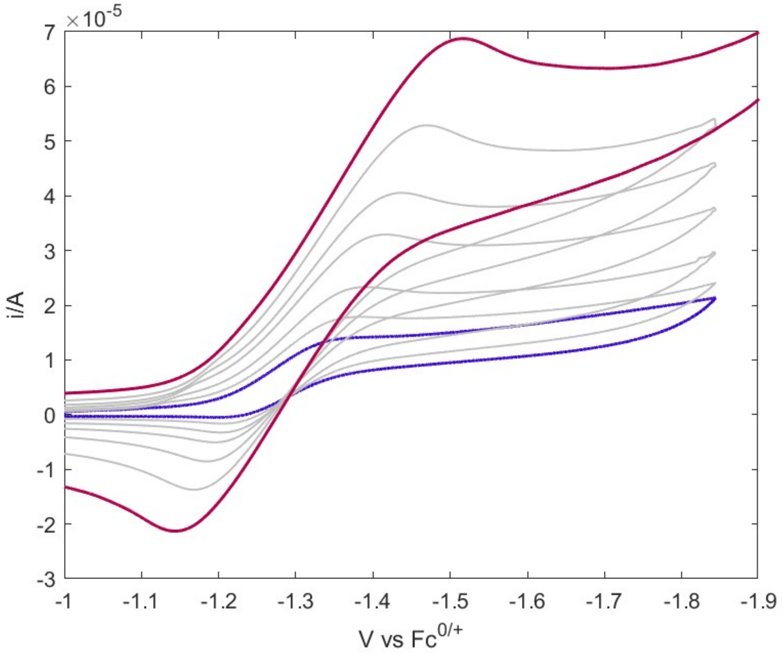

Figure S24. (A): Cyclic voltammograms of $[(C_6H_6)_2Cr]BAr^F_4$ (1 mM, dashed lines) in 0.2 M [TBA]OTf, with 50 equiv $[Ph_2NH_2]$OTf added, with varying scan rate from 25 (blue trace) to 1600 mv s^{-1} (red trace).

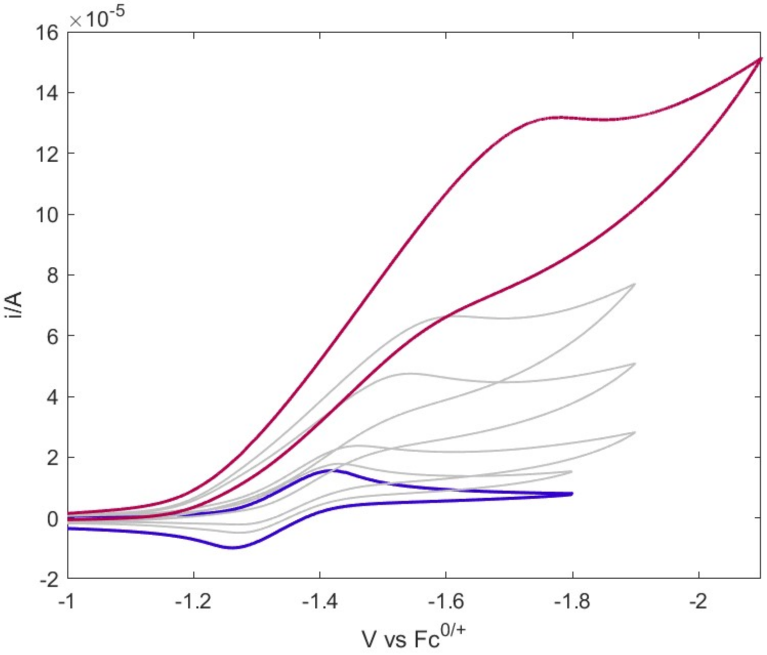

Figure S25. (A): Cyclic voltammograms of $[Cp_2Co]BAr^F_4$ (1 mM, dashed lines) in 0.2 M [TBA]OTf, with increasing $[Ph_2NH_2]OTf$ added from 0 (blue trace) to 50 equiv (red trace) at 100 mV s^{-1} scan rate.

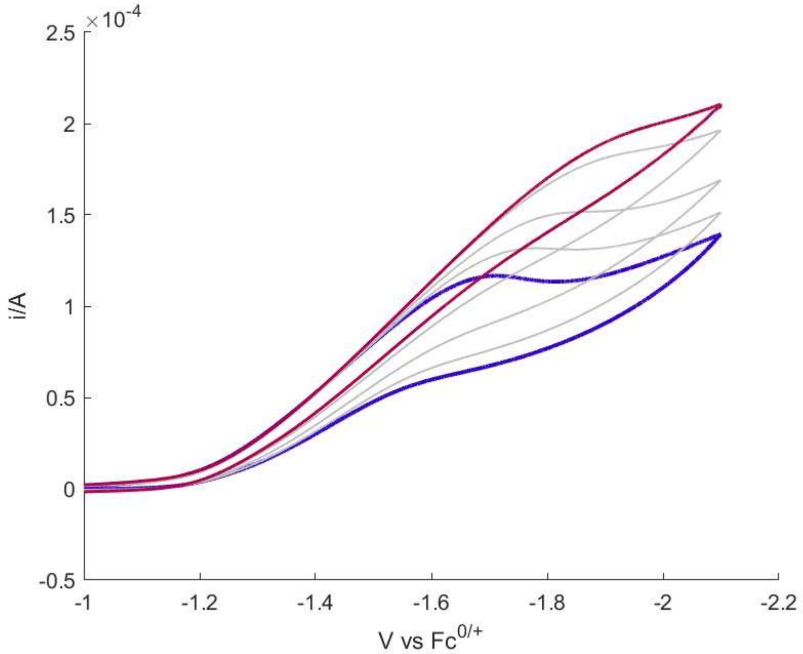

Figure S26. (A): Cyclic voltammograms of $[Cp_2Co]BAr^F_4$ (1 mM, dashed lines) in 0.2 M [TBA]OTf, with 50 equiv $[Ph_2NH_2]OTf$ added, with varying scan rate from 50 (blue trace) to 800 mv s^{-1} (red trace).

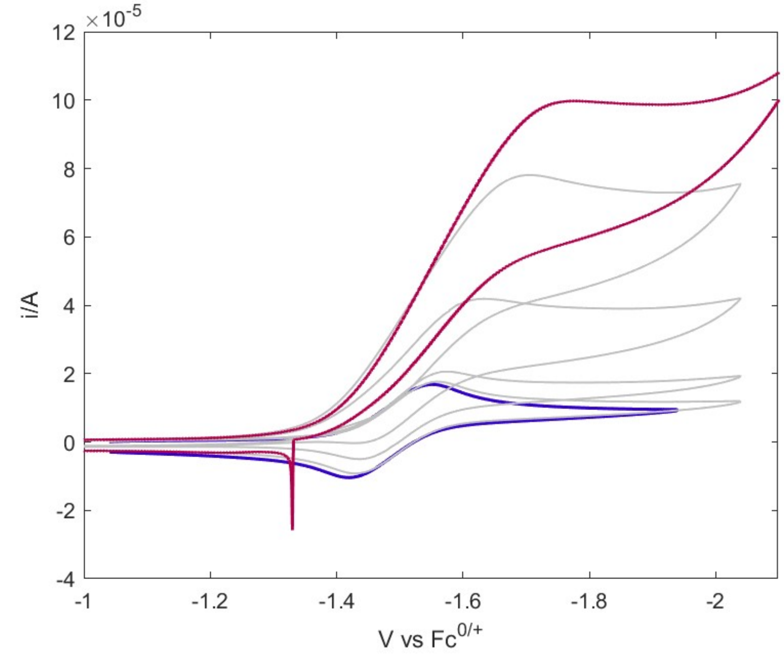

Figure S27. (A): Cyclic voltammograms of [Cp^*_2Cr]BAr^F_4 (1 mM, dashed lines) in 0.2 M [TBA]OTf, with increasing [Ph_2NH_2]OTf added from 0 (blue trace) to 50 equiv (red trace) at 100 mV s^{-1} scan rate.

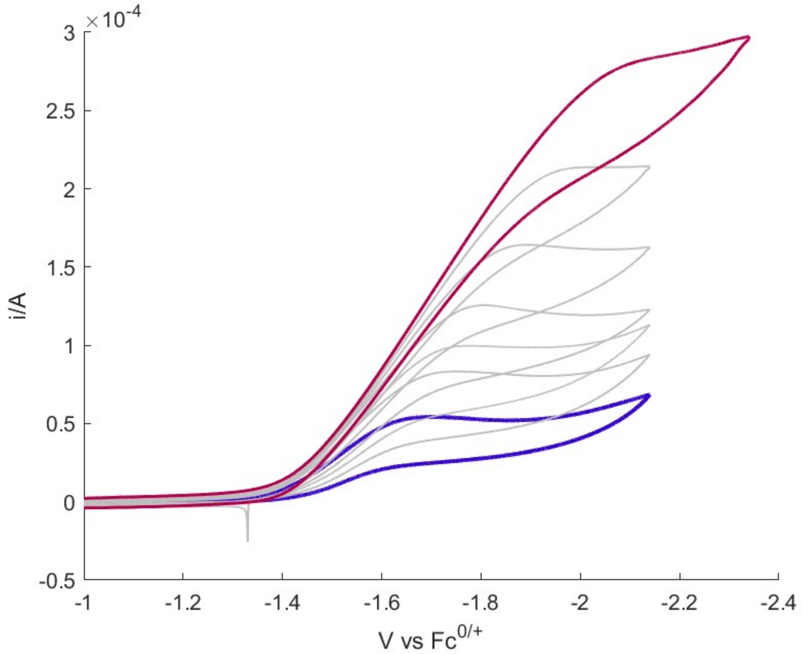

Figure S28. (A): Cyclic voltammograms of [Cp^*_2Cr]BAr^F_4 (1 mM, dashed lines) in 0.2 M [TBA]OTf, with 50 equiv [Ph_2NH_2]OTf added, with varying scan rate from 25 (blue trace) to 1600 mv s^{-1} (red trace).

S8.3 Electrochemical mechanistic studies with [FeCN]

Studies of the electrochemistry of [FeCN] support that catalysis can occur with weak reductants like $(C_6H_6)_2Cr$. We have previously measured the reduction potential of $[FeCN]^{0/-}$ at E_{red}= –2.07 V vs $Fc^{+/0}$,[1] which is not accessible using $(C_6H_6)_2Cr$ reductant (E_{ox}= –1.22 V). Upon addition of $[Ph_2NH_2]OTf$ to a solution of $[Fe^{II}CN]$ in THF with 0.2 M $[TBA]PF_6$ electrolyte at 25 °C, we observed that this Fe^{II}/Fe^{I} couple shifts to $E° \approx -1.2$ V (**Figure S29**, inset), close to what has been previously observed for **[**$FeCNH]^{+}[BAr^F{}_4]$ (E_{red}= –1.27 V).[1]

Upon closer inspection, this new reductive couple was composed of two features that partially overlap, with current maxima at E_p = –1.15 V and E_p = –1.28 V (**Figure S29**)., attributed to $E([FeCNH]^{+/0})$ and $E([FeCNH_2]^{+/0})$ respectively. The irreversibility of the first peak ($E°_{1/2}$= –1.10 V) is attributed to the rapid protonation of electrochemically generated [FeCNH]. The second peak ($E°_{1/2}$= –1.23 V) is irreversible at high acid loadings and slow scan rates (**Figure S30**), which is consistent with a multielectron process occurring after the protonation of electrochemically generated **[FeCNH₂]**. Using normalized current $i_{normalized} = i/\sqrt{v}$ shows a decrease in current with increasing scan rate, as expected for a catalytic current.

These cyclic voltammetry experiments support our proposed early steps for catalysis. It is worth noting that the measured reduction potential of $[FeCNH_2]^{+/0}$ (−1.23 V) would suggest the formation of an equilibrium of these species when $(C_6H_6)_2Cr$ (−1.22 V) is employed as a reductant, as we observe experimentally (Figure 4). Based on this analysis we cite the ***E*°(**$[FeCNH_2]^{+/0}$) as ~−1.2 V in the main text. The electrochemical and chemical reactions as described are summarized in **Figure S31**.

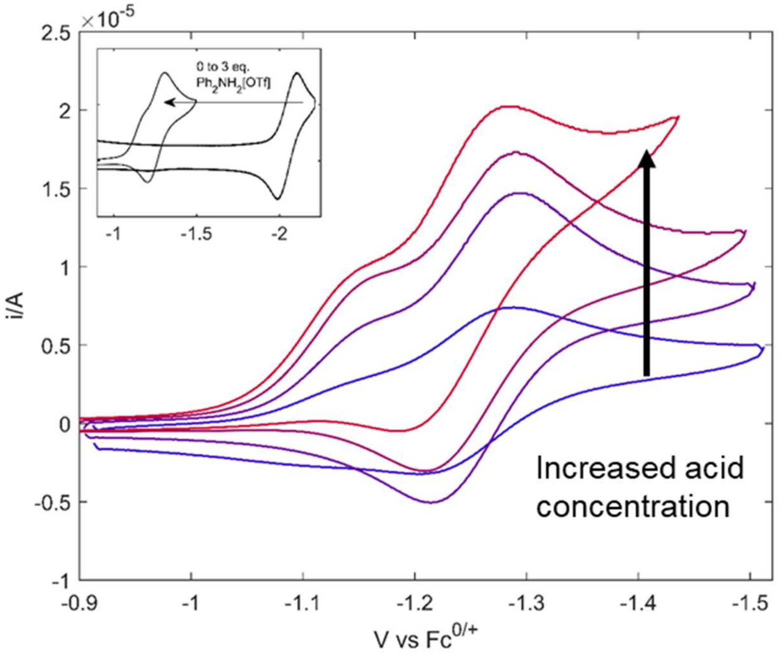

Figure S29. Cyclic voltammograms monitoring the titration of $[Ph_2NH_2]OTf$ to [FeCN], at 100 mV s^{-1} scan rate: 0 to 3 equiv shown in inset, 3 equiv to 20 equiv shown in main figure.

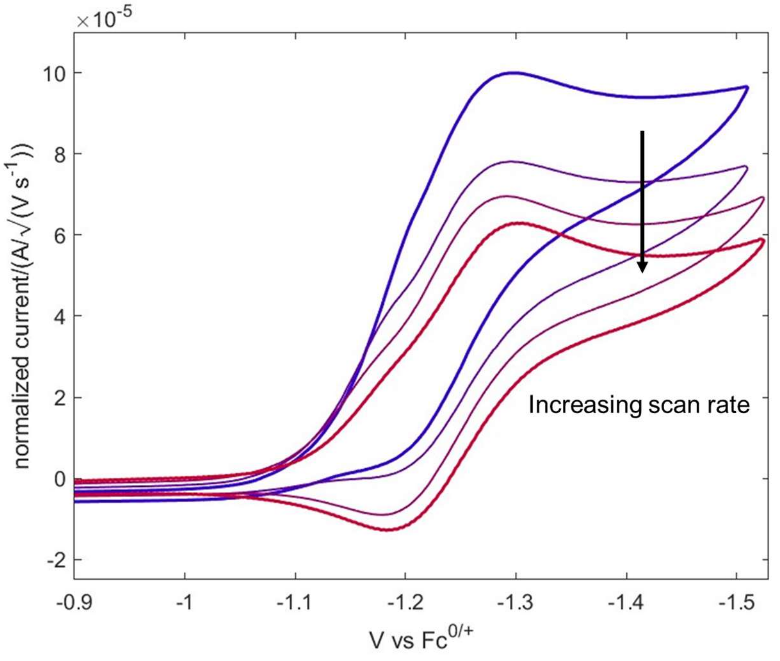

Figure S30. Cyclic voltammograms monitoring scan rate dependance for [FeCN] reduction in the presence of 20 equiv [Ph_2NH_2]OTf from 10 mV s^{-1} to 75 mV s^{-1}. Normalized current decreases with scan rate as expected for a multielectron process.

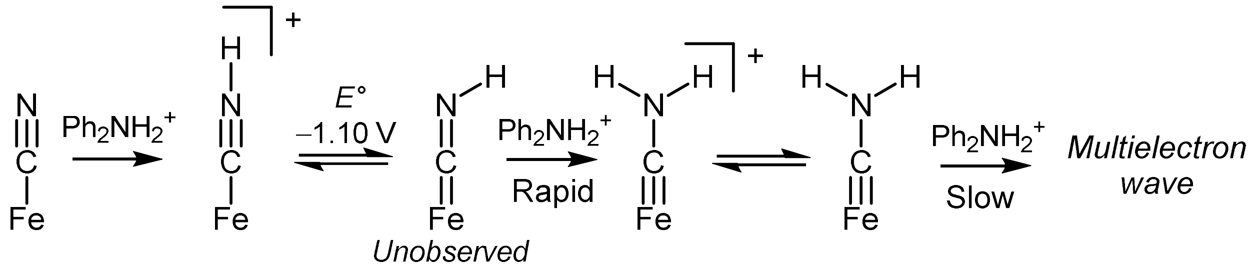

Figure S31. Proposed scheme for cyclic voltammetry measurements

S9. Generation of proposed $[FeC(H)(NMe_2)]^+$

For ease of the reader ^{1}H-NMR, UV-vis and ^{57}Fe Mössbauer data associated with the synthesis of the proposed carbene species $[FeC(H)(NMe_2)]^+$ are all collated in **section S9**, instead of having the individual spectra in the appropriate spectroscopic sections.

S9.1 Generation of UV-vis spectrum of $[FeC(H)(NMe_2)]^+$ in the absence of reductant.

A cuvette of dilute $[FeCNMe_2]$[1] (1 mM) in THF was prepared in the glovebox and sealed with a septum. It was brought out of the glovebox and cooled to 0 °C, with a positive flow of N_2 supplied with a needle. After equilibration, 5 equiv $[Ph_2NH_2]OTf$ in 0.25 mL THF were added by syringe, and the reaction was monitored. Within 20 minutes the initial peak at 420 nm had been consumed with new maxima appear at 585 nm and 790 nm, as well as a feature that grows beyond the detection limit of the spectrometer (1100 nm). The addition of 10 equiv strong base (TBD) resulted in a loss of these new features and restoration of the initial absorbance at 420 nm, diagnostic for $[FeCNMe_2]$.

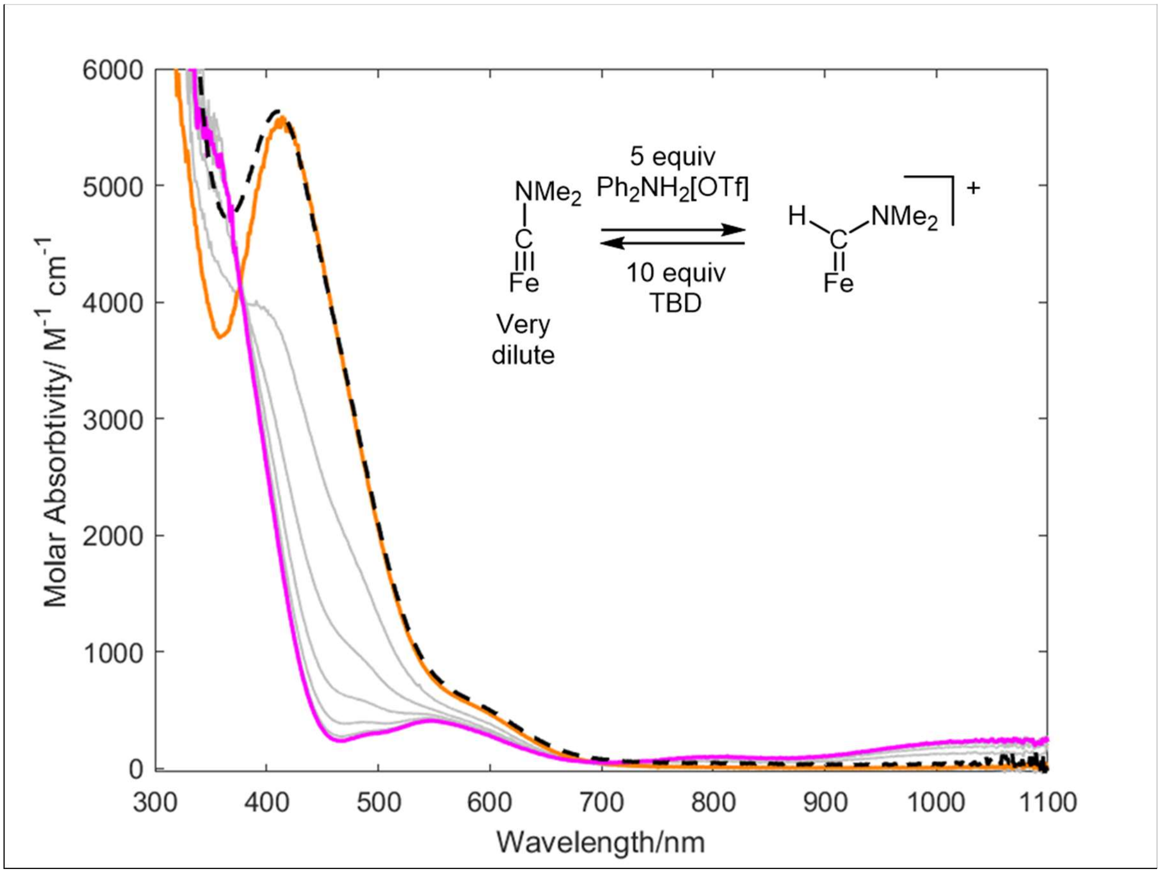

Figure S32. UV-vis monitoring of reaction of $[FeCNMe_2]$ (orange trace) and 5 equiv $[Ph_2NH_2]OTf$ to form $[FeC(H)NMe_2]$ (pink trace). 5 minutes between scans, reaction run at 0 °C. The product was monitored to assure no further reactivity occurred. Excess TBD was added, and the spectrum rapidly regenerated the initially observed spectra of $[FeCNMe_2]$ (Black, dashed trace).

S9.2 Generation of UV-vis spectrum of $[FeC(H)(NMe_2)]^+$ with reductant.

A cuvette of $[FeCNMe_2]$[1] (1.5 mM) in THF was prepared in the glovebox and sealed with a septum. It was brought out of the glovebox with a positive flow of N_2 supplied with a needle during UV-vis monitoring. The cuvette was cooled to −20°C, and after equilibration (10 min), 10 equiv $[Ph_2NH_2]OTf$ in 0.25 mLTHF was added via syringe, followed by 10 equiv $(C_6H_6)_2Cr$ in 0.5 mL THF (also added by syringe). The reaction was monitored, with the absorption maxima at 420 nm associated with $[FeCNMe_2]$ consumed with and new maxima growing in at 790 nm, as well as a feature that grows beyond the detection limit of the spectrometer (1100 nm). These new maxima matched the spectra for the proposed $[FeC(H)(NMe_2)]^+$ formed from the reaction of $[FeCNMe_2]$ with $[Ph_2NH_2]OTf$ (**Figure S32**). High energy transitions were obscured by $(C_6H_6)_2Cr^{0/+}$.

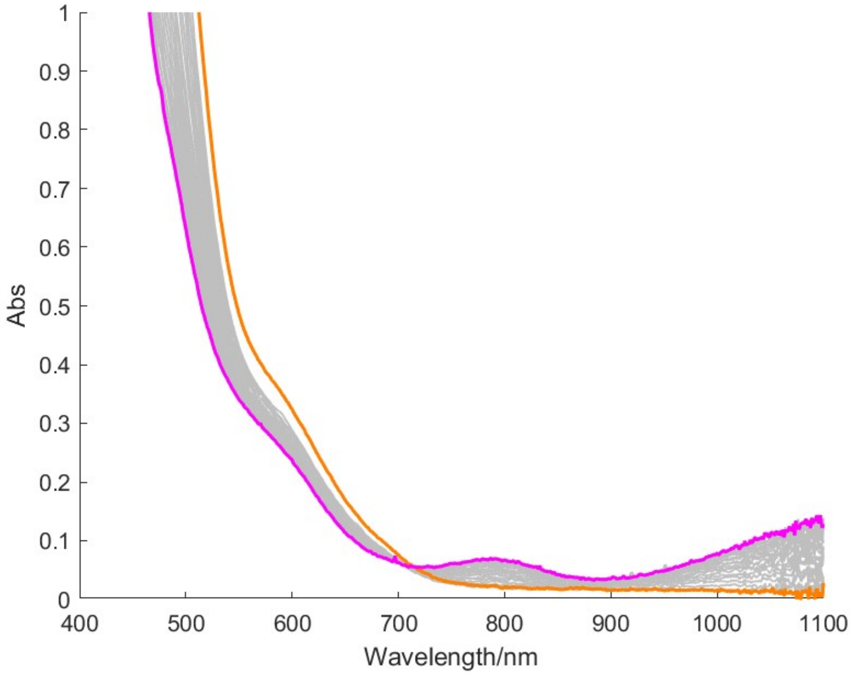

Figure S33. UV-vis monitoring of reaction of $[FeCNMe_2]$ (pink trace) and with 10 equiv $[Ph_2NH_2]OTf$ and 10 equiv $(C_6H_6)_2Cr$ to form $[FeC(H)NMe_2]^+$ (pink trace) at room temperature. 30 seconds between scans.

S9.3 $[FeC(H)(NMe_2)]^+$ generation for NMR experiments

A J. Young NMR tube was loaded with $[FeCNMe_2]$ (5 mM) in d_8-THF (0.5 mL) in the glovebox. $[Ph_2NH_2]OTf$ (25 μmol, 10 equiv) was added as a solid and ^1H NMR was collected. The formation of $[FeCNMe_2]^+$, H_2 and a new species were observed. Characteristic peaks (that do not overlap with acid or $[FeCNMe_2]^+$), for this new species were at 23.4 ppm, 5.08 ppm, -2.00 ppm. Addition of $(C_6H_6)_2Cr$ (25 μmol, 10 equiv) resulted in complete consumption of $[FeCNMe_2]^+$ and an increase concentration of the new species. Over time this species decays to form [FeOTf]. After one week [FeOTf] was the only major paramagnetic Fe-species left. The contents of the tube were cooled to -78°C and transferred to a Schlenk flask, which was subsequently frozen to 77 K. Once frozen the flask was opened to air and KO^{t}Bu was

added as a solid. The volatiles were vacuum transferred onto a receiving flask of 3 ml 1 M HCl in Et_2O. The receiving flask was thawed, and solvent was removed *in vacuo* to leave a solid precipitate. This was taken up in d_6-DMSO with a known amount of 1,3,5-trimethoxybenzene added, with $[Me_2NH_2]Cl$ and $[Me_3NH]Cl$ both detectable as volatile basic products (**Figure S36**).

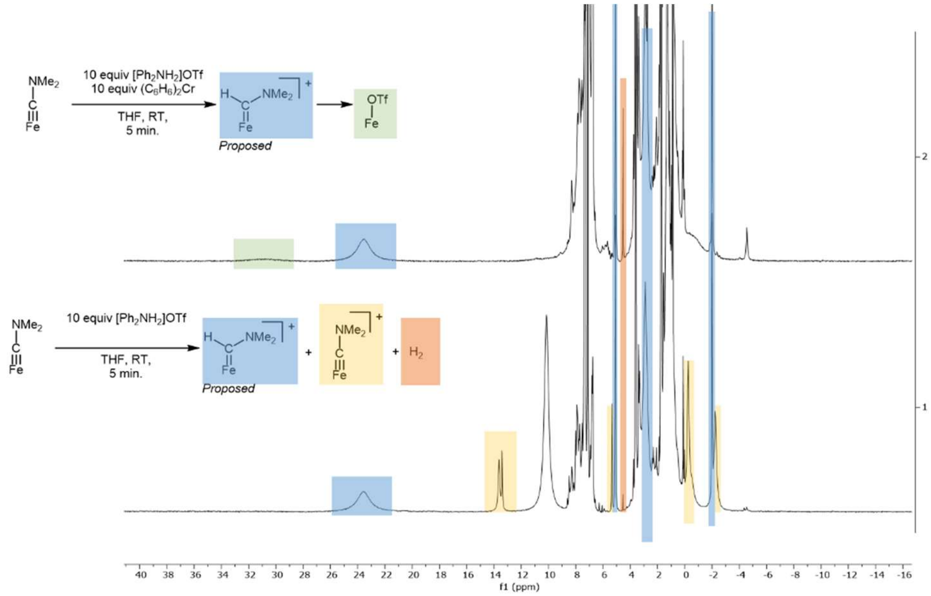

Figure S34. ^{1}H NMR spectra of reaction of $P_3^{Si}FeCNMe_2$ with 10 equiv $[Ph_2NH_2]OTf$ (top) and reaction of $P_3^{Si}FeCNMe_2$ with 10 equiv $[Ph_2NH_2]OTf$ and 10 equiv $(C_6H_6)_2Cr$ (bottom). Peaks diagnostic for $[FeCNMe_2]^+$,[1] [FeOTf][8], H_2,[12] and the proposed $[FeC(H)(NMe_2)]^+$ are highlighted.

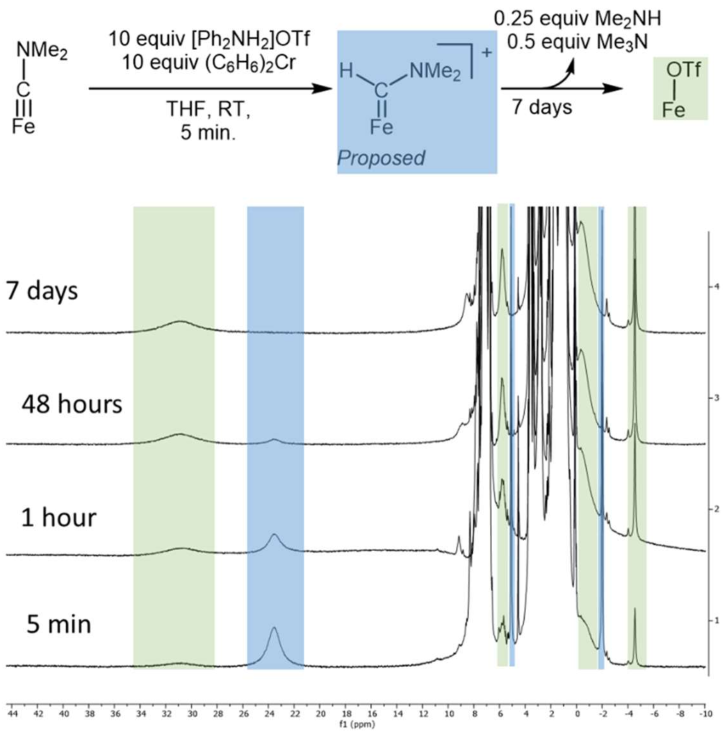

Figure S35. Reaction of $P_3^{Si}FeCNMe_2$ with 10 equiv $[Ph_2NH_2]OTf$ and 10 equiv $(C_6H_6)_2Cr$ (bottom), monitored overtime. Peaks diagnostic for [FeOTf][8] and the proposed $[FeCHNMe_2]$ are highlighted.

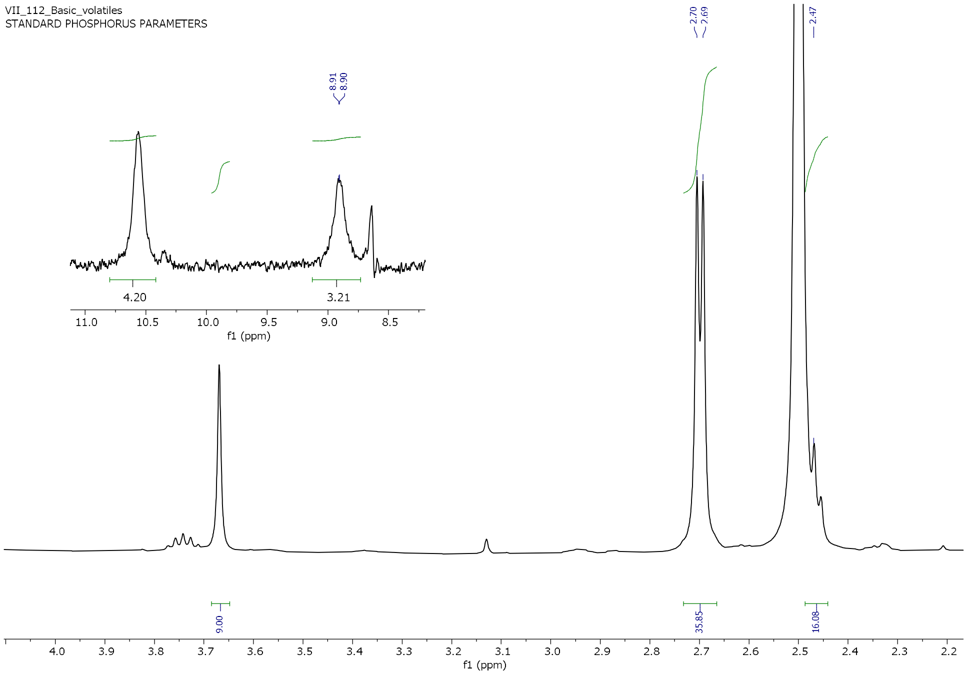

Figure S36. Volatile basic products from the reaction of $P_3^{Si}FeCNMe_2$ with 10 equiv $[Ph_2NH_2]OTf$ and 10 equiv $(C_6H_6)_2Cr$, with $[Me_2NH_2]Cl$ (δ 8.8, br, 2H, δ 2.48, t, J = 6 Hz, 6H) and $[Me_3NH]Cl$ (δ 10.6, br, 2H, δ 2.69, d, J = 5.2 Hz, 9H) products.

S9.4 Mössbauer experiment

The Mössbauer spectrum of this proposed carbene species ($[FeC(H)(NMe_2)]^+$) was generated by reacting $[FeCNMe_2]$ (25 mg, 35 μmol) with 10 equiv $(C_6H_6)_2Cr$ (72 mg, 350 μmol) and 10 equiv $[Ph_2NH_2]OTf$ (95 mg, 350 μmol) in 0.8 mL THF at room temperature for 5 minutes. The reaction was transferred to a Mössbauer cup and frozen at 77 K.

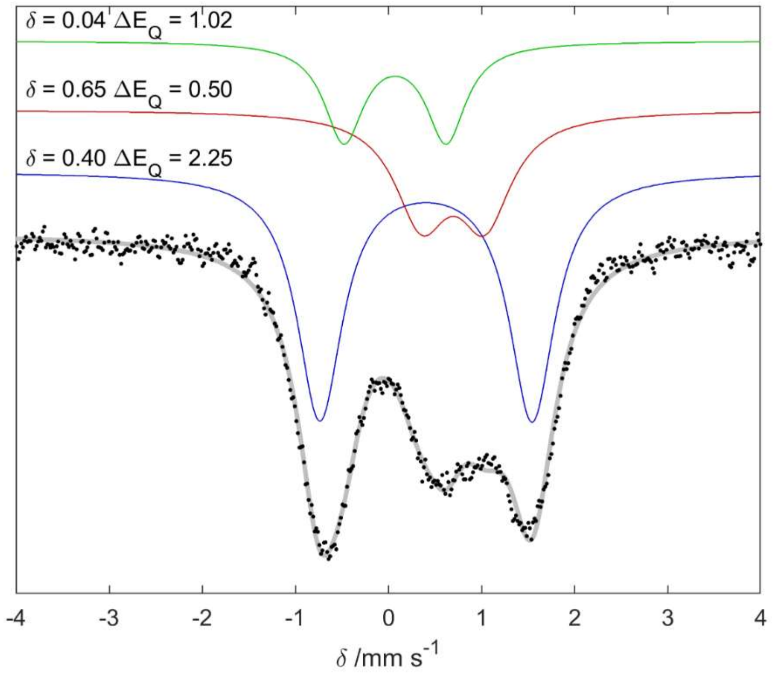

Trace	δ (mm s^{-1})	ΔE_Q (mm s^{-1})	Relative area	Assignment
Blue	0.40±0.01	2.25±0.01	54 %	$[FeC(H)(NMe_2)]^+$
Red	0.65±0.05	0.5±0.1	26%	[FeOTf]
Green	0.04±0.05	1.02±0.1	19 %	$[FeCNMe_2]$

Figure S37. Mössbauer spectrum of reaction of $P_3^{Si}FeCNMe_2$ with 10 equiv each of $(C_6H_6)_2Cr$ and $[Ph_2NH_2]OTf$. The major species (blue) is proposed to be $[FeC(H)(NMe_2)]^+$, with the other species matching previously characterized [FeOTf][10] and $[FeCNMe_2]$.[1]

S10 Derivation of estimated BDFE for early N−H bonds

To derive the early N−H bond Bond Dissociation Free Energies (BDFE) we have used previously measured values,[10] but with updated C_G constants, which have since our initial publication been measured in THF (C_G = 59.9 kcal mol^{-1}),[13] allowing us to derive BDFEs and not only BDEs. For clarity the thermochemical parameters previously measured are presented again in **Table S5.** The BDFEs are derived using these values.

In addition, we derive the $BDFE_{eff}$ for the combination of $(C_6H_6)_2Cr$/$[Ph_2NH_2]OTf$ used as a reference for the ΔG derived for H^+/e^- addition in **figure 7** in the main text.

Table S5. E_{ox} and pK_a for $[FeCNH_x]$ species and $(C_6H_6)_2Cr$ and $[Ph_2NH_2]OTf$ used to derive $BDFE_{N\text{-}H}$ and $BDFE_{eff}$.

Compound	E_{ox} (vs $Fc^{+/0}$ in THF)	pK_a (in THF)
[FeCN]	−0.38 V	
$[FeCNH]^+$	−0.17 V	5.6
[FeCNH]	−1.27 V	
$[FeCN(Me)H]^+$		7.1
[FeCN(Me)H]	−1.27 V	
[FeCNMe]	−1.31 V	
$(C_6H_6)_2Cr$	−1.22 V	
$[Ph_2NH_2]OTf$		3.2

Estimation of the N–H BDFE of $[FeCNH]^+$:

$$
\begin{array}{ll}
[FeCNH]^+ \rightleftharpoons [FeCN] + H^+ & 1.37(pK_a([FeCNH]^+)) = 7.7\ \text{kcal mol}^{-1} \\
[FeCN] \rightleftharpoons [FeCN]^+ + e^- & 23.06(E_{ox}([FeCN])) = -8.8\ \text{kcal mol}^{-1} \\
\underline{H^+ + e^- \rightleftharpoons H^\bullet} & \underline{C_G = 59.9\ \text{kcal mol}^{-1}} \\
[FeCNH]^+ \rightleftharpoons [FeCN]^+ + H^\bullet & BDFE_{N\text{–}H}([FeCNH]^+) = 58.8\ \text{kcal mol}^{-1}
\end{array}
$$

Estimation of the N–H BDFE of [FeCNH]:

$$
\begin{array}{ll}
[FeCNH] \rightleftharpoons [FeCNH]^+ + e^- & 23.06(E_{ox}([FeCN])) = -29.3\ \text{kcal mol}^{-1} \\
[FeCNH]^+ \rightleftharpoons [FeCN] + H^+ & 1.37(pK_a([FeCNH]^+)) = 7.7\ \text{kcal mol}^{-1} \\
\underline{H^+ + e^- \rightleftharpoons H^\bullet} & \underline{C_G = 59.9\ \text{kcal mol}^{-1}} \\
[FeCNH] \rightleftharpoons [FeCN] + H^\bullet & BDFE_{N\text{–}H}([FeCNH]) = 38.3\ \text{kcal mol}^{-1}
\end{array}
$$

Estimation of the N–H BDFE of $[FeCN(Me)H]^+$:

$$
\begin{array}{ll}
[FeCN(Me)H]^+ \rightleftharpoons [FeCNMe] + H^+ & 1.37(pK_a([FeCN(Me)H]^+)) = 9.7\ \text{kcal mol}^{-1} \\
[FeCNMe] \rightleftharpoons [FeCNMe]^+ + e^- & 23.06(E_{ox}([FeCNMe])) = -30.2\ \text{kcal mol}^{-1} \\
\underline{H^+ + e^- \rightleftharpoons H^\bullet} & \underline{C_G = 59.9\ \text{kcal mol}^{-1}} \\
[FeCN(Me)H]^+ \rightleftharpoons [FeCNMe]^+ + H^\bullet & BDFE_{N\text{–}H}([FeCN(Me)H]^+) = 39.4\ \text{kcal mol}^{-1}
\end{array}
$$

Estimation of the N–H BDFE of [FeCN(Me)H]:

[FeCN(Me)H] ⇌ $[FeCN(Me)H]^+ + e^-$	$23.06(E_{ox}([FeCN(Me)H])) = -29.3$ kcal mol^{-1}
$[FeCN(Me)H]^+$ ⇌ [FeCNMe] + H^+	$1.37(pK_a([FeCN(Me)H]^+)) = 9.7$ kcal mol^{-1}
$H^+ + e^-$ ⇌ $H^\bullet$	$C_G = 59.9$ kcal mol^{-1}
[FeCN(Me)H] ⇌ [FeCNMe] + $H^\bullet$	$BDFE_{N\text{–}H}([FeCN(Me)H]) = 40.3$ kcal mol^{-1}

Estimation of the $BDFE_{eff}$ of $(C_6H_6)_2Cr/[Ph_2NH_2]OTf$:

$(C_6H_6)_2Cr$ ⇌ $(C_6H_6)_2Cr^+ + e^-$	$23.06(E_{ox}((C_6H_6)_2Cr)) = -28.1$ kcal mol^{-1}
$[Ph_2NH_2]^+$ ⇌ $[Ph_2NH] + H^+$	$1.37(pK_a([Ph_2NH_2]^+)) = 4.4$ kcal mol^{-1}
$H^+ + e^-$ ⇌ $H^\bullet$	$C_G = 59.9$ kcal mol^{-1}
$(C_6H_6)_2Cr/[Ph_2NH_2]OTf$ ⇌ $[(C_6H_6)_2Cr]OTf/Ph_2NH + H^\bullet$	$BDFE_{eff} = 36.2$ kcal mol^{-1}

S11 Computational Methods

S11.1 General overview of computational details

As stated in the main text for our computational studies we employed the TPSS functional[14] and a def2-TZVP basis set on transition metals and a def2-SVP basis set on all other atoms,[15] which we have previously demonstrated accurately replicate BDFEs on similar platforms.[16] The ORCA open source software package was used.[17]

The numerical frequencies of the minimized structures were calculated to assure that these structures represented local minima and not saddle points. Entropic and internal energy contributions were calculated at 195 K, 253 K, 273 K and 293 K to computationally derive the ΔG_f at these temperatures.

For the analysis in **Figure 6**, comparison of the lowest energy isomers are presented in **Tables S6, S7** and **S8**. Key bond lengths and their comparison to relevant crystallographic data are presented in **Table S9**.

For the analysis in **Figure 7**, the energy of each pathway is presented in **Figure S38** and **S39**. Final step(s) to convert the final intermediate in pathways *i-iii* (as defined in **Figure 7**) to an on-path species $[FeCNH]^{+/0}$ is presented in **Figure S39**. The calculations to derive the ΔG in **Figure 7** and **Figure S38**, **S39** are summarized in **Tables S10-S14**.

The minimized structures are provided in a separate .txt file.

S.11.2 Treatment of $H^{\bullet}$

The treatment of $S(H^{\bullet})$ in calculations can be a source of systematic error in DFT calculations, therefore it is useful to detail how this value has been derived. We have taken an *ab initio* approach to $S(H^{\bullet})$ using the !PRINTTHERMOCHEM function in ORCA 5.0[18] to calculate the translational entropy and internal energy of $H^{\bullet}$ at temperatures 195 K, 253 K, 273 K and 293 K. These values are used directly to derive the $\Delta G_f(H^{\bullet})$ values as presented in **Table S9**.

S11.2 Calculating ΔG for bond formations in figure 7

In the analysis of step g in **Figure 7** we use the computations presented in **Figures S38-S39** and **Tables S10-S14** to guide the discussion. However, for clarity for bond formation steps we reference these H-atom transfers from the addition of H^+ from $[Ph_2NH_2]OTf$ and e^- from $(C_6H_6)_2Cr$ to the $BDFE_{eff}$ of this reagent combination, derived in **section S10** to be 36.2 kcal mol^{-1}.

For example, for the addition of an H-atom to $[FeC(H)(NH_2)]^+$ to $[FeC(H)(NH_3)]^+$ the $BDFE_{N\text{-}H}$ of this bond is 14 kcal mol^{-1} (**Table S11**). The ΔG as reported in **Figure 7** and **Figures S38-S39** is then given as $\Delta G = \mathrm{BDFE_{eff}} - \mathrm{BDFE_{N-H}}$ = 36-14 = +<u>22 kcal mol^{-1}</u>

Table S6. Table of experimental[10] and computationally derived bond dissociation free energies and comparison to relevant experimental values.

	$BDFE_{195K}$ (kcal mol^{-1})	$BDFE_{253K}$ (kcal mol^{-1})	$BDFE_{273K}$ (kcal mol^{-1})	$BDFE_{298K}$ (kcal mol^{-1})	$BDFE_{298\ K,\ exp}$ (kcal mol^{-1})
$P_3^{Si}FeCNH_2^+ \rightarrow P_3^{Si}FeCNH^+ + H^\bullet$	42	40	39	38	
$P_3^{Si}FeCHNH_2^+ \rightarrow P_3^{Si}FeCNH^+_2 + H^\bullet$	55	53	53	52	
$P_3^{Si}FeCHNH_2 \rightarrow P_3^{Si}FeCNH_2 + H^\bullet$	49	48	47	47	
$P_3^{Si}FeCNH_2 \rightarrow P_3^{Si}FeCNH + H^\bullet$	38	36	35	35	
$P_3^{Si}FeCN(Me)H^+ \rightarrow P_3^{Si}FeCNMe^+ + H^\bullet$					41
$P_3^{Si}FeCN(Me)H \rightarrow P_3^{Si}FeCNMe + H^\bullet$					40

Table S7. Computationally derived free energies of intermediates of ($P_3^{Si}FeCNH_2 + H^\bullet$).

Compound	Spin state	$\Delta G_{f,195}$ (Eh)	$\Delta G_{f,253}$ (Eh)	$\Delta G_{f,273}$ (Eh)	$\Delta G_{f,298}$ (Eh)	$\Delta G_{rel,195}$ (kcal mol^{-1})	$\Delta G_{rel,253}$ (kcal mol^{-1})	$\Delta G_{rel,273}$ (kcal mol^{-1})	$\Delta G_{rel,298}$ (kcal mol^{-1})
$P_3^{Si}Fe(CNH_3)^+$ species									
$P_3^{Si}FeC(H)(NH_2)^+$	triplet	-4076.57	-4076.59	-4076.59	-4076.60	0.0	0.0	0.0	0.0
$P_3^{Si}FeC(H)(NH_2)^+$	singlet	-4076.55	-4076.57	-4076.58	-4076.59	9.3	9.5	9.6	9.7
$P_3^{Si}FeC(H)(NH_2)^+$	quintet	-4076.51	-4076.53	-4076.53	-4076.54	39.1	38.7	38.6	38.4
$P_3^{Si}FeCNH_3^+$	singlet	-4076.50	-4076.51	-4076.52	-4076.53	45.1	45.6	45.7	46.0
$P_3^{Si}FeCNH_3^+$	triplet	-4076.46	-4076.48	-4076.49	-4076.50	65.5	65.7	65.8	65.9
$P_3^{Si}Fe(H)CNH_2^+$	singlet	-4076.56	-4076.57	-4076.58	-4076.59	6.9	7.7	7.8	8.0
$P_3^{Si}FeC^+$	singlet	-4019.92	-4019.94	-4019.94	-4019.95	-	-	-	-
$P_3^{Si}FeC^+ + NH_3$	singlet	-4076.48	-4076.51	-4076.51	-4076.52	53.3	51.2	50.5	49.6
$P_3^{Si}Fe(CNH_3)$ species									
$P_3^{Si}FeC(H)(NH_2)$	doublet	-4076.73	-4076.75	-4076.75	-4076.76	0.0	0.0	0.0	0.0
$P_3^{Si}FeC(H)(NH_2)$	quartet	-4076.68	-4076.70	-4076.71	-4076.72	28.6	28.4	28.4	28.3
$P_3^{Si}FeCNH_3$	doublet	-4076.62	-4076.64	-4076.65	-4076.66	66.1	66.0	66.0	65.9
$P_3^{Si}Fe(H)CNH_2$	doublet	-4076.70	-4076.72	-4076.72	-4076.73	18.5	19.1	19.1	19.0
$P_3^{Si}FeC$	doublet	-4020.10	-4020.12	-4020.13	-4020.14	-	-	-	-
$P_3^{Si}FeC + NH_3$	doublet	-4076.67	-4076.69	-4076.70	-4076.71	39.2	36.9	36.0	35.0

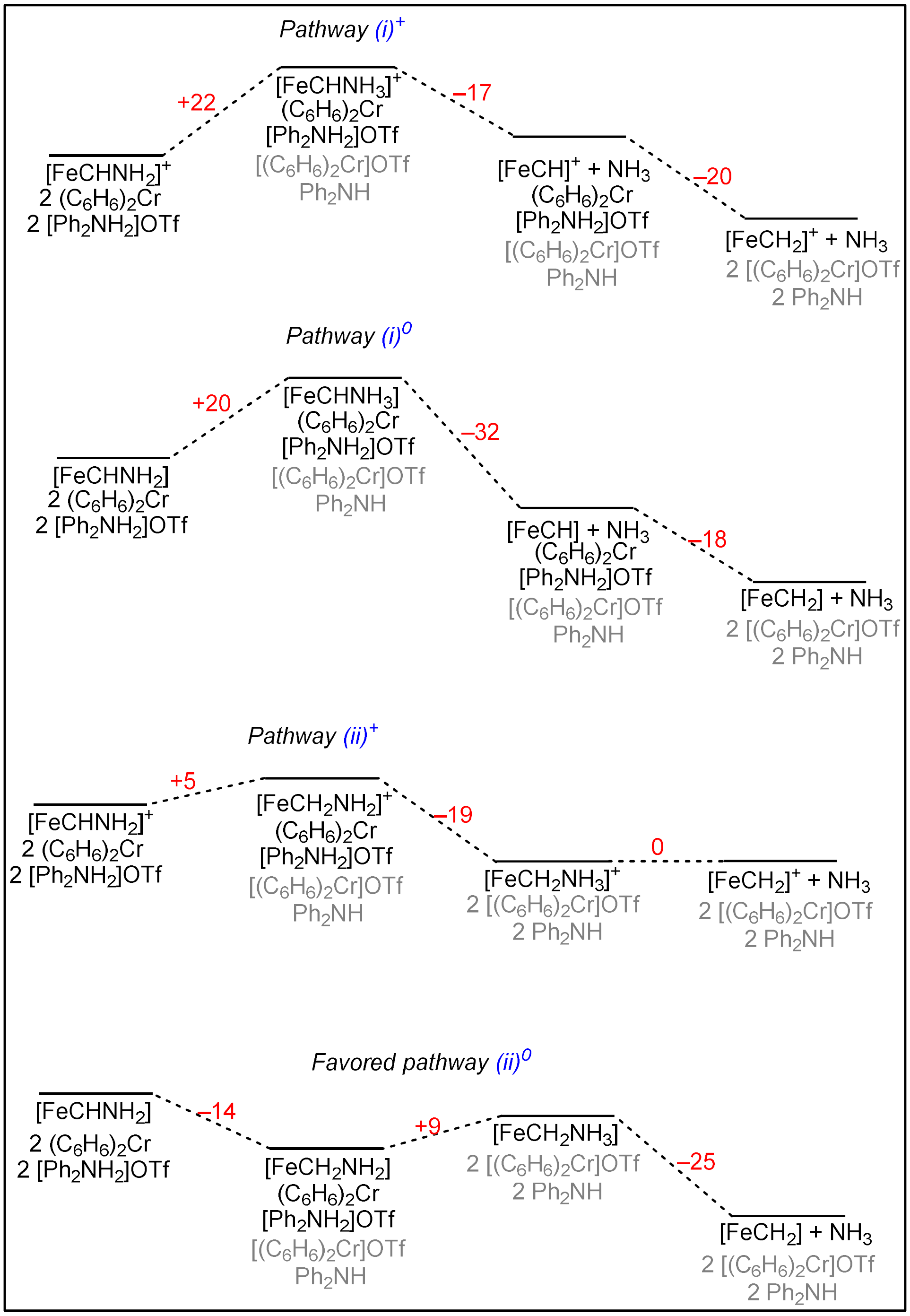

Figure S38. Thermodynamic barriers for CH_4 and NH_3 selective pathways *i-ii*.

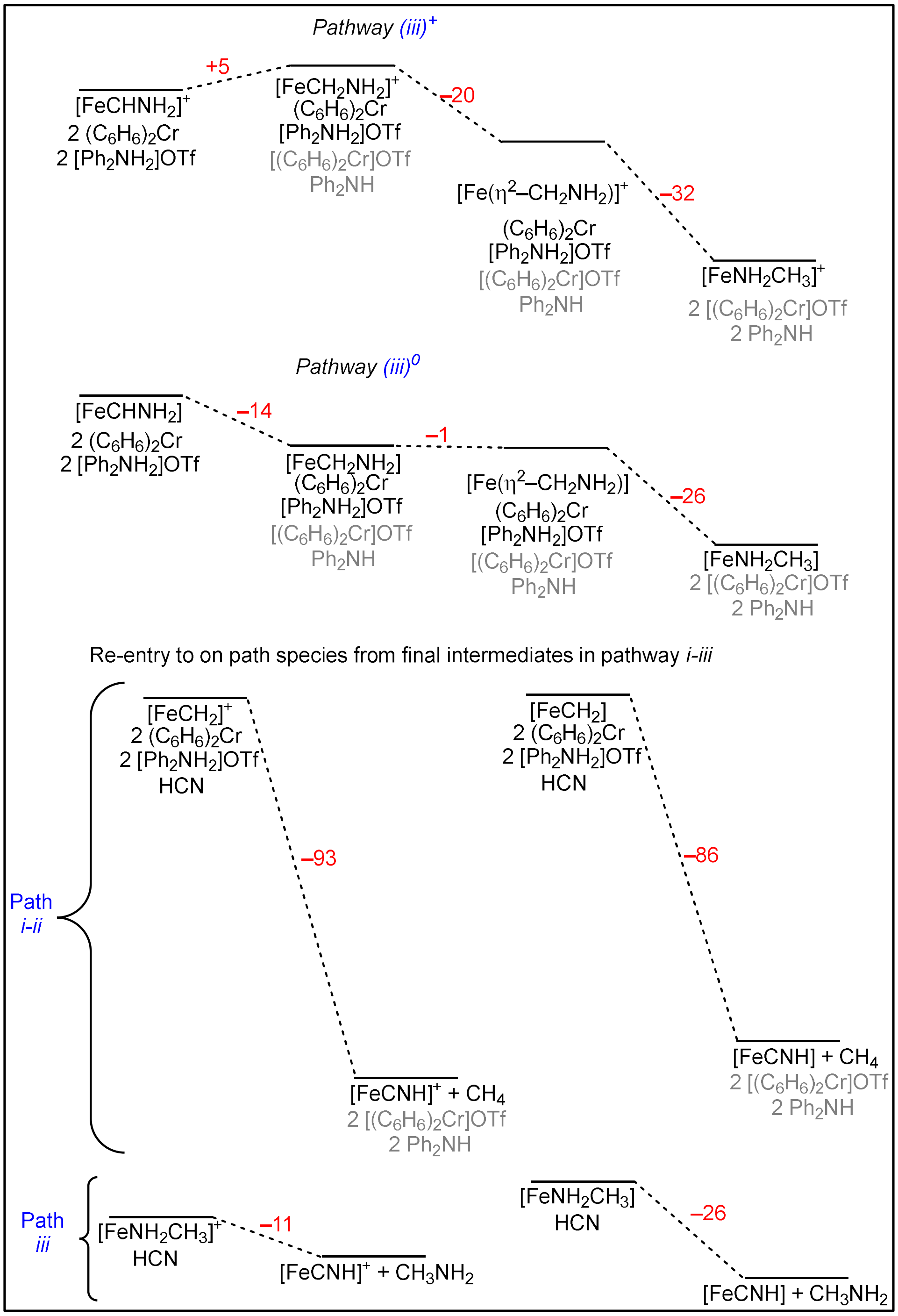

Figure S39. Thermodynamic barriers for CH_3NH_2 selective pathways *iii* and thermodynamic barriers for release of substrate, and rebinding of CN^-.

Table S8. Computationally derived free energies of early intermediates of $P_3^{Si}FeCN$ reduction.

Compound	Spin state	$\Delta G_{f,195}$ (Eh)	$\Delta G_{f,253}$ (Eh)	$\Delta G_{f,273}$ (Eh)	$\Delta G_{f,298}$ (Eh)
$P_3^{Si}FeCNH^+$	triplet	-4075.40	-4075.42	-4075.43	-4075.44
$P_3^{Si}FeCNH$	doublet	-4075.58	-4075.60	-4075.60	-4075.61
$P_3^{Si}FeCNH_2^+$	doublet	-4075.98	-4075.99	-4076.00	-4076.01
$P_3^{Si}FeCNH_2$	singlet	-4076.14	-4076.16	-4076.17	-4076.18

Table S9. Experimentally[1,8,10] and computationally derived key bond lengths and comparison with known crystallographic data. In general, the Fe−P and Fe−Si bonds are very reliably reproduced computationally. Increased error is observed in the C−N bond. This is likely partially attributable to the use of methylated analogues (due to the instability of the compounds) and the hydrogen bonds formed to N−H in $P_3^{Si}FeCNH^+$ and $P_3^{Si}FeCNH_2^+$. The Fe−C bond length is consistently overestimated by about 0.05 Å by DFT.

Compound		C−N (Å)	Fe−C (Å)	Fe−P_{ave} (Å)	Fe−Si (Å)	<Fe−C−N
$P_3^{Si}FeCNH^+$	Computed	1.186	1.857	2.368	2.331	177.1
$P_3^{Si}FeCNH^+$	Crystal.	1.144	1.913	2.384	2.305	178.4
$P_3^{Si}FeCNH$	Computed	1.228	1.774	2.266	2.289	175.6
$P_3^{Si}FeCNMe$	Crystal	1.186	1.821	2.273	2.273	177.6
$P_3^{Si}FeCNH_2^+$	Computed	1.317	1.684	2.316	2.358	176.5
$P_3^{Si}FeCNH_2^+$	Crystal	1.223	1.799	2.314	2.311	174.2
$P_3^{Si}FeCNMe_2^+$	Crystal	1.310	1.736	2.364	2.358	173.8
$P_3^{Si}FeCNH_2$	Computed	1.337	1.675	2.214	2.310	179.1
$P_3^{Si}FeCNMe_2$	Crystal	1.328	1.710	2.230	2.305	177.8
$P_3^{Si}FeCH^+$	Computed		1.65	2.30	2.38	
$P_3^{Si}FeCMe^+$	Crystal	-	1.70	2.34	2.39	-
$P_3^{Si}FeCH$	Computed		1.65	2.21	2.32	
$P_3^{Si}FeCMe$	Crystal	-	1.67	2.25	2.33	-

Table S10. Computationally derived energies of small molecules relevant to calculations.

Compound	Spin state	$\Delta G_{f,195}$ (Eh)	$\Delta G_{f,253}$ (Eh)	$\Delta G_{f,273}$ (Eh)	$\Delta G_{f,298}$ (Eh)
HCN	singlet	-93.47	-93.48	-93.48	-93.48
$MeNH_2$	singlet	-95.86	-95.87	-95.87	-95.87
CH_4	singlet	-40.51	-40.51	-40.51	-40.51
NH_3	singlet	-56.57	-56.57	-56.57	-56.57
H•	doublet	-0.5062	-0.5086	-0.5094	-0.5105

Table S11. Table of computationally derived energies of isomers and spin states of $P_3^{Si}Fe(CNH_4)$. Values derived here used in figure 7, S37 and S38.

Compound	Spin state	$\Delta G_{f,195}$ (Eh)	$\Delta G_{f,253}$ (Eh)	$\Delta G_{f,273}$ (Eh)	$\Delta G_{f,298}$ (Eh)	$\Delta G_{rel,195}$ (kcal mol^{-1})	$\Delta G_{rel,253}$ (kcal mol^{-1})	$\Delta G_{rel,273}$ (kcal mol^{-1})	$\Delta G_{rel,298}$ (kcal mol^{-1})
$P_3^{Si}FeCHNH_2^+ + H\cdot$	-	-4077.07	-4077.10	-4077.10	-4077.11	0	0	0	0
$P_3^{Si}FeCHNH_3^+$	doublet	-4077.10	-4077.12	-4077.13	-4077.14	-17	-15	-14	-14
$P_3^{Si}FeCH^+$	doublet	-4020.55	-4020.57	-4020.58	-4020.59	-	-	-	-
$P_3^{Si}FeCH^+ + NH_3$	doublet	-4077.12	-4077.14	-4077.15	-4077.16	-29	-30	-30	-30
$P_3^{Si}FeCH_2NH_2^+$	quartet	-4077.13	-4077.15	-4077.15	-4077.16	-34	-32	-32	-31
$P_3^{Si}Fe(\eta^2\text{-}CH_2NH_2)^+$	doublet	-4077.16	-4077.18	-4077.19	-4077.20	-54	-52	-52	-51

Table S12. Table of computationally derived energies of isomers and spin states of $P_3^{Si}Fe(CNH_4)$. Values derived here used in figure 7, S37 and S38.

Compound	Spin state	$\Delta G_{f,195}$ (Eh)	$\Delta G_{f,253}$ (Eh)	$\Delta G_{f,273}$ (Eh)	$\Delta G_{f,298}$ (Eh)	$\Delta G_{rel,195}$ (kcal mol^{-1})	$\Delta G_{rel,253}$ (kcal mol^{-1})	$\Delta G_{rel,273}$ (kcal mol^{-1})	$\Delta G_{rel,298}$ (kcal mol^{-1})
$P_3^{Si}FeCHNH_2 + H\cdot$		-4077.24	-4077.26	-4077.26	-4077.27	0	0	0	0
$P_3^{Si}FeCHNH_3$ constrained	singlet	-4077.26	-4077.28	-4077.29	-4077.30	-17	-17	-16	-16
$P_3^{Si}FeCH$	singlet	-4020.74	-4020.76	-4020.77	-4020.78	-	-	-	-
$P_3^{Si}FeCH + NH_3$	singlet	-4077.31	-4077.33	-4077.34	-4077.35	-47	-48	-48	-48
$P_3^{Si}FeCH_2NH_2$	triplet	-4077.32	-4077.34	-4077.34	-4077.35	-52	-51	-50	-50
$P_3^{Si}Fe(\eta^2\text{-}CH_2NH_2)$	singlet	-4077.32	-4077.34	-4077.35	-4077.36	-54	-52	-52	-51

Table S13. Table of computationally derived energies of isomers and spin states of $P_3^{Si}Fe(CNH_5)^+$. Values derived here used in figure 7, S37 and S38.

Compound	Spin state	$\Delta G_{f,195}$ (Eh)	$\Delta G_{f,253}$ (Eh)	$\Delta G_{f,273}$ (Eh)	$\Delta G_{f,298}$ (Eh)	$\Delta G_{rel,195}$ (kcal mol^{-1})	$\Delta G_{rel,253}$ (kcal mol^{-1})	$\Delta G_{rel,273}$ (kcal mol^{-1})	$\Delta G_{rel,298}$ (kcal mol^{-1})
$P_3^{Si}Fe(\eta^2\text{-}CH_2NH_2)^+ + H.$		-4077.67	-4077.69	-4077.70	-4077.71	0	0	0	0
$P_3^{Si}FeCH_2NH_3^+$	triplet	-4077.73	-4077.75	-4077.75	-4077.76	-37	-36	-36	-35
$P_3^{Si}FeCH_2^+$	triplet	-4021.16	-4021.17	-4021.18	-4021.19	-	-	-	-
$P_3^{Si}FeCH_2^+$	singlet	-4021.15	-4021.17	-4021.18	-4021.19	-	-	-	-
$P_3^{Si}FeCH_2^+ + NH_3$	triplet	-4077.72	-4077.74	-4077.75	-4077.76	-34	-35	-35	-36
$P_3^{Si}FeNH_2CH_3^+$	triplet	-4077.78	-4077.80	-4077.80	-4077.81	-70	-69	-68	-68

Table S14. Table of computationally derived energies of isomers and spin states of $P_3^{Si}Fe(CNH_5)$. Values derived here used in figure 7, S37 and S38.

Compound	Spin state	$\Delta G_{f,195}$ (Eh)	$\Delta G_{f,253}$ (Eh)	$\Delta G_{f,273}$ (Eh)	$\Delta G_{f,298}$ (Eh)	$\Delta G_{rel,195}$ (kcal mol^{-1})	$\Delta G_{rel,253}$ (kcal mol^{-1})	$\Delta G_{rel,273}$ (kcal mol^{-1})	$\Delta G_{rel,298}$ (kcal mol^{-1})
$P_3^{Si}Fe(\eta^2\text{-}CH_2NH_2)$ + H.		-4077.83	-4077.85	-4077.86	-4077.87	0	0	0	0
$P_3^{Si}FeCH_2NH_3$	doublet	-4077.87	-4077.89	-4077.90	-4077.91	-28	-27	-27	-26
$P_3^{Si}FeCH_2$	doublet	-4021.34	-4021.36	-4021.37	-4021.38	-	-	-	-
$P_3^{Si}FeCH_2$ + NH_3	doublet	-4077.91	-4077.93	-4077.94	-4077.95	-50	-51	-51	-52
$P_3^{Si}FeNH_2CH_3$	doublet	-4077.93	-4077.95	-4077.96	-4077.97	-64	-63	-63	-62

S11.3 Example input file – optimization and frequency job

```
! tpss opt Numfreq def2-tzvpp d3zero VerySlowConv

%pal
nprocs 8
end

%freq
temp 195
end

%maxcore 1000

%scf
maxiter 500
end

* XYZFILE 1 3 inputfile.xyz #Numbers: charge state spin state
```

S12 References

(1) Rittle, J.; Peters, J. C. Proton-Coupled Reduction of an Iron Cyanide Complex to Methane and Ammonia. *Angew. Chem. Int. Ed.* **2016**, *55* (40), 12262–12265. https://doi.org/10.1002/anie.201606366.

(2) Anderson, J. S.; Moret, M.-E.; Peters, J. C. Conversion of Fe–NH_2 to Fe–N_2 with Release of NH_3. *J. Am. Chem. Soc.* **2013**, *135* (2), 534–537. https://doi.org/10.1021/ja307714m.

(3) Betley, T. A.; Peters, J. C. A Tetrahedrally Coordinated L_3Fe–N_x Platform That Accommodates Terminal Nitride (Fe^{IV}≡N) and Dinitrogen (Fe^{I}–N_2–Fe^{I}) Ligands. *J. Am. Chem. Soc.* **2004**, *126* (20), 6252–6254.

(4) Chalkley, M. J.; Del Castillo, T. J.; Matson, B. D.; Roddy, J. P.; Peters, J. C. Catalytic N_2-to-NH_3 Conversion by Fe at Lower Driving Force: A Proposed Role for Metallocene-Mediated PCET. *ACS Cent. Sci.* **2017**, *3* (3), 217–223. https://doi.org/10.1021/acscentsci.7b00014.

(5) Del Castillo, T. J.; Thompson, N. B.; Peters, J. C. A Synthetic Single-Site Fe Nitrogenase: High Turnover, Freeze-Quench ^{57}Fe Mössbauer Data, and a Hydride Resting State. *J. Am. Chem. Soc.* **2016**, *138* (16), 5341–5350. https://doi.org/10.1021/jacs.6b01706.

(6) Erhardt, S.; Grushin, V. V.; Kilpatrick, A. H.; Macgregor, S. A.; Marshall, W. J.; Roe, D. C. Mechanisms of Catalyst Poisoning in Palladium-Catalyzed Cyanation of Haloarenes. Remarkably Facile C−N Bond Activation in the $[(Ph_3P)_4Pd]/[Bu_4N]^+ CN^-$ System. *J. Am. Chem. Soc.* **2008**, *130* (14), 4828–4845. https://doi.org/10.1021/ja078298h.

(7) Chalkley, M. J.; Del Castillo, T. J.; Matson, B. D.; Peters, J. C. Fe-Mediated Nitrogen Fixation with a Metallocene Mediator: Exploring PKa Effects and Demonstrating Electrocatalysis. *J. Am. Chem. Soc.* **2018**, *140* (19), 6122–6129. https://doi.org/10.1021/jacs.8b02335.

(8) Citek, C.; Oyala, P. H.; Peters, J. C. Mononuclear Fe(I) and Fe(II) Acetylene Adducts and Their Reductive Protonation to Terminal Fe(IV) and Fe(V) Carbynes. *J. Am. Chem. Soc.* **2019**, *141* (38), 15211–15221. https://doi.org/10.1021/jacs.9b06987.

(9) Lee, Y.; Kinney, R. A.; Hoffman, B. M.; Peters, J. C. A Nonclassical Dihydrogen Adduct of S = 1/2 Fe(I). *J. Am. Chem. Soc.* **2011**, *133* (41), 16366–16369. https://doi.org/10.1021/ja207003m.

(10) Rittle, J.; Peters, J. C. N–H Bond Dissociation Enthalpies and Facile H Atom Transfers for Early Intermediates of Fe–N_2 and Fe–CN Reductions. *J. Am. Chem. Soc.* **2017**, *139* (8), 3161–3170. https://doi.org/10.1021/jacs.6b12861.

(11) Lee, K. J.; Elgrishi, N.; Kandemir, B.; Dempsey, J. L. Electrochemical and Spectroscopic Methods for Evaluating Molecular Electrocatalysts. *Nat. Rev. Chem.* **2017**, *1* (5). https://doi.org/10.1038/s41570-017-0039.

(12) Fulmer, G. R.; Miller, A. J. M.; Sherden, N. H.; Gottlieb, H. E.; Nudelman, A.; Stoltz, B. M.; Bercaw, J. E.; Goldberg, K. I. NMR Chemical Shifts of Trace Impurities: Common Laboratory Solvents, Organics, and Gases in Deuterated Solvents Relevant to the Organometallic Chemist. *Organometallics* **2010**, *29* (9), 2176–2179. https://doi.org/10.1021/om100106e.

(13) Agarwal, R. G.; Coste, S. C.; Groff, B. D.; Heuer, A. M.; Noh, H.; Parada, G. A.; Wise, C. F.; Nichols, E. M.; Warren, J. J.; Mayer, J. M. Free Energies of Proton-Coupled Electron Transfer Reagents and Their Applications. *Chem. Rev.* **2022**, *122* (1), 1–49. https://doi.org/10.1021/acs.chemrev.1c00521.

(14) Tao, J.; Perdew, J. P.; Staroverov, V. N.; Scuseria, G. E. Climbing the Density Functional Ladder: Nonempirical Meta-Generalized Gradient Approximation Designed for Molecules and Solids. *Phys. Rev. Lett.* **2003**, *91* (14), 146401–146404.

(15) Weigend, F. Accurate Coulomb-Fitting Basis Sets for H to Rn. *Phys. Chem. Chem. Phys.* **2006**, *8* (9), 1057–1065. https://doi.org/10.1039/B515623H.

(16) Matson, B. D.; Peters, J. C. Fe-Mediated HER vs N_2RR: Exploring Factors That Contribute to Selectivity in $P_3^EFe(N_2)$ (E = B, Si, C) Catalyst Model Systems. *ACS Catal.* **2018**, *8* (2), 1448–1455. https://doi.org/10.1021/acscatal.7b03068.

(17) Neese, F.; Wennmohs, F.; Becker, U.; Riplinger, C. The ORCA Quantum Chemistry Program Package. *J. Chem. Phys.* **2020**, *152* (22), 224108. https://doi.org/10.1063/5.0004608.

(18) *Thermodynamics — ORCA tutorials 5.0 documentation*. https://www.orcasoftware.de/tutorials_orca/prop/thermo.html (accessed 2023-10-22).
